# Dysivillosins A–D, Unusual Anti-allergic Meroterpenoids from the Marine Sponge *Dysidea villosa*

**DOI:** 10.1038/s41598-017-04021-z

**Published:** 2017-08-21

**Authors:** Wei-Hua Jiao, Bao-Hui Cheng, Guo-Hua Shi, Guo-Dong Chen, Bin-Bin Gu, Yong-Jun Zhou, Li-Li Hong, Fan Yang, Zhi-Qiang Liu, Shu-Qi Qiu, Zhi-Gang Liu, Ping-Chang Yang, Hou-Wen Lin

**Affiliations:** 1 Research Center for Marine Drugs, State Key Laboratory of Oncogenes and Related Genes, Department of Pharmacy, Ren Ji Hospital, School of Medicine, Shanghai Jiao Tong University, Shanghai, 200127 China; 2 Shenzhen Key Laboratory of ENT, Longgang ENT hospital & Institute of ENT, Shenzhen, 518172 China; 3 Institute of Traditional Chinese Medicine & Natural Products, College of Pharmacy, Jinan University, Guangzhou, 510632 China; 4 Shenzhen Key Laboratory of Allergy & Immunology, Shenzhen University School of Medicine, Shenzhen, 518060 China

## Abstract

Four unusual meroterpenoids, dysivillosins A–D (**1**–**4**), were isolated from an organic extract of the marine sponge *Dysidea villosa* collected from the South China Sea. Their planar structures were determined by 1D and 2D NMR and HRESIMS techniques, while the relative and absolute configurations were elucidated by NOESY experiments and comparison between the calculated and experimental ECD spectra. To the best of our knowledge, dysivillosins A–D are the first examples of terpene-polyketide-pyridine hybrid metabolites from the nature. Anti-allergic activity evaluation showed that compounds **1**–**4** potently inhibited the release of β-hexosaminidase, a marker of degranulation, in a dose-dependent manner with IC_50_ values of 8.2–19.9 μM. Additionally, the four meroterpenoids could downregulate the production of lipid mediator leukotrienes B_4_ (LTB_4_) and pro-inflammatory cytokine interleukin-4 (IL-4) in the antigen-stimulated RBL-2H3 mast cells. Further biological investigations revealed that dysivillosin A (**1**) could suppress the phosphorylation of Syk and PLCγ1 in IgE/FcɛRI/Syk signaling pathway, which resulted in the inhibition of degranulation and the downregulation of LTB_4_ and IL-4 production in mast cells.

## Introduction

Mast cells have long been considered as important effector cells in immunoglobulin E (IgE)-associated immune responses^1^. IgE binds to mast cells via its high-affinity receptor, FcεRI, and cross-linking of FcɛRI-bound IgE molecules by allergen triggers a cascade of signaling events which leads to mast cell degranulation and the release of allergic mediators characteristic of type I hypersensitivity reactions^2–4^. FcεRI aggregation initiates Src family kinases, and subsequently activates another key tyrosine kinase, Syk, which is most essential for the activation of various downstream signaling molecules, such as GTPase, Ras, PI3K, and PLCγ1^5^. Therefore, FcεRI-proximal kinase, Syk, has recently been proposed as an excellent therapeutic target for the treatment of allergic diseases^5^.

**Figure 1:**
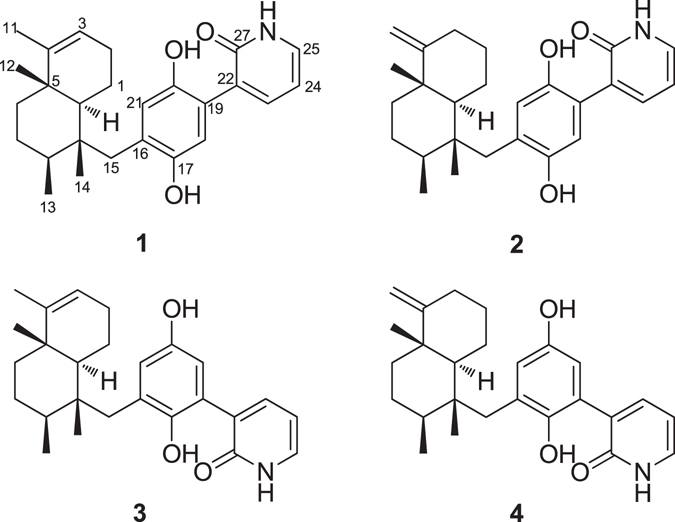
Structures of dysivillosins A–D (**1**–**4**).

Marine sponges are rich sources of diverse bioactive metabolites with unusual chemical scaffolds^6^. As part of our search for new anti-allergic compounds from the South China Sea marine sponges, several sponge specimens have been collected and screened for their inhibitory activity against β-hexosaminidase, a degranulation indicator of mast cells. Among them, the MeOH extract of *Dysidea villosa* showed the most potent inhibitory activity against β-hexosaminidase with a IC_50_ value of 5.3 μg/mL. Detailed bioassay-guided fractionation of the active organic extract led to the isolation of four unusual meroterpenoids, dysivillosins A–D (**1**–**4**), as depicted in Fig. 1. To the best of our knowledge, this is the first report of terpene-polyketide-pyridine hybrid metabolites from the nature. All the four compounds were evaluated for their anti-allergic activity, they could significantly inhibit β-hexosaminidase and the production of LTB4 and IL-4 in antigen-stimulated RBL-2H3 mast cells. Further investigations showed that anti-allergic activity of dysivillosin A was triggered by suppressing Syk/PLCγ1 signaling pathway.

## Results and Discussion

Dysivillosin A (**1**) was isolated as an amorphous powder ([*α*]_D_^26^ + 26.5) and gave a molecular formula of C_26_H_33_NO_3_ with 11 degrees of unsaturation, as determined by HRESIMS data. The IR spectrum indicated the presence of hydroxy (3185 cm^−1^), carbonyl (1713 and 1662 cm^−1^), and benzene (1591 and 1440 cm^−1^) groups^7, 8^, whereas the UV absorption maxima at 216, 255, and 303 nm were indicative of a 2-pyridone unit^9^. The ^1^H NMR spectrum of dysivillosin A (**1**) (Table 1) displayed the resonances for four methyls at *δ*_H_ 0.94, 1.03, 1.20, and 1.45, two singlet aromatic protons at *δ*_H_ 7.59 and 7.74, three mutually coupling olefinic protons at *δ*_H_ 7.17 (1 H, dd, *J* = 7.8, 4.8 Hz), 8.13 (1 H, dd, *J* = 7.8, 1.8 Hz), and 8.46 (1 H, dd, *J* = 4.8, 1.8 Hz), along with a phenolic hydroxy proton at *δ*_H_ 11.65. The ^13^C NMR and HSQC spectra revealed the presence of one conjugated carbonyl (*δ*_C_ 163.5), four aromatic quaternary carbons [*δ*_C_ 120.8, 128.4, 147.8 (oxygenated), and 153.9 (oxygenated)], two olefinic quaternary carbons (*δ*_C_ 117.0 and 143.6), two aromatic methines (*δ*_C_ 106.1 and 114.9), four olefinic methines (*δ*_C_ 118.4, 120.4, 129.1, and 145.4), two aliphatic quaternary carbons (*δ*_C_ 38.0 and 41.8), two aliphatic methines (*δ*_C_ 36.0 and 45.9), five methylenes (*δ*_C_ 19.5, 26.3, 27.4, 35.6, and 37.8), and four methyls (*δ*_C_ 17.3, 17.4, 17.6, and 19.6). These above NMR spectroscopic features were characteristic of a sesquiterpene hydroquinone containing a 2-pyridone unit^10^.

**Table 1:** ^1^H (600 MHz) and ^13^C (150 MHz) NMR Data for Dysivillosins A–D (**1**–**4**) in Pyr-*d*_5_.

Position	**1**		**2**		**3**		**4**	
	*δ*_C_	*δ*_H_ (*J* in Hz)	*δ*_C_	*δ*_H_ (*J* in Hz)	*δ*_C_	*δ*_H_ (*J* in Hz)	*δ*_C_	*δ*_H_ (*J* in Hz)
1α	19.5, CH_2_	2.33, m	22.8, CH_2_	2.35, m	19.1, CH_2_	2.39, m	22.5, CH_2_	2.41, m
1β		1.63, m		1.55, dd (12.0, 2.4)		1.57, m		1.53, dd (12.0, 3.6)
2a	26.3, CH_2_	2.38, m	28.0, CH_2_	1.93, m	26.1, CH_2_	2.23, m	27.9, CH_2_	1.92, m
2b		2.14, m		1.59, m		2.07, m		1.35, m
3α	120.4, CH	5.16, brs	32.6, CH_2_	2.36, m	120.4, CH	4.98, brs	32.5, CH_2_	2.26, td (15.0, 4.8)
3β				2.11, m				1.96, m
4	143.6, C		159.4, C		143.1, C		159.0, C	
5	38.0, C		39.9, C		37.8, C		39.8, C	
6α	35.6, CH_2_	0.89, td (13.2, 3.6)	36.4, CH_2_	1.29, dd (13.2, 3.6)	35.5, CH_2_	0.75, td (13.2, 3.6)	36.3, CH_2_	1.14, td (13.2, 3.6)
6β		1.49, dt (12.6, 3.0)		1.45, m		1.41, dt (12.6, 3.6)		1.35, m
7α	27.4, CH_2_	1.32, m	27.4, CH_2_	1.42, m	27.0, CH_2_	1.20, dd (13.2, 3.6)	27.0, CH_2_	1.41, m
7β		1.37, td (12.6, 1.2)		1.39, m		1.30, td (13.2, 3.0)		1.35, m
8	36.0, CH	1.81, m	36.3, CH	1.76, m	35.9, CH	1.69, m	36.2, CH	1.63, m
9	41.8, C		42.2, C		41.3, C		41.6, C	
10	45.9, CH	1.55, d (12.0)	48.2, CH	1.36, dd (12.0, 2.4)	45.9, CH	1.39, dd (12.6, 1.2)	48.3, CH	1.20, dd (12.0, 1.8)
11a	17.6, CH_3_	1.45, br s	102.6, CH_2_	4.46, s	17.5, CH	1.35, br s	102.6, CH_2_	4.32, s
11b				4.38, s				4.28, s
12	19.6, CH_3_	1.03, s	20.0, CH_3_	1.06, s	19.5, CH_3_	0.96, s	20.0, CH_3_	0.99, s
13	17.3, CH_3_	1.20, d (6.6)	17.4, CH_3_	1.20, d (6.6)	16.6, CH_3_	1.09, d (6.6)	16.8, CH_3_	1.10, d (6.6)
14	17.4, CH_3_	0.94, s	17.3, CH_3_	0.93, s	17.3, CH_3_	0.87, s	17.2, CH_3_	0.88, s
15a	37.8, CH_2_	3.13, s	37.7, CH_2_	3.09, d (14.4)	37.2, CH_2_	3.20, d (14.4)	37.3, CH_2_	3.12, d (14.4)
15b				3.04, d (14.4)		2.93, d (14.4)		2.88, d (14.4)
16	128.4, C		128.2, C		124.1, C		123.9, C	
17	153.9, C		153.8, C		147.9, C		149.7, C	
18	106.1, CH	7.74, s	106.1, CH	7.69, s	122.3, C		122.4, C	
19	120.8, C		120.8, C		105.0, CH	7.68, d (2.4)	105.1, CH	7.63, d (3.0)
20	147.8, C		147.8, C		154.3, C		154.2, C	
21	114.9, CH	7.59, s	114.6, CH	7.53, s	120.0, CH	7.26, d (2.4)	119.7, CH	7.20, d (3.0)
22	117.0, C		117.0, C		117.3, C		117.2, C	
23	129.1, CH	8.13, dd (7.8, 1.8)	129.1, CH	8.10, dd (7.8, 1.8)	129.5, CH	8.21, dd (7.2, 1.8)	129.4, CH	8.18, dd (7.8, 1.8)
24	118.4, CH	7.17, dd (7.8, 4.8)	118.4, CH	7.16, dd (7.8, 4.8)	118.6, CH	7.21, dd (7.2, 4.8)	118.5, CH	7.16, dd (7.8, 4.8)
25	145.4, CH	8.46, dd (4.8, 1.8)	145.4, CH	8.45, dd (4.8, 1.8)	145.8, CH	8.52, dd (4.8, 1.8)	145.8, CH	8.50, dd (4.8, 1.8)
27	163.5, C		163.5, C		163.3, C		163.3, C	
17-OH		11.65, br s		11.63, br s		11.67, br s		11.63, br s

**Figure 2:**
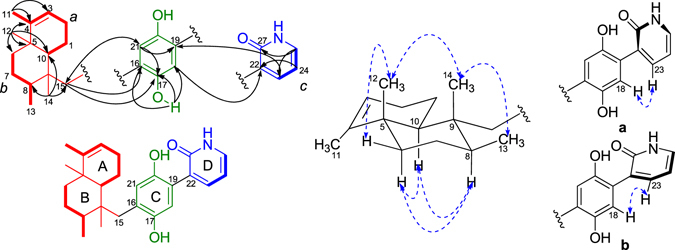
Key COSY, HMBC, and NOESY correlations of **1**.

The COSY correlations of H_2_-1/H-10, H_2_-1/H_2_-2, H_2_-2/H-3, H_2_-6/H_2_-7, H_2_-7/H-8, H-8/H_3_-13, H-23/H-24, and H-24/H-25 coupled with HSQC spectrum revealed the presence of three isolated spin systems: (*a*) C-10–C-1–C-2–C-3, (*b*) C-6–C-7–C-8–C-13, and (*c*) C-23 –C-24–C-25, as shown in Fig. 2. The elongation from C-3 to C-11 was evident from the allylic COSY correlation of H-3/H_3_-11. The connections of the three spin systems with the other carbons were mainly determined by analysis of the HMBC spectrum. The long-ranged HMBC correlations of H_3_-11/C-3, C-4, and C-5 and H_3_-12/C-4, C-5, C-6, and C-10 connected the two partial structures *a* and *b via* C-5 in rings A and B and also allowed the assignment of the two methyls at C-4 and C-5, respectively. Moreover, the HMBC correlations of H_3_-13/C-7 and C-9 and H_3_-14/C-8, C-9, C-10, and C-15 established a tetramethyl decalin sesquiterpene moiety with a methylene CH_2_-15 tethered at C-9. This assignment was supported by the HMBC correlations of H-10/C-4, C-5, C-6, C-9, C-14 and C-15 and H_2_-15/C-8, C-9, C-10, and C-14. In the down field, the aromatic proton H-18 showed HMBC correlations with C-16 and the oxygenated aromatic carbon C-20, meanwhile H-21 showed HMBC correlations with C-19 and oxygenated aromatic carbon C-17, which indicated the presence of a 1,4-hydroquinone unit (ring C) in **1**. The HMBC correlations from the phenolic hydroxy proton at *δ*_H_ 11.65 to C-16, C-17, and C-18 assigned the phenolic hydroxy group at C-17. Additionally, HMBC correlations of H_2_-15/C-16, C-17, and C-21 and H-21/C-15 suggested that the sesquiterpene fragment was linked to hydroquinone unit through the carbon bond C-15–C-16 (Fig. 2)^10^. What’s more, HMBC correlations of H-24/C-22 and H-23/C-27, and H-25/C-27 coupled with the remaining conjugated amide carbonyl C-27 (*δ*_C_ 163.5) indicated the presence a 2-pyridone substructure (ring D). Finally, HMBC correlations of H-18/C-22 and H-23/C-19 determined that the 2-pyridone unit was tethered at C-19 in the hydroquinone, supported by the NOESY correlation of H-18/H-23.

Once the planar structure of **1** was established, its relative configuration was addressed by NOESY experiments, as shown in Fig. 2. The large coupling constant value of H-10 (*J* = 12.0 Hz) and the chemical shift of CH_3_-12 (*δ*_C_ 19.6/*δ*_H_ 1.03) suggested the *trans*-fusion of the bicyclic system (rings A/B)^11, 12^, which was confirmed by the NOESY correlations of H_3_-12/H-6β and H-10/H-6α. The NOESY correlations of H-8/H-10 and H-6α/H-10 indicated the α-orientation of the three protons, meanwhile the correlations of H_3_-12/H_3_-14 and H_3_-13/H_3_-14 assigned the three methyl groups as β-orientation. Thus, the relative configuration of **1** was determined as 5S*, 8S*, 9R*, 10S*, as depicted in Fig. 2. Additionally, the aromatic proton H-18 in the hydroquinone unit showed NOESY correlations with H-23 in the 2-pyridone moiety, which suggested the presence of two possible configurations (**a** and **b**) of the axial chiral center C-19–C-22 (Fig. 2).

**Figure 3:**
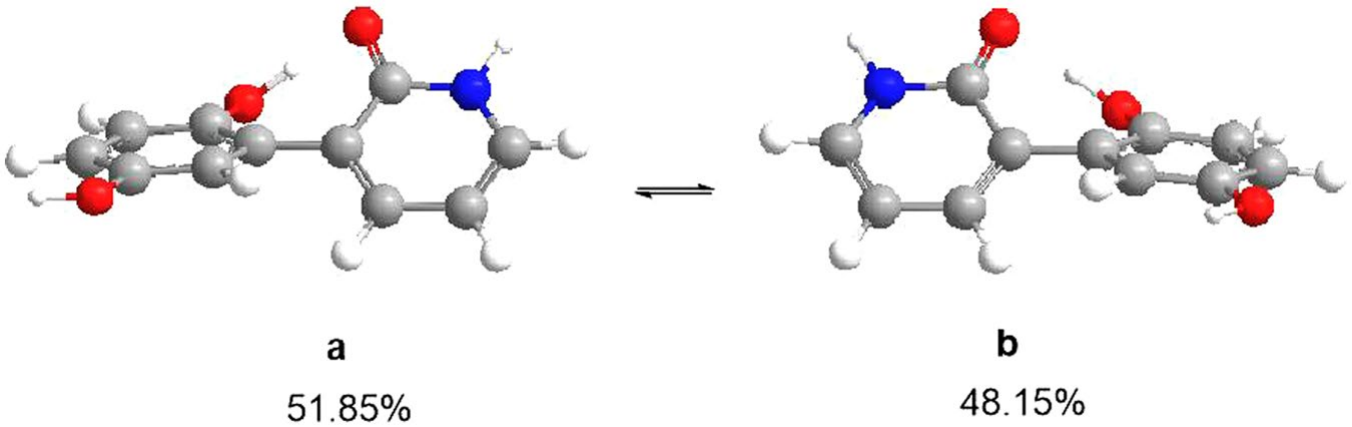
The two conformers ((**a**) and (**b**)) of hydroquinone-2-pyridone (ΔE = 0–5 kcal/mol). The Boltzman population ratios of **a** and **b** are respective of 51.85% and 48.15% at the temperature of 298.15 K.

**Figure 4:**
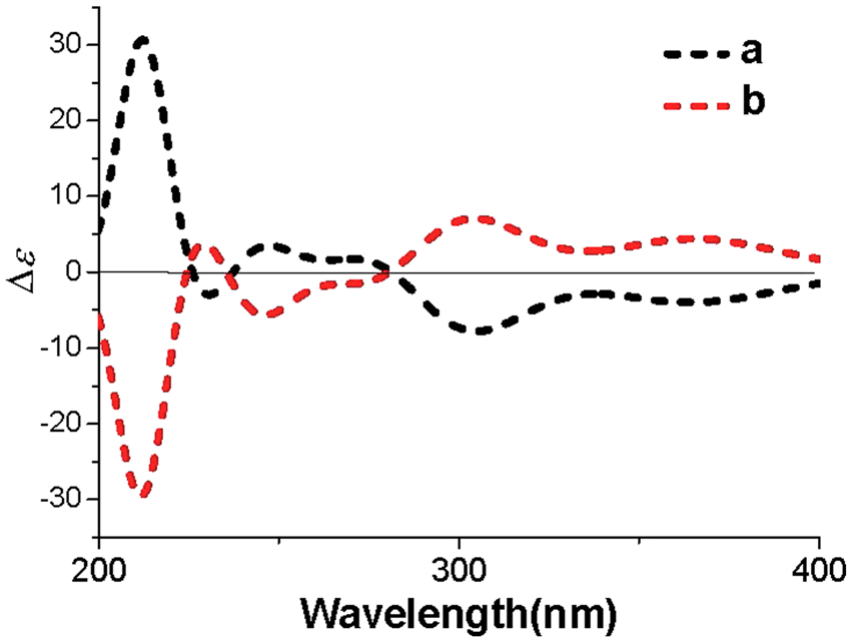
The calculated ECD spectra of the two conformers (**a** and **b**) for hydroquinone-2-pyridone.

**Figure 5:**
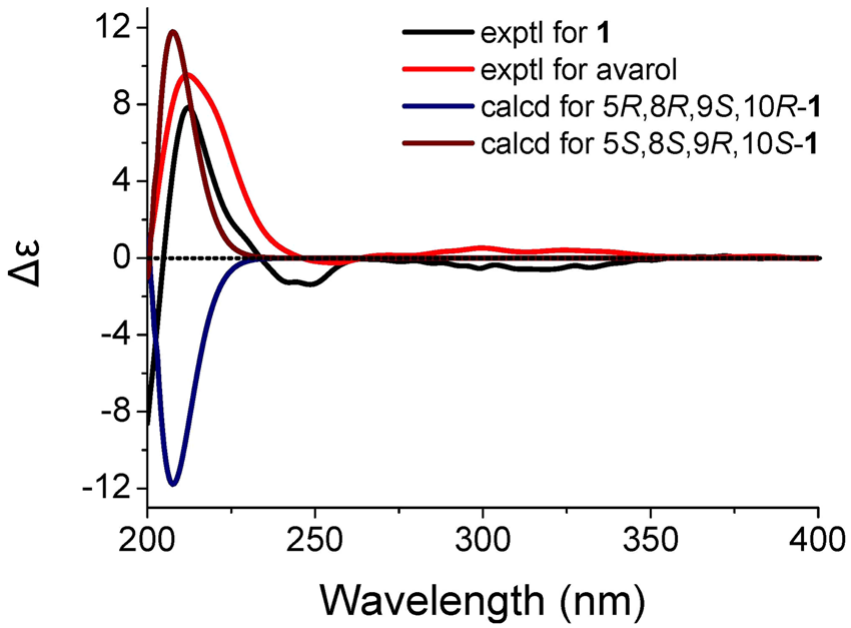
Comparison of the experimental ECD spectra of **1** and avarol to the calculated ECD spectra of **1** and its enantiomer.

Despite of the best efforts, the crystallization of **1** was finally unsuccessful. In order to determine the whole absolute configuration of **1**, quantum chemical ECD calculation methods were employed to determine the absolute configuration of **1** ^13^. Firstly, a hydroquinone-2-pyridone model structure was used to investigate the configuration of the axial chirality in the structure of **1** by HF/6-31G(d) method in Gaussian09^14^. Only two equilibrium conformers (**a** and **b**) were found using molecular mechanics (ΔE = 0–5 kcal/mol), as shown in Fig. 3, which is identical with the deduction form the NOESY experiment. The Boltzman population ratios of the two conformers are 51.85% (**a**) and 48.15% (**b**) at the temperature of 298.15 K, and their dihedral angels are 37.5° and 37.6°, respectively. It was interesting to find that the Cotton effects (CEs) in the calculated ECD spectrum of **a** were opposite to those of **b**, as shown in Fig. 4. The majority of axial chiral compounds have a rotational barrier sufficiently high to prevent racemization of the atropisomers at room temperature, and the separation of stereoisomers at room temperature requires energy barriers of at least 22 kcal/mol^15^. However, conformer **a** could easily convert to **b** with the energy barriers of less than 5 kcal/mol, which suggested that the axial chiral center in compound **1** could isomerize from *M* to *P* atropisomer at room temperature. Therefore, the whole absolute configuration of **1** was mainly determined by the configurations of four chiral centers (C-5, C-8, C-9 and C-10) in the sesquiterpene moiety. Subsequently, two optimized stereoisomers, (5*R*, 8*R*, 9*S*, 10*R*)-**1** and (5*S*, 8*S*, 9*R*, 10*S*)-**1**, were built based on the relative configuration of **1**, their ECD spectra were calculated at the level of PBE0/6-311 ++ G (2d, 2p) with MeOH as the solvent^16, 17^. The positive CE at 210 nm in the calculated spectrum of the 5*S*, 8*S*, 9*R*, 10*S* enantiomer matched well with the CE observed in the experimental ECD spectrum of **1** (Fig. 5), which determined the absolute configuration of **1** as 5*S*, 8*S*, 9*R*, 10*S*, identical with that of avarol.

**Figure 6:**
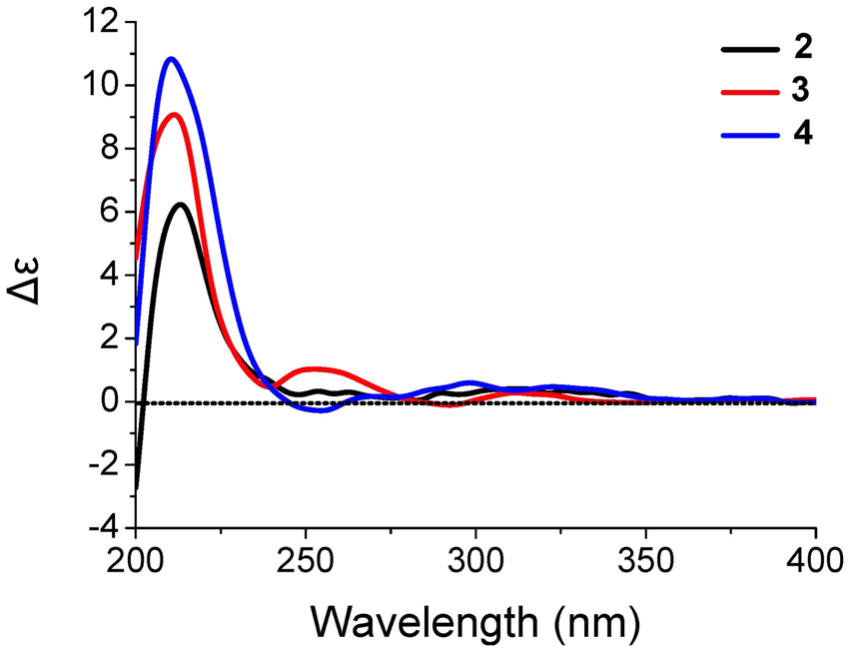
Experimental ECD spectra of **2–4**.

Dysivillosin B (**2**) was assigned the same molecular formula C_26_H_33_NO_3_ as that of **1** by HRESIMS data. The ^1^H and ^13^C NMR data of **2** corresponded closely to those of **1** (Table 1), except for three subunits, a methylene CH_2_-3 (*δ*_C_ 32.6/*δ*_H_ 2.36, 2.11), an olefinic quaternary carbon C-4 (*δ*_C_ 159.4), and an exomethylene CH_2_-11 (*δ*_C_ 102.6/*δ*_H_ 4.46, 4.38), which implied that the avarol sesquiterenoid fragment in **1** was replaced by an neoavarol fragment in **2** ^18^. This assignment was further supported by detailed analyses of COSY and HMBC correlations (Figure S1). Similar NOESY correlations and CE in the ECD spectrum (Fig. 6) showed that dysivillosin B shared the same relative and absolute configurations with those of **1**.

Dysivillosin C (**3**) was also obtained as an isomer of **1**, based on the HRESIMS data. Comprehensive analysis of the ^1^H and ^13^C NMR data of **3** (Table 1) showed that the only difference between **1** and **3** was the position of 2-pyridone, which tethered at C-18 in **3** rather than at C-19 in **1**. This assignment was supported by HMBC correlations of H-19/C-22 and H-23/C-18 as well as the NOESY correlation of H-19/H-23 (Figure S1). Subsequent analyses of the NOESY and ECD spectra of **3** disclosed that its relative and absolute configurations were both consistent with those of **1** (Fig. 6).

The HRESIMS data indicated that dysivillosin D (**4**) was an isomer of **1**–**3**. Detailed comparison of the NMR data of **4** with those of **2** indicated that the main difference was the position of 2-pyridone in the hydroquinone unit (Table 1). Key HMBC correlations of H-19/C-22 and H-23/C-18 positioned the 2-pyridone at C-18 (Figure S1). Identical NOESY correlations and similar CE in the ECD spectrum showed that dysivillosin D shared the same relative and absolute configurations with those of **1**–**3**, as shown in Fig. 6.

**Figure 7:**
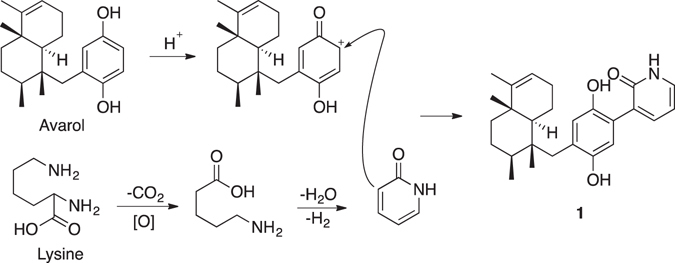
Plausible Biogenetic Pathway of **1**.

There are a large number of naturally occurring derivatives of avarol/avarone and meroterpenoids where the hydroquinone/quinone is substituted by amines and amino acids^19–23^. Recently, Anderson *et al*. reported the structure of avinosol, a meroterpenoid-nucleoside conjugate^24^. However, dysivillosins A–D (**1**–**4**) are the first example of terpene-polyketide-pyridine hybrid compounds from the nature. The proposed biosynthesis of **1**–**4** could be involved with the biosynthetic pathways of three precursors: terpenoid, acetate, and pyridine. The four compounds could be biogenetically related to two meroterpenoids, avarol and neoavarol, which were both isolated from marine sponges belonging to the *Dysidea* genus^10, 18, 25^. As shown in Fig. 7, 2-pyridone might be produced by *L*-lysine via successive decarboxylation, amidation, and dehydrogenation reactions^26^. Subsequent nucleophilic attack from the 2-pyridone to the hydroquinone unit in avarol could finally result in the formation of dysivillosin A (**1**)^27^.

**Figure 8:**
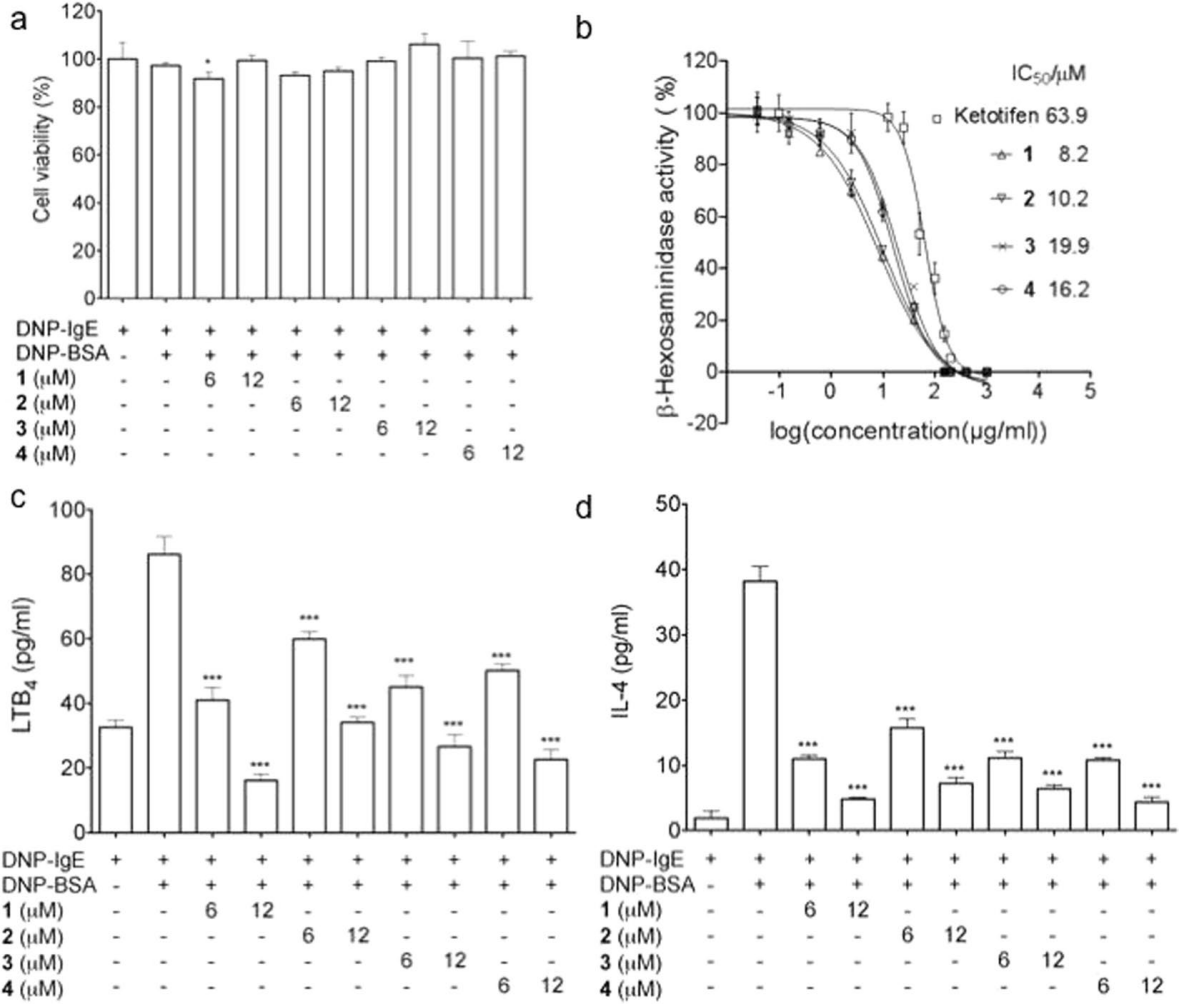
(**a**) Cytotoxic effects of **1**–**4** on RBL-2H3 cells. (**b**) Inhibitory effects of **1**–**4** on the release of β-hexosaminidase in DNP-IgE-activated RBL-2H3 cells. (**c**) Inhibitory activity on lipid mediator LTB_4_ secretion of **1**–**4** in RBL-2H3 cells. (**d**) Inhibition of IL-4 production of **1**–**4** in RBL-2H3 cells. Data are presented as the mean ± SD values of triplicate determinations. *P < 0.05, **P < 0.01, ***P < 0.001 versus DNP-BSA-treated group (n = 3). Data are analyzed by one-way ANOVA followed by Tukey’s Multiple Comparison Post-Test (GraphPad Prism 5.0).

The four compounds (**1**–**4**) from *D. villosa* were screened for their anti-allergic activity on rat basophilic leukemia (RBL-2H3) cells. All of them showed no cytotoxicity on RBL-2H3 cells at the concentrations of 6 and 12 μM, as depicted in Fig. 8a. Subsequently, the inhibitory activity of **1**–**4** on the degranulation in DNP-IgE-activated RBL-2H3 mast cells was examined, using ketotifen as a positive control^28^. Dysivillosins A–D were found to potently inhibit the release of β-hexosaminidase, a marker of degranulation, in a dose-dependent manner with IC_50_ values of, 8.2, 10.2, 19.9, and 16.2, respectively (Fig. 8b). As the main allergic mediators released by mast cells, lipid mediator LTB_4_ and pro-inflammatory cytokine IL-4 are involved in acute allergic responses^29, 30^, and the inhibitory activity of **1**–**4** on LTB_4_ and IL-4 was investigated as well. As depicted in Fig. 8c and d, all the four compounds could dose dependently downregulate the production of LTB_4_ and IL-4 in the antigen-stimulated RBL-2H3 mast cells at the concentrations of 6 and 12 μM. Among the four compounds, dysivillosin A (**1**) showed the most potent anti-allergic activity, and thus was selected for further inhibitory activity evaluation on the antigen specific IgE/FcɛRI/Syk signaling pathway, because both of the degranulation and subsequent production of LTB_4_ and IL-4 in mast cells are triggered by cross-linking of FcεRI-bound IgE molecules^31, 32^.

**Figure 9:**
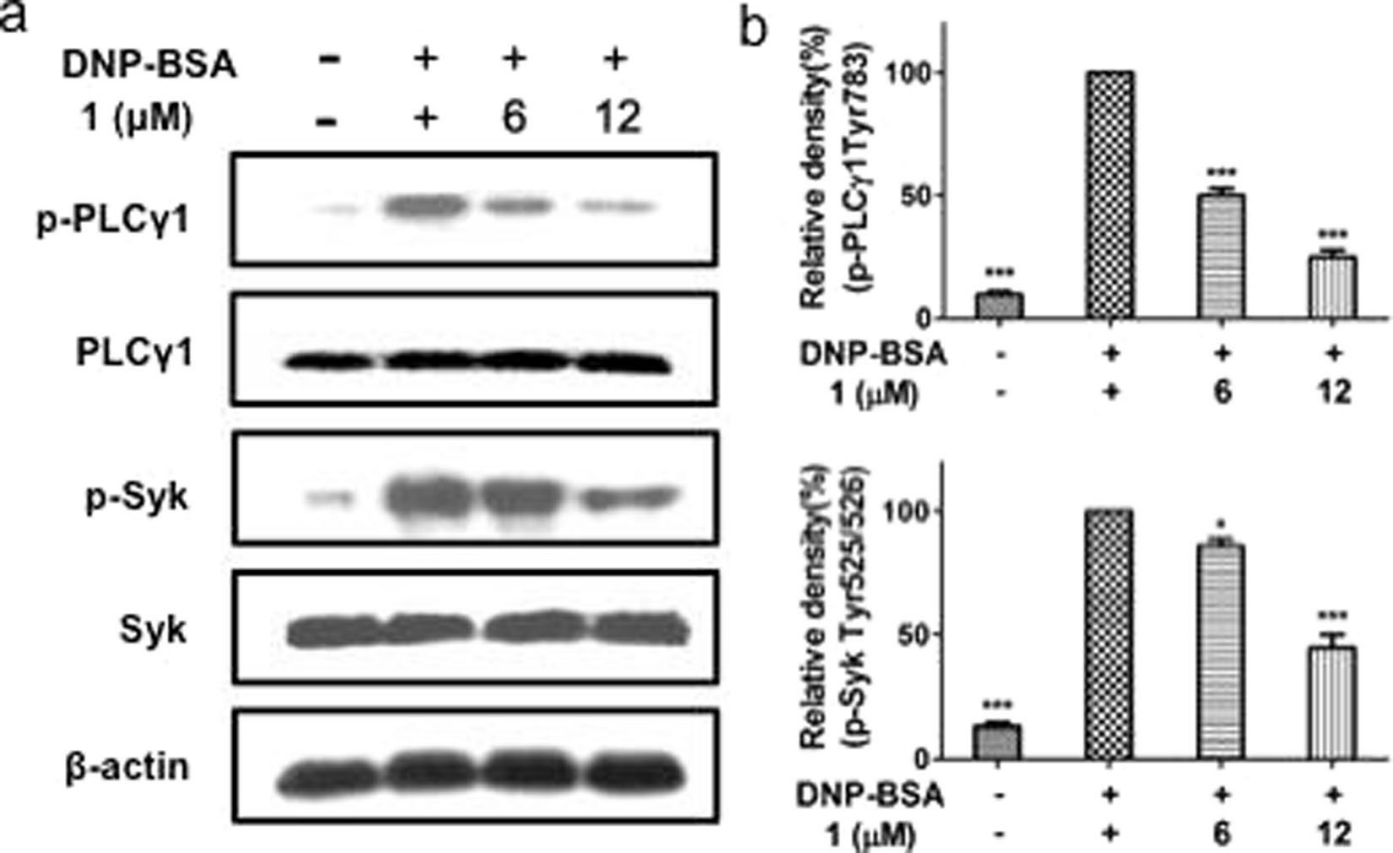
Comparison of the dysivillosin A (**1**) inhibited the activation of signaling molecules in antigen-stimulated RBL-2H3 cells. IgE-sensitized RBL-2H3 cells (2 × 10^6^ cells/well) were stimulated with 1 μg/ml DNP-BSA for 15 min in the presence or absence of **1**. The levels of p-Syk, Syk, p-PLCγ1 and PLCγ1 were measured by immunoblotting analysis. (**a**) The blots are representative of three independent experiments. (**b**) The data are presented as means ± SD. *P < 0.05, ***P < 0.001 versus DNP-BSA-treated group (n = 3). Data are analyzed by one-way ANOVA followed by Tukey’s Multiple Comparison Post-Test (GraphPad Prism 5.0).

Interestingly, we found that dysivillosin A (**1**) could significantly inhibit the activation of Syk as well as its downstream signaling molecule, PLCγ1, as shown in Fig. 9a and b. this finding indicated that dysivillosin A could inhibit mast cell activation by suppressing the phosphorylation of Syk and PLCγ1, which subsequently inhibited the degranulation and downregulated the production of LTB_4_ and IL-4. These results highlight the potential of dysivillosin A (**1**) as a new chemotherapeutic scaffold targeting Syk-associated allergy.

## Methods

### General experimental procedures

Optical rotations were measured with an Autopol I-30575 (*l* = 10 cm) and UV spectra on a Hitachi U-3010 spectrophotometer; IR spectra (KBr) were obtained with a Jasco FTIR-400 spectrometer, whereas ECD spectra were obtained on a Jasco J-810 spectropolarimeter. NMR spectra were obtained with a Bruker Avance NMR spectrometer at 600 MHz for ^1^H and 150 Hz for ^13^C using Pyr-*d*_5_ as solvent. HRESIMS data were obtained on an Agilent 6210 LC/MSD TOF mass spectrometer.

### Collection, extraction, and isolation

Samples of *Dysidea villosa* were collected from the coast of Yongxing Island in South China sea on May 7^th^, 2011. The voucher sample of *D. villosa* (XD110507A) was maintained at the Research Center for Marine Drugs, State Key Laboratory of Oncogenes and Related Genes, Department of Pharmacy, Ren Ji Hospital, School of Medicine, Shanghai Jiao Tong University. The animals (520 g, wet weight) was extracted by MeOH repeatedly to give 9.2 g extract, which showed potent inhibitory activity against β-hexosaminidase (IC_50_ = 5.3 μg/mL). The bioactive extract was dissolved in 500 mL water, and partitioned with the same volume of CH_2_Cl_2_ four times to yield 8.7 g CH_2_Cl_2_-soluble fraction, which was subjected to a silica gel chromatography column eluted with gradient *n*-hexane and EtOAc, yielding 14 subfractions (DA-DN). Fraction DK (1.2 g) was passed through open ODS chromatography column eluted with gradient aqueous MeOH, size-exclusion chromatography Sephadex LH-20 eluted with CH_2_Cl_2_/MeOH (1:1), and then purified by reversed-phased HPLC (YMC C_18_ column, 10 × 250 mm, 2 mL/min, 297 nm) with a elution of 80% CH_3_CN, to give dysivillosin A (**1**, 7.0 mg, *t*_R_ 79.1 min), dysivillosin B (**2**, 2.3 mg, *t*_R_ 74.0 min), dysivillosin C (**3**, 6.4 mg, *t*_R_ 62.3 min), and dysivillosin D (**4**, 2.7 mg, *t*_R_ 58.1 min).

**Dysivillosin A (1):** colorless solid; [*α*]_D_^26^ + 26.5 (*c* 0.1, MeOH); UV (MeOH) *λ*_max_ (log*ε*) 216 (4.12), 255 (3.90), 303 (4.08), 331 (3.65) nm; CD (MeOH) *λ* (Δ*ε*) 212 (+8.51) nm; IR (KBr) *ν*_max_ 3185, 2960, 2922, 2854, 1713, 1662, 1591, 1444, 1261, 1166, 1094, 1021, 799 cm^−1^; ^1^H and ^13^C NMR data see Table 1; HRESIMS *m*/*z* 388.2274 [M - H_2_O - H]^−^ (calcd for C_26_H_30_NO_2_, 388.2277).

**Dysivillosin B (2):** colorless solid; [*α*]_D_^26^ + 6.7 (*c* 0.1, MeOH); UV (MeOH) *λ*_max_ (log*ε*) 202 (4.01), 248 (3.54), 301 (3.74), 326 (3.34) nm; CD (MeOH) *λ* (Δ*ε*) 209 (+5.56) nm; IR (KBr) *ν*_max_ 2958, 2924, 2855, 1735, 1665, 1634, 1592, 1444, 1378, 1260, 1162, 1093, 1023, 800 cm^−1^; ^1^H and ^13^C NMR data see Table 1; HRESIMS *m*/*z* 388.2275 [M - H_2_O - H]^−^ (calcd for C_26_H_30_NO_2_, 388.2277).

**Dysivillosin C (3):** colorless solid; [*α*]_D_^26^ + 4.2 (*c* 0.3, MeOH); UV (MeOH) *λ*_max_ (log*ε*) 216 (4.11), 254 (3.70), 297 (3.91), 326 (3.33) nm; CD (MeOH) *λ* (Δ*ε*) 210 (+9.21) nm; IR (KBr) *ν*_max_ 3194, 2957, 2925, 2856, 1713, 1664, 1620, 1594, 1461, 1403, 1379, 1253, 1171 cm^−1^; ^1^H and ^13^C NMR data see Table 1; HRESIMS *m*/*z* 388.2279 [M - H_2_O - H]^−^ (calcd for C_26_H_30_NO_2_, 388.2277).

**Dysivillosin D (4):** colorless solid; [*α*]_D_^26^ + 8.5 (*c* 0.1, MeOH); UV (MeOH) *λ*_max_ (log*ε*) 205 (4.08), 244 (3.60), 297 (3.85), 326 (2.82) nm; CD (MeOH) *λ* (Δ*ε*) 207 (+11.03) nm; IR (KBr) *ν*_max_ 3203, 2959, 2925, 2855, 1737, 1666, 1616, 1595, 1495, 1454, 1261, 1174, 1093, 1022, 800 cm^−1^; ^1^H and ^13^C NMR data see Table 1; HRESIMS *m/z* 388.2279 [M - H_2_O - H]^−^ (calcd for C_26_H_30_NO_2_, 388.2277).

### Energy Minimization and ECD Calculations

The molecule of **1** was converted into SMILES code before its initial 3D structure was generated with CORINA version 3.4. The conformer database of **1** was generated using CONFLEX version 7.0 with an energy window for acceptable conformers (ewindow) of 5 kcal mol^**−**1^ above the ground state using the modified version of the MMFF94 force field. Each conformer was optimized with the HF/6-31G(d) method in Gaussian09. Further optimization at the B3P86/6-31G(d) level led to the observed dihedral angles. The optimized conformers were taken for the ECD calculations, which were performed with Gaussian09 (B3P86/6-311++G(2d,p))^13^. The solvent effects were taken into account by the conductor-like polarizable continuum model (MeOH as the solvent)^14, 16^. To gain ECD curves from the output files that were suited for comparison with the experimental data, the length representation of the oscillator and rotatory strengths was always selected and the spectra were overlaid with Gaussian profiles that were in turn summed to give, after Boltzmann weighting of the individual spectra, a calculated ECD spectrum.

### Cell Culture

Rat basophilic leukemia (RBL-2H3) cells from the cell bank of Shanghai Science Academy were cultured in Dulbecco’s modified Eagle’s medium (DMEM), which contains 10% (v/v) FBS, 100 U/mL penicillin, and 100 μg/mL streptomycin, at 37 °C in a humidified atmosphere of 5% CO_2_.

### Cell Viability

MTT (3-(4,5-dimethyl-2-thiazolyl)-2,5-diphenyl-2-H-tetrazolium bromide) assay was conducted to examine cell viability. RBL-2H3 cells (5 × 10^5^ cells/well, 100 μL/well) were plated into a 96-well plate. After 24 h of incubation, cells were incubated with compounds **1**–**4** for 3 hours and the medium was replaced by MTT solution (250 μg/ml) and incubated at 37 °C for 4 h. The medium was then carefully discarded and the formazan was resuspended in 150 μL of dimethyl sulfoxide (DMSO). The absorbance was measured at 490 nm using a microplate reader. Values measured from untreated cells were considered to represent 100% viability.

### β-hexosaminidase Release Activity

RBL-2H3 cells were seeded in a 24-well plate (5 × 10^5^ cells/well), and were sensitized with dinitrophenyl (DNP)-specific IgE (DNP-IgE) (1 μg/mL) at 37 °C for overnight. DNP-IgE-sensitized cells were preincubated with sponge extracts or dysivillosins A–D (**1**–**4**) for 30 min, and then stimulated with DNP-BSA for 1.5 h. To measure the activity of β-hexosaminidase released from the cells, the cultured media were centrifuged (17,000 g, 10 min) at 4 °C. The supernatant (50 μL) was mixed with 50 μL of 0.1 M sodium citrate buffer (pH 4.5) containing 10 mM 4-nitrophenyl N-acetyl-β-D-glucosaminide in a 96-well plate, and then was incubated at 37 °C for 90 min. The absorbance was measured at 405 nm after terminating the reaction by 0.2 M glycine (pH 10.0).

### Determination of IL-4 and LTB_4_

To measure Interleukin-4 (IL-4) and Leukotriene B_4_ (LTB_4_) level in the cultured media, all cultured media were centrifuged at 4 °C, and the test compounds were stored at −80 °C until assay. IL-4 and LTB4 were quantified using an ELISA kit according to the manufacturer’s instructions.

### Immunoblot Analysis

After cell collection and extraction with the protein extraction solution, the protein samples were subjected to 10% SDS–polyacrylamide gel electrophoresis (PAGE). The protein spots were electrotransferred to a PVDF membrane, and then the membrane was incubated with blocking buffer (Tris-buffered saline (TBS) containing 0.05% Tween-20 and 5% w/v nonfat dry milk) for 1 h and was washed by TBST (TBS containing 0.05% Tween-20) three times, each time for 5 min, followed by a diluted solution of anti-p-syk, anti-syk, anti-p-PLCγ1, and anti-PLCγ1 antibodies (Cell Signaling Technology) overnight at 4 °C. In addition, the blots probed with a 1:1000 diluted solution of anti-β-actin antibody (Abcam) were used as internal control to keep each well loading with the same amount of protein. The membrane was washed by TBST for three times, each time for 10 min, shaken in a solution of horseradish peroxidase (HRP)-linked anti-rabbit or anti-mouse IgG secondary antibody, and washed by TBST for another three times, each time for 10 min. The expressions of proteins were detected by enhanced chemiluminescent (ECL) reagent (Thermo Scientific), followed by exposure of the membranes to photographic film (Fujifilm Corporation). The intensity of blots were analyzed and quantitated with ImageJ software.

### Statistical Analysis

The data were analyzed using a one-way ANOVA followed by Dunnett’s Multiple Comparison Test with GraphPad Prism software (GraphPad Prism version 5.01 for Windows, San Diego, CA, USA). The values are expressed as the means ± SD. The differences with p < 0.05 were considered significant.

## Electronic supplementary material

Supplementary Information
