# Supplementary material for: Dysivillosins A–D, Unusual Anti-allergic Meroterpenoids from the Marine Sponge Dysidea villosa
Source: Sci Rep. 2017 Aug 21;7:8947. doi: 10.1038/s41598-017-04021-z (PMC5567184; doi:10.1038/s41598-017-04021-z)
Supplement: Supplementary file 1 — Supplementary Information [file 41598_2017_4021_MOESM1_ESM.doc]

**Supporting Information**

Dysivillosins A−D, Unusual Anti-allergic Meroterpenoids from the Marine Sponge *Dysidea villosa*

Wei-Hua Jiao^1,+^, Bao-Hui Cheng^2,+^, Guo-Hua Shi^1^, Guo-Dong Chen^3^, Bin-Bin Gu^1^, Yong-Jun Zhou^1^, Li-Li Hong^1^, Fan Yang^1^, Zhi-Qiang Liu^2^, Shu-Qi Qiu^2^, Zhi-Gang Liu^2,4^, Ping-Chang Yang*^,2,4^, and Hou-Wen Lin*^,1^

^1^Research Center for Marine Drugs, State Key Laboratory of Oncogenes and Related Genes, Department of Pharmacy, Ren Ji Hospital, School of Medicine, Shanghai Jiao Tong University, Shanghai 200127, China

^2^Shenzhen Key Laboratory of ENT, Longgang ENT hospital & Institute of ENT, Shenzhen 518172, China

^3^Institute of Traditional Chinese Medicine & Natural Products, College of Pharmacy, Jinan University, Guangzhou 510632, China

^4^Shenzhen Key Laboratory of Allergy & Immunology, Shenzhen University School of Medicine, Shenzhen 518060, China

*****Corresponding authors: [yangp@cmaster.ca](mailto:yangp@cmaster.ca) (P.C. Yang) and [franklin67@126.com](mailto:franklin67@126.com) (H.W. Lin)

^+^These authors contributed equally to this work.

**Contents**

**Table S1.** ^1^H and ^13^C NMR Spectroscopic Data for Dysivillosin A (**1**) in Pyr-*d*_5_

**Table S2.** ^1^H and ^13^C NMR Spectroscopic Data for Dysivillosin B (**2**) in Pyr-*d*_5_

**Table S3.** ^1^H and ^13^C NMR Spectroscopic Data for Dysivillosin C (**3**) in Pyr-*d*_5_

**Table S4.** ^1^H and ^13^C NMR Spectroscopic Data for Dysivillosin D (**4**) in Pyr-*d*_5_

**Energy Minimization and ECD Calculations**

**Figure S1.** Key 2D NMR correlations of Dysivillosins B−D (2−**4**).

**Figure S2.** ^1^H NMR Spectrum of Dysivillosin A (**1**) in Pyr-*d*_5_.

**Figure S3.** ^13^C NMR Spectrum of Dysivillosin A (**1**) in Pyr-*d*_5_.

**Figure S4.** DEPT135 Spectrum of Dysivillosin A (**1**) in Pyr-*d*_5_.

**Figure S5.** ^1^H-^1^H COSY Spectrum of Dysivillosin A (**1**) in Pyr-*d*_5_.

**Figure S6.** HSQC Spectrum of Dysivillosin A (**1**) in Pyr-*d*_5_.

**Figure S7.** HMBC Spectrum of Dysivillosin A (**1**) in Pyr-*d*_5_.

**Figure S8.** NOESY Spectrum of Dysivillosin A (**1**) in Pyr-*d*_5_.

**Figure S9.** HRESIMS of Dysivillosin A (**1**).

**Figure S10.** CD Spectrum of Dysivillosin A (**1**) in MeOH.

**Figure S11.** UV Spectrum of Dysivillosin A (**1**) in MeOH.

**Figure S12.** IR Spectrum of Dysivillosin A (**1**).

**Figure S13.** ^1^H NMR Spectrum of Dysivillosin B (**2**) in Pyr-*d*_5_.

**Figure S14.** ^13^C NMR Spectrum of Dysivillosin B (**2**) in Pyr-*d*_5_.

**Figure S15.** DEPT135 Spectrum of Dysivillosin B (**2**) in Pyr-*d*_5_.

**Figure S16.** ^1^H-^1^H COSY Spectrum of Dysivillosin B (**2**) in Pyr-*d*_5_.

**Figure S17.** HSQC Spectrum of Dysivillosin B (**2**) in Pyr-*d*_5_.

**Figure S18.** HMBC Spectrum of Dysivillosin B (**2**) in Pyr-*d*_5_.

**Figure S19.** NOESY Spectrum of Dysivillosin B (**2**) in Pyr-*d*_5_.

**Figure S20.** HRESIMS of Dysivillosin B (**2**).

**Figure S21.** CD Spectrum of Dysivillosin B (**2**) in MeOH.

**Figure S22.** UV Spectrum of Dysivillosin B (**2**) in MeOH.

**Figure S23.** IR Spectrum of Dysivillosin B (**2**).

**Figure S24.** ^1^H NMR Spectrum of Dysivillosin C (**3**) in Pyr-*d*_5_.

**Figure S25.** ^13^C NMR Spectrum of Dysivillosin C (**3**) in Pyr-*d*_5_.

**Figure S26.** DEPT135 Spectrum of Dysivillosin C (**3**) in Pyr-*d*_5_.

**Figure S27.** ^1^H-^1^H COSY Spectrum of Dysivillosin C (**3**) in Pyr-*d*_5_.

**Figure S28.** HSQC Spectrum of Dysivillosin C (**3**) in Pyr-*d*_5_.

**Figure S29.** HMBC Spectrum of Dysivillosin C (**3**) in Pyr-*d*_5_.

**Figure S30.** NOESY Spectrum of Dysivillosin C (**3**) in Pyr-*d*_5_.

**Figure S31.** HRESIMS of Dysivillosin C (**3**).

**Figure S32.** CD Spectrum of Dysivillosin C (**3**) in MeOH.

**Figure S33.** UV Spectrum of Dysivillosin C (**3**) in MeOH.

**Figure S34.** IR Spectrum of Dysivillosin C (**3**).

**Figure S35.** ^1^H NMR Spectrum of Dysivillosin D (**4**) in Pyr-*d*_5_.

**Figure S36.** ^13^C NMR Spectrum of Dysivillosin D (**4**) in Pyr-*d*_5_.

**Figure S37.** DEPT135 Spectrum of Dysivillosin D (**4**) in Pyr-*d*_5_.

**Figure S38.** ^1^H-^1^H COSY Spectrum of Dysivillosin D (**4**) in Pyr-*d*_5_.

**Figure S39.** HSQC Spectrum of Dysivillosin D (**4**) in Pyr-*d*_5_.

**Figure S40.** HMBC Spectrum of Dysivillosin D (**4**) in Pyr-*d*_5_.

**Figure S41.** NOESY Spectrum of Dysivillosin D (**4**) in Pyr-*d*_5_.

**Figure S42.** HRESIMS of Dysivillosin D (**4**).

**Figure S43.** CD Spectrum of Dysivillosin D (**4**) in MeOH.

**Figure S44.** UV Spectrum of Dysivillosin D (**4**) in MeOH.

**Figure S45.** IR Spectrum of Dysivillosin D (**4**).

**Table S1**. ^1^H and ^13^C NMR Spectroscopic Data for Dysivillosin A (**1**) in Pyr-*d*_5_.*^a^*

| Position | *δ*_H_ (*J* in Hz)*^b^* | *δ*_C_ | HMBC (H→C) | NOESY |
| --- | --- | --- | --- | --- |
| 1α | 2.33, m*^c^* | 19.5, CH_2_ | C-2, 3, 5, 9, 10 | H-2β, 10 |
| 1β | 1.63, m |  | C-2, 3, 5, 10 | H-2α, 10, 12, 14 |
| 2a | 2.38, m | 26.3, CH_2_ | C-3 | H-1α, 2b, 10 |
| 2b | 2.14, m |  | C-1, 3, 4, 10 | H-1α, 1β, 2a |
| 3 | 5.16, brs | 120.4, CH | C-2, 5, 11 | H-2a, 2b, 11 |
| 4 |  | 143.6, C |  |  |
| 5 |  | 38.0, C |  |  |
| 6α | 0.89, td, 13.2, 3.6 | 35.6, CH_2_ | C-4, 5 | H-6β, 8, 10 |
| 6β | 1.49, dt, 12.6, 3.0 |  | C-5, 10 | H-6α, 7β, 11, 12 |
| 7α | 1.33, m | 27.4, CH_2_ | C-6, 13 | H-6α, 7β, 10 |
| 7β | 1.38, dd, 12.6, 1.2 |  | C-5, 6, 9 | H-6α, 6β, 7α, 12, 13 |
| 8 | 1.81, m | 36.0, CH | C-5, 7, 9, 13 | H-6α, 7α, 7β, 10, 13 |
| 9 |  | 41.8, C |  |  |
| 10 | 1.55, d, 12.0 | 45.9, CH | C-1, 4, 5, 6, 9, 12, 14 | H-1α, 1β, 6α, 8 |
| 11 | 1.45, br s | 17.6, CH_3_ | C-3, 4, 5 | H-3, 12 |
| 12 | 1.03, s | 19.6, CH_3_ | C-4, 5, 6, 10 | H-1β, 6β, 7β, 11, 14 |
| 13 | 1.20, d, 6.6 | 17.4, CH_3_ | C-7, 8, 9 | H-7β, 8, 14 |
| 14 | 0.94, s | 17.3, CH_3_ | C-8, 9, 10, 15, 16 | H-1β, 7β, 12, 13 |
| 15 | 3.13, s | 37.8, CH_2_ | C-8, 9, 10, 14, 16, 17, 21 | H-1, 8, 10, 13, 14 |
| 16 |  | 128.4, C |  |  |
| 17 |  | 153.9, C |  |  |
| 18 | 7.74, s | 106.1, CH | C-16, 17, 19, 22 | 17-OH |
| 19 |  | 120.8, C |  |  |
| 20 |  | 147.8, C |  |  |
| 21 | 7.59, s | 114.9, CH | C-15, 17, 20 | H-1α, 8, 10, 13, 15 |
| 22 |  | 117.0, C |  |  |
| 23 | 8.13, dd, 7.8, 1.8 | 129.1 CH | C-22, 25, 27 | H-24 |
| 24 | 7.18, dd, 7.8, 4.8 | 118.4, CH | C-19, 25 | H-23, 25 |
| 25 | 8.46, dd, 5.4, 1.8 | 145.4, CH | C-23, 24, 27 | H-24 |
| 27 |  | 163.5, C |  |  |
| 17-OH | 11.65, br s |  | C-16, 17 | H-18 |

*^a^* 600 MHz for ^1^H NMR and 150 MHz for ^13^C NMR. *^b^* The number of attached protons was determined by analysis of 2D spectroscopic data. *^c^* The coupling constant was not determined because of overlapping signals.

**Table S2**. ^1^H and ^13^C NMR Spectroscopic Data for Dysivillosin B (**2**) in Pyr-*d*_5_.*^a^*

| Position | *δ*_H_ (*J* in Hz)*^b^* | *δ*_C_ | HMBC (H→C) | NOESY |
| --- | --- | --- | --- | --- |
| 1α | 2.35, m*^c^* | 22.8, CH_2_ | C-3, 5, 10 | H-2a, 2b, 3α, 10, 15 |
| 1β | 1.55, dd, 12.0, 2.4 |  | C-2, 3 | H-1α, 2a, 3β, 10, 12, 14 |
| 2a | 1.93, m | 28.0, CH_2_ |  | H-1α, 2b, 3α, 3β |
| 2b | 1.59, m |  | C-10 | H-1α, 2a, 3α, 3β |
| 3α | 2.11, m | 32.6, CH_2_ | C-1, 2, 4, 5, 11 | H-2a, 2b, 11a |
| 3β | 2.36, m |  | C-4, 7, 11 | H-1β, 2a, 2b, 12 |
| 4 |  | 159.4, C |  |  |
| 5 |  | 39.9, C |  |  |
| 6α | 1.29, dd, 13.2, 3.6 | 36.4, CH_2_ | C-5, 9 | H-6β, 8, 10 |
| 6β | 1.45, m |  | C-7, 10 | H-6α, 7β, 11b, 12 |
| 7α | 1.39, m | 27.4, CH_2_ | C-5, 6, 9, 13 | H-6α, 6β, 7β, 8 |
| 7β | 1.42, m |  | C-5, 6, 10 | H-6α, 6β, 7α, 12, 13 |
| 8 | 1.76, m | 36.3, CH | C-6, 7, 9, 13, 14 | H-6α, 7α, 10, 13 |
| 9 |  | 42.2, C |  |  |
| 10 | 1.36, dd, 12.0, 2.4 | 48.2, CH | C-1, 2, 4, 5, 6, 7, 9, 14 | H-1α, 1β, 3α, 6α, 8 |
| 11a | 4.46, s | 102.6, CH_2_ | C-3, 4, 5 | H-3α |
| 11b | 4.38, s |  | C-3, 4, 5 | H-6α, 6β |
| 12 | 1.06, s | 20.0, CH_3_ | C-4, 5, 6, 10 | H-1β, 3β, 6β, 7β, 14 |
| 13 | 1.20, d, 6.6 | 17.4, CH_3_ | C-7, 8, 9 | H-6β, 7β, 8, 14 |
| 14 | 0.93, s | 17.3, CH_3_ | C-8, 9, 10, 15 | H-1β, 7β, 12, 13, 15 |
| 15a | 3.09, d, 14.4 | 37.7, CH_2_ | C-8, 14, 16, 17, 21 | H-1α, 13, 14 |
| 15b | 3.04, d, 14.4 |  | C-9, 10, 14, 16, 17, 21 | H-1α, 13, 14 |
| 16 |  | 128.2, C |  |  |
| 17 |  | 153.8, C |  |  |
| 18 | 7.69, s | 106.1, CH | C-16, 17, 19, 20, 22 | H-23 |
| 19 |  | 120.8, C |  |  |
| 20 |  | 147.8, C |  |  |
| 21 | 7.53, s | 114.6, CH | C-15, 17, 18, 19, 20 |  |
| 22 |  | 117.0, C |  |  |
| 23 | 8.10, dd, 7.8, 1.8 | 129.1, CH | C-19, 25, 27 | H-18, 24 |
| 24 | 7.16, dd, 7.8, 4.8 | 118.4, CH | C-22, 25 | H-23, 25 |
| 25 | 8.45, dd, 4.8, 1.8 | 145.4, CH | C-23, 24, 27 | H-24 |
| 27 |  | 163.5, C |  |  |
| 17-OH | 11.63, br s |  | C-16, 17 |  |

*^a^* 600 MHz for ^1^H NMR and 150 MHz for ^13^C NMR. *^b^* The number of attached protons was determined by analysis of 2D spectroscopic data. *^c^* The coupling constant was not determined because of overlapping signals.

**Table S3**. ^1^H and ^13^C NMR Spectroscopic Data for Dysivillosin C (**3**) in Pyr-*d*_5_.*^a^*

| Position | *δ*_H_ (*J* in Hz)*^b^* | *δ*_C_ | HMBC (H→C) | NOESY |
| --- | --- | --- | --- | --- |
| 1a | 1.57, m*^c^* | 19.1, CH_2_ | C-2, 3, 5, 9, 10 | H-2β, 10 |
| 1b | 2.39, m |  | C-2, 3, 5, 10 | H-2α, 10, 12, 14 |
| 2α | 2.07, m | 26.1, CH_2_ | C-3 | H-1α, 2b, 10 |
| 2β | 2.23, m |  | C-1, 3, 4, 10 | H-1α, 1β, 2a |
| 3 | 4.98, br s | 120.4, CH | C-2, 5, 11 | H-2a, 2b, 11 |
| 4 |  | 143.1, C |  |  |
| 5 |  | 37.8, C |  |  |
| 6α | 0.75, td, 13.2, 3.6 | 35.5, CH_2_ | C-4, 5 | H-6β, 8, 10 |
| 6β | 1.41, dt, 12.6, 3.6 |  | C-5, 10 | H-6α, 7β, 11, 12 |
| 7α | 1.20, dq, 13.2, 3.6 | 27.0, CH_2_ | C-6, 13 | H-6α, 7β, 10 |
| 7β | 1.30, td, 13.2, 3.0 |  | C-5, 6, 9 | H-6α, 6β, 7α, 12, 13 |
| 8 | 1.69, m | 35.9, CH | C-5, 7, 9, 13 | H-6α, 7α, 7β, 10, 13 |
| 9 |  | 41.3, C |  |  |
| 10 | 1.39, dd, 12.6, 1.2 | 45.9, CH | C-1, 4, 5, 6, 9, 12, 14 | H-1α, 1β, 6α, 8 |
| 11 | 1.35, br s | 17.5, CH_3_ | C-3, 4, 5 | H-3, 12 |
| 12 | 0.96, s | 19.5, CH_3_ | C-4, 5, 6, 10 | H-1β, 6β, 7β, 11, 14 |
| 13 | 1.09, d, 6.6 | 16.6, CH_3_ | C-7, 8, 9 | H-7β, 8, 14 |
| 14 | 0.87, s | 17.3, CH_3_ | C-8, 9, 10, 15, 16 | H-1β, 7β, 12, 13 |
| 15a | 3.20, d, 14.4 | 37.2, CH_2_ | C-9, 10, 14, 16, 17, 21 | H-1, 8, 10, 13, 14 |
| 15b | 2.93, d, 14.4 |  | C-8, 10, 14, 16, 17, 21 | H-1, 8, 10, 13, 14 |
| 16 |  | 124.1, C |  |  |
| 17 |  | 147.9, C |  |  |
| 18 |  | 122.3, C |  |  |
| 19 | 7.68, d, 2.4 | 105.0, CH | C-17, 20, 21, 22 | H-23 |
| 20 |  | 154.3, C |  |  |
| 21 | 7.26, d, 2.4 | 120.0, CH | C-15, 17, 19, 20 | H-15a, 15b |
| 22 |  | 117.3, C |  |  |
| 23 | 8.21, dd, 7.2, 1.8 | 129.5, CH | C-18, 25, 27 | H-19, 24 |
| 24 | 7.21, dd, 7.2, 4.8 | 118.6, CH | C-22, 25 | H-23, 25 |
| 25 | 8.52, dd, 4.8, 1.8 | 145.8, CH | C-23, 24, 27 | H-24 |
| 27 |  | 163.3, C |  |  |
| 6′ | 11.67, br s |  |  |  |

*^a^* 600 MHz for ^1^H NMR and 150 MHz for ^13^C NMR. *^b^*The number of attached protons was determined by analysis of 2D spectroscopic data. *^c^* The coupling constant was not determined because of overlapping signals.

**Table S4**. ^1^H and ^13^C NMR Spectroscopic Data for Dysivillosin D (**4**) in Pyr-*d*_5_.*^a^*

| Position | *δ*_H_ (*J* in Hz)*^b^* | *δ*_C_ | HMBC (H→C) | NOESY |
| --- | --- | --- | --- | --- |
| 1α | 2.41, m*^c^* | 22.5, CH_2_ | C-3, 5, 10 | H-2a, 2b, 3α, 10, 15 |
| 1β | 1.53, dd, 12.0, 3.6 |  | C-2, 3 | H-1α, 2a, 3β, 10, 12, 14 |
| 2a | 1.92, m | 27.9, CH_2_ |  | H-1α, 2b, 3α, 3β |
| 2b | 1.35, m |  | C-10 | H-1α, 1β, 2a, 3α, 3β |
| 3α | 1.96, m | 32.5, CH_2_ | C-1, 2, 4, 5, 11 | H-2a, 2b, 11a |
| 3β | 2.26, td, 15.0, 4.8 |  | C-4, 7, 11 | H-1β, 2a, 2b, 12 |
| 4 |  | 159.0, C |  |  |
| 5 |  | 39.8, C |  |  |
| 6α | 1.14, td, 13.2, 3.6 | 36.3, CH_2_ | C-5, 9 | H-6β, 8, 10 |
| 6β | 1.35, m |  | C-7, 10 | H-6α, 7β, 11b, 12 |
| 7α | 1.35, m | 27.0, CH_2_ | C-5, 6, 9, 13 | H-6α, 6β, 7β, 8 |
| 7β | 1.41, m |  | C-5, 6, 10 | H-6α, 6β, 7α, 12, 13 |
| 8 | 1.63, m | 36.2, CH | C-6, 7, 9, 13, 14 | H-6α, 7α, 10, 13 |
| 9 |  | 41.6, C |  |  |
| 10 | 1.20, dd, 12.0, 1.8 | 48.3, CH | C-1, 2, 4, 5, 6, 7, 9, 14 | H-1α, 1β, 3α, 6α, 8 |
| 11a | 4.32, s | 102.6, CH_2_ | C-3, 4, 5 | H-3α |
| 11b | 4.28, s |  | C-3, 4, 5 | H-6α, 6β |
| 12 | 0.99, s | 20.0, CH_3_ | C-4, 5, 6, 10 | H-1β, 3β, 6β, 7β, 14 |
| 13 | 1.10, d, 6.6 | 16.8, CH_3_ | C-7, 8, 9 | H-6β, 7β, 8, 14 |
| 14 | 0.88, s | 17.2, CH_3_ | C-8, 9, 10, 15 | H-1β, 7β, 12, 13, 15 |
| 15a | 3.12, d, 14.4 | 37.3, CH_2_ | C-8, 9, 10, 14, 16, 17, 21 | H-1α, 13, 14 |
| 15b | 2.88, d, 14.4 |  | C-8, 9, 10, 14, 16, 17, 21 | H-1α, 13, 14 |
| 16 |  | 123.9, C |  |  |
| 17 |  | 149.7, C |  |  |
| 18 |  | 122.4, C |  |  |
| 19 | 7.63, d, 3.0 | 105.1, C | C-17, 20, 21, 22 | H-23 |
| 20 |  | 154.2, C |  |  |
| 21 | 7.20, d, 3.0 | 119.7, CH | C-15, 17, 19, 20 | H-15 |
| 22 |  | 117.2, C |  |  |
| 23 | 8.18, dd, 7.8, 1.8 | 129.4, CH | C-18, 25, 27 | H-19, 24 |
| 24 | 7.16, dd, 7.8, 4.8 | 118.5, CH | C-22, 25 | H-23, 25 |
| 25 | 8.50, dd, 4.8, 1.8 | 145.8, CH | C-23, 24, 27 | H-24 |
| 27 |  | 163.3, C |  |  |
| 17-OH | 11.63, br s |  |  |  |

*^a^* 600 MHz for ^1^H NMR and 150 MHz for ^13^C NMR. *^b^*The number of attached protons was determined by analysis of 2D spectroscopic data. *^c^* The coupling constant was not determined because of overlapping signals.

**Energy Minimization and ECD Calculations.**

The quantum chemical ECD calculation method was used to establish the absolute configuration of dysivillosin A (**1**). The preliminary conformational distribution search was performed by Syby18.0 software using the Tripos force field overlaid with key correlations observed in the NOESY spectra of **1**. The corresponding minimum geometries were further fully optimized by using DFT at B3LYP/6-31G(d) level as implemented in the Gaussian 09 program package. The stable conformers obtained were submitted to ECD calculation by TDDFT [B3LYP/6-31++G(2d,3p)] method. The overall predicted ECD spectra of **1** were subsequently compared with the experimental ones.

The initial conformations of **1** were optimized using MMFF94 method in MarvinSketch 5.8.1 and then HF/6-31G(d) method in Gaussian 09.^1^ Further optimized at the B3P86/6-31G(d) level led the dihedral angles to be got. The optimized conformations were taken for the ECD calculations, which were performed with Gaussian09 (B3P86/6-311++G(2d,p)). The solvent effects were taken into account by the polarizable-conductor calculation model (CPCM, methanol as the solvent).

**References:**

(1) Frisch, M. J.; Trucks, G. W.; Schlegel, H. B.; Scuseria, G. E.; Robb, M. A.; Cheeseman, J. R.; Scalmani, G.; Barone, V.; Mennucci, B.; Petersson, G. A.; Nakatsuji, H.; Caricato, M.; Li, X.; Hratchian, H. P.; Izmaylov, A. F.; Bloino, J.; Zheng, G.; Sonnenberg, J. L.; Hada, M.; Ehara, M.; Toyota, K.; Fukuda, R.; Hasegawa, J.; Ishida, M.; Nakajima, T.; Honda, Y.; Kitao, O.; Nakai, H.; Vreven, T.; Montgomery, J. A., Jr.; Peralta, J. E.; Ogliaro, F.; Bearpark, M.; Heyd, J. J.; Brothers, E.; Kudin, K. N.; Staroverov, V. N.; Keith, T.; Kobayashi, R.; Normand, J.; Raghavachari, K.; Rendell, A.; Burant, J. C.; Iyengar, S. S.; Tomasi, J.; Cossi, M.; Rega, N.; Millam, J. M.; Klene, M.; Knox, J. E.; Cross, J. B.; Bakken, V.; Adamo, C.; Jaramillo, J.; Gomperts, R.; Stratmann, R. E.; Yazyev, O.; Austin, A. J.; Cammi, R.; Pomelli, C.; Ochterski, J. W.; Martin, R. L.; Morokuma, K.; Zakrzewski, V. G.; Voth, G. A.; Salvador, P.; Dannenberg, J. J.; Dapprich, S.; Daniels, A. D.; Farkas, O.; Foresman, J. B.; Ortiz, J. V.; Cioslowski, J.; Fox, D. J. *Gaussian 09, Revision B.01*;

Gaussian, Inc.: Wallingford, CT, 2010.

**Figure S1.** Key 2D NMR correlations of dysivillosins B−D (**2**−**4**).


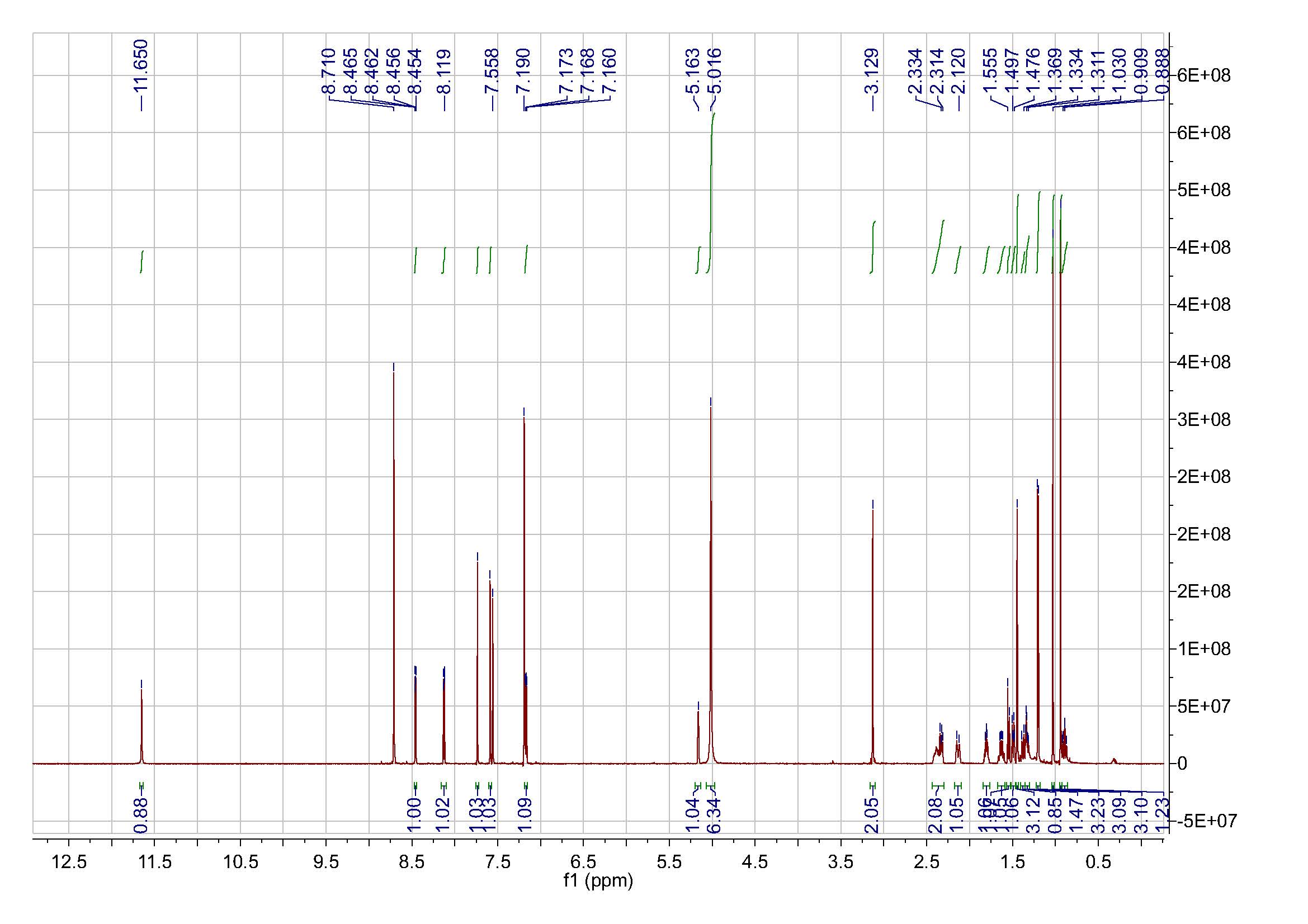


**Figure S2.** ^1^H NMR Spectrum of Dysivillosin A (**1**) in Pyr-*d*_5_.


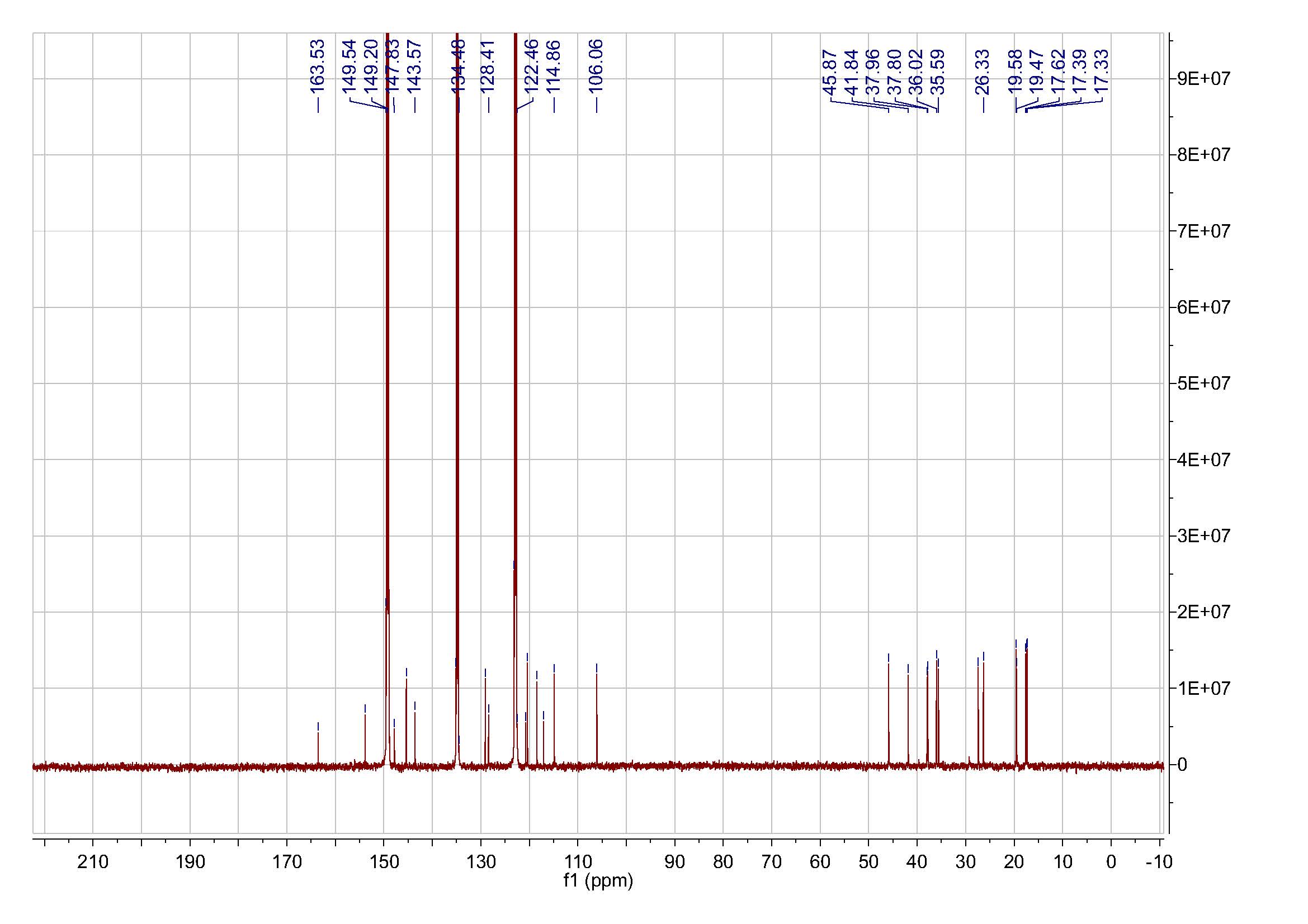


**Figure S3.** ^13^C NMR Spectrum of Dysivillosin A (**1**) in Pyr-*d*_5_.


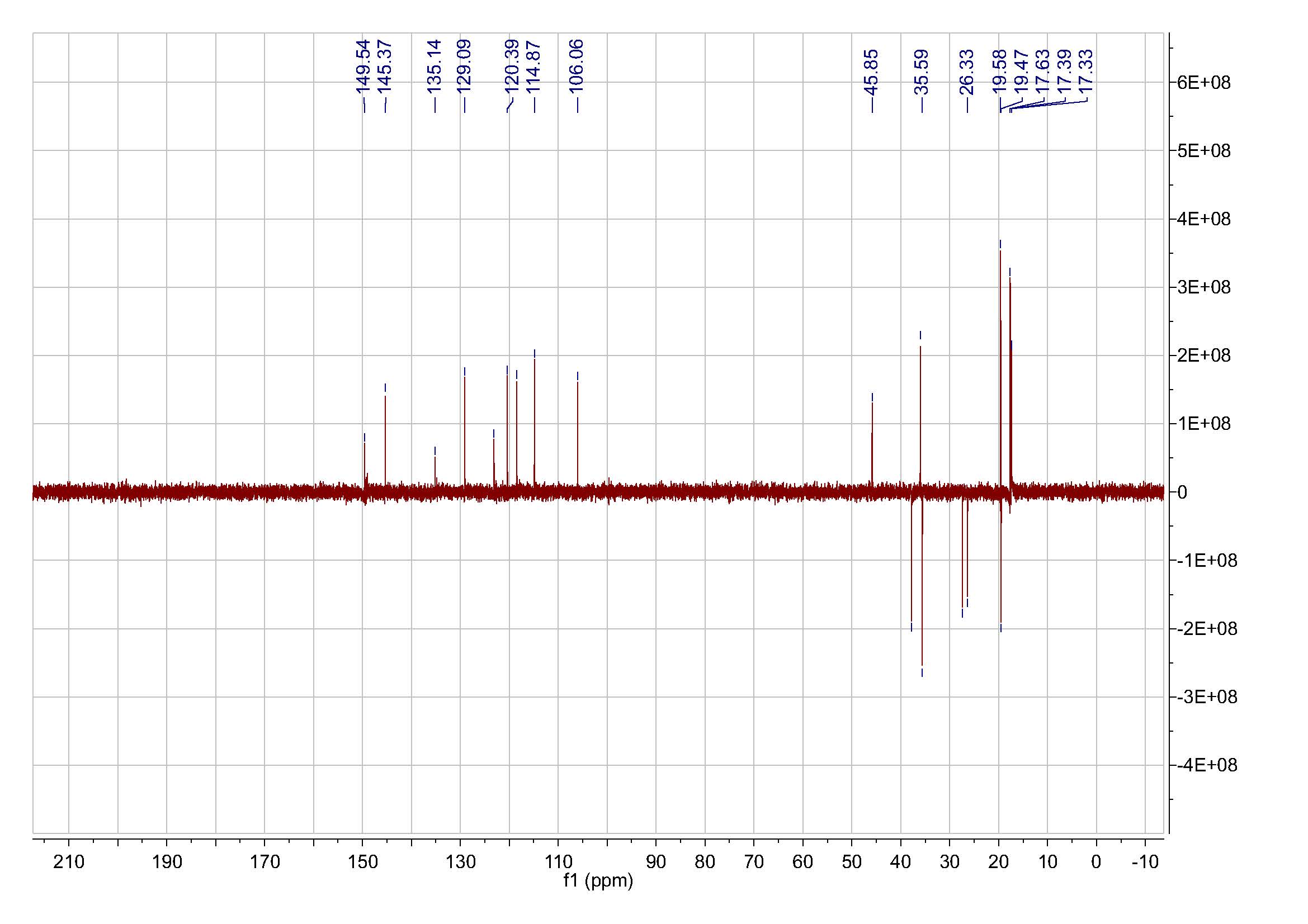


**Figure S4.** DEPT135 Spectrum of Dysivillosin A (**1**) in Pyr-*d*_5_.


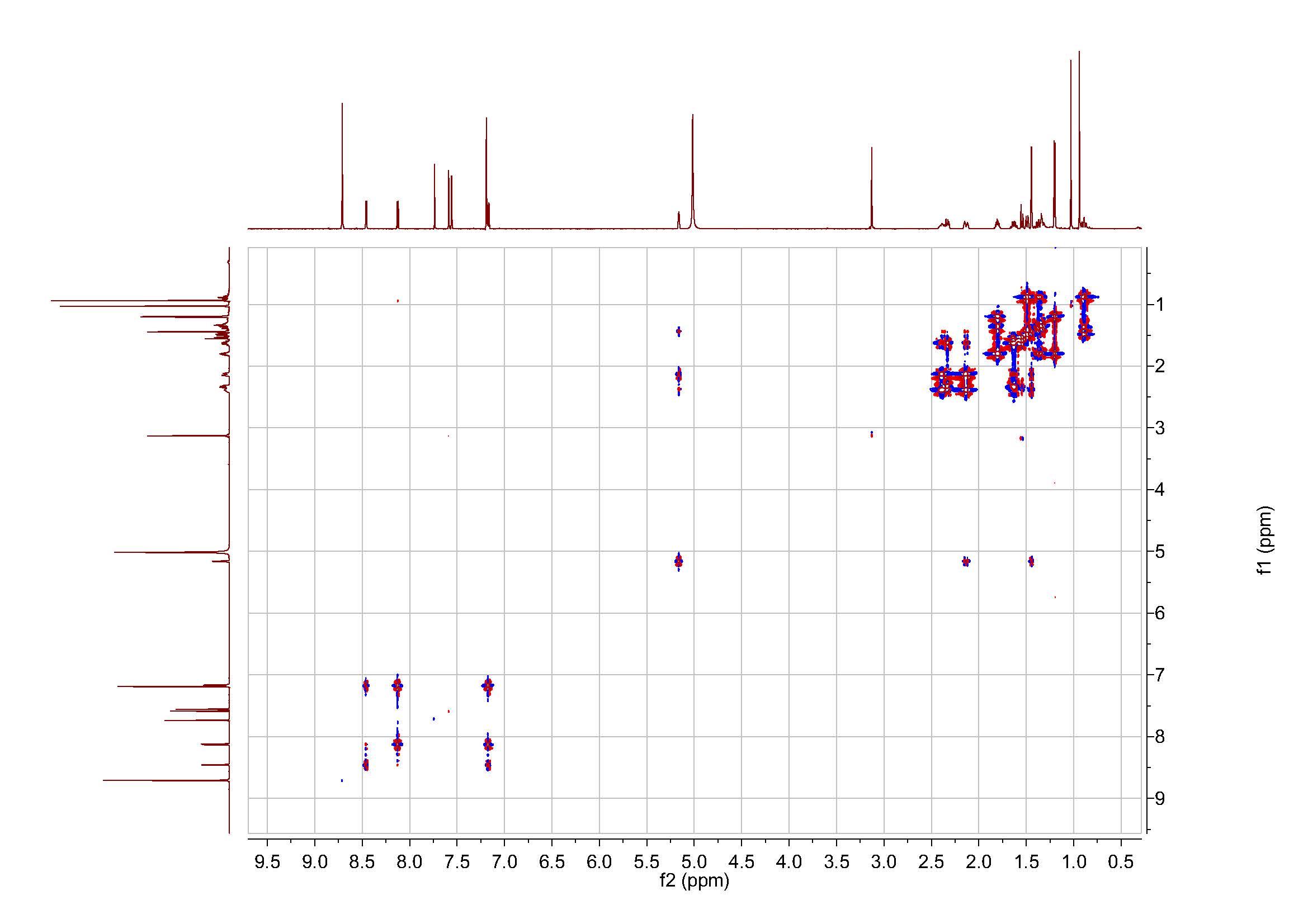


**Figure S5.** ^1^H-^1^H COSY Spectrum of Dysivillosin A (**1**) in Pyr-*d*_5_.


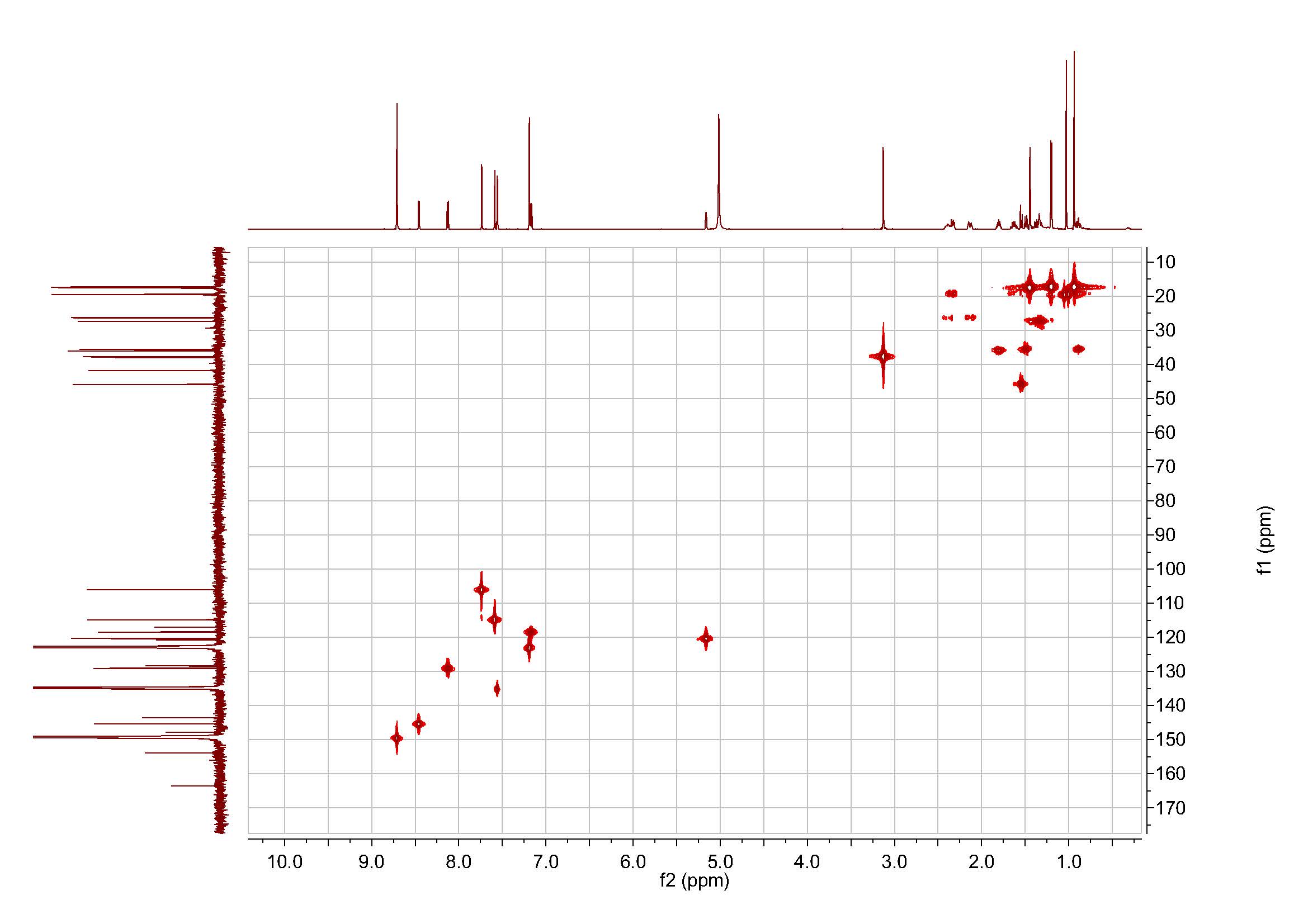


**Figure S6.** HSQC Spectrum of Dysivillosin A (**1**) in Pyr-*d*_5_.


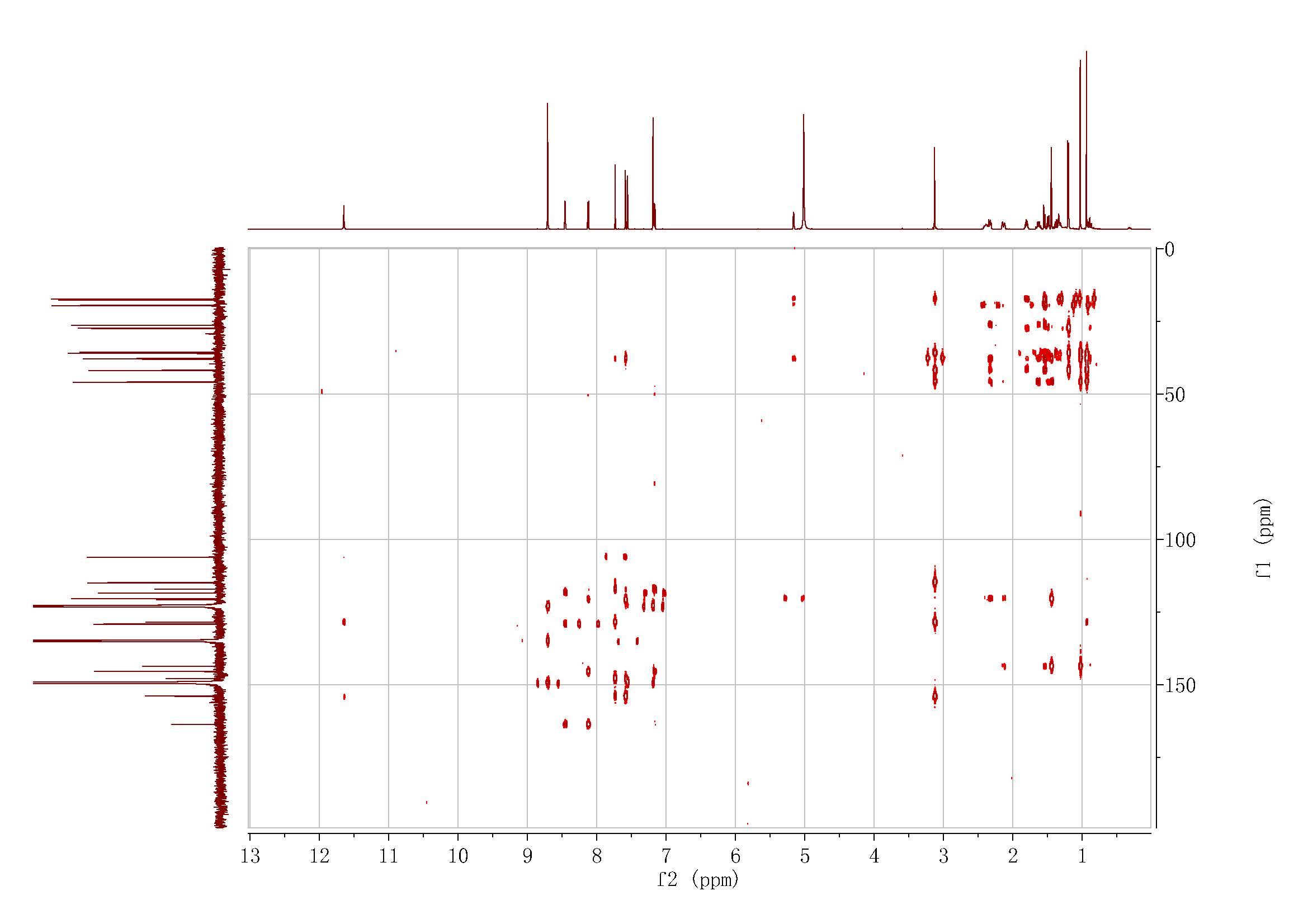


**Figure S7.** HMBC Spectrum of Dysivillosin A (**1**) in Pyr-*d*_5_.


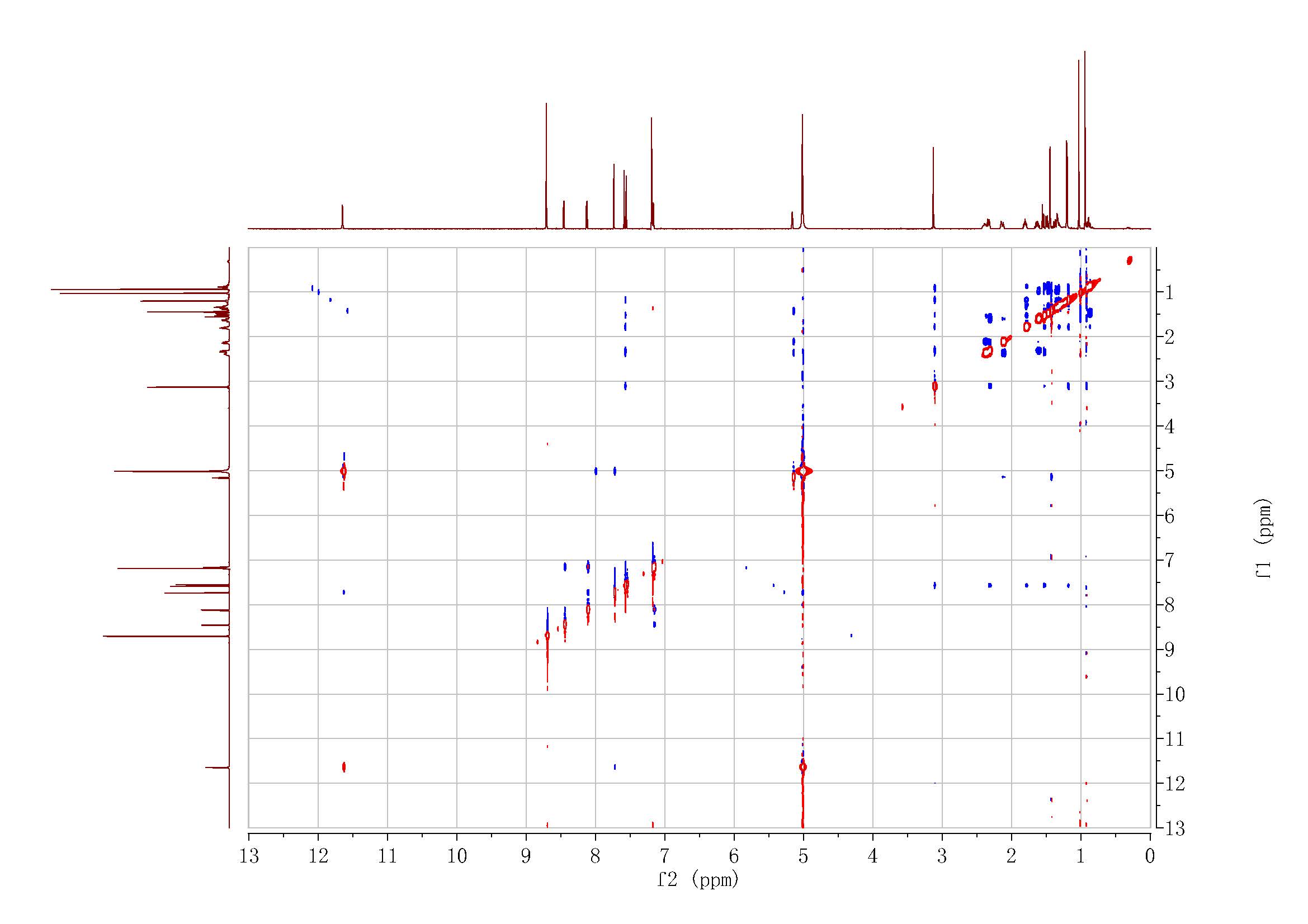


**Figure S8.** NOESY Spectrum of Dysivillosin A (**1**) in Pyr-*d*_5_.


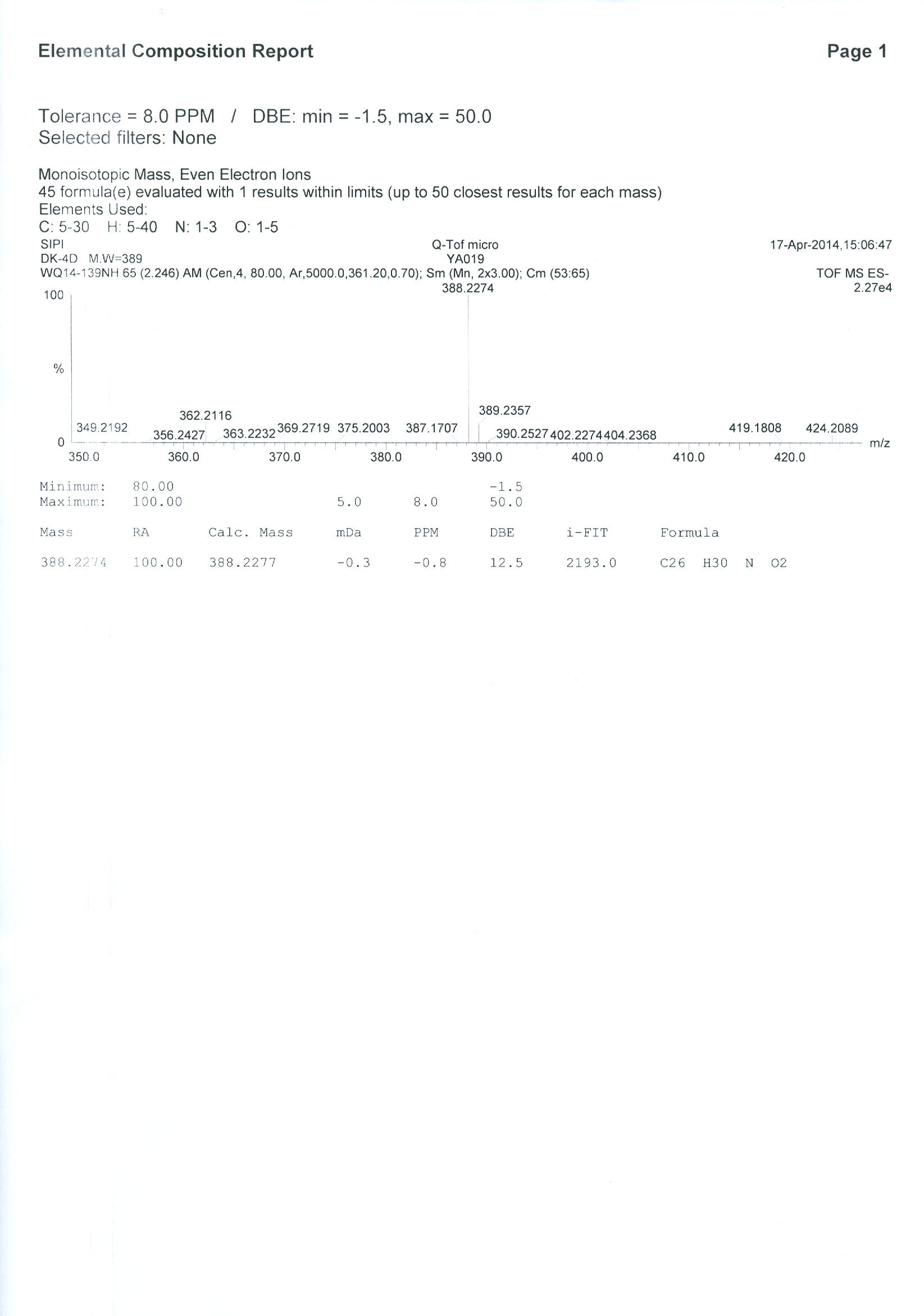


**Figure S9.** HRESIMS of Dysivillosin A (**1**).


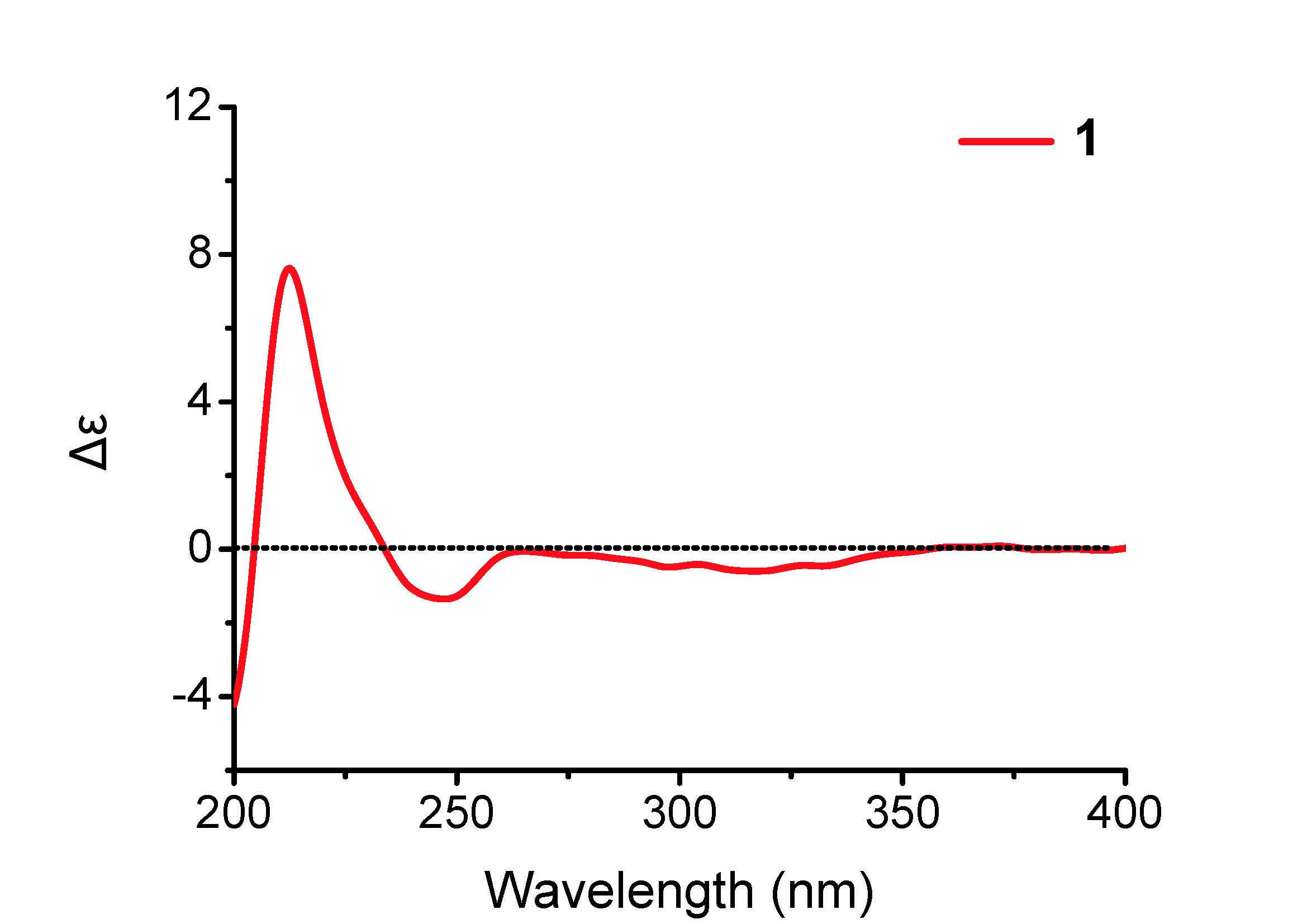


**Figure S10.** Experimental ECD Spectrum of Dysivillosin A (**1**) in MeOH.


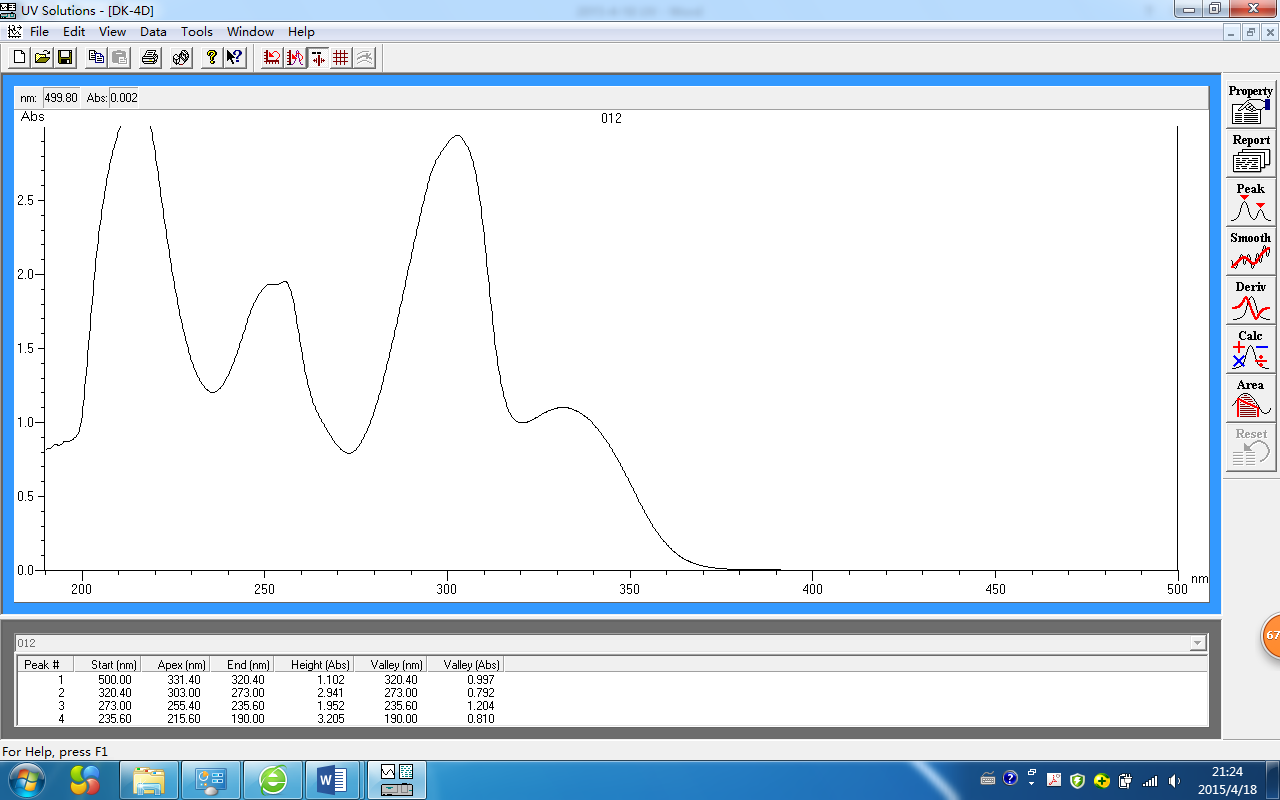


**Figure S11.** UV Spectrum of Dysivillosin A (**1**) in MeOH.


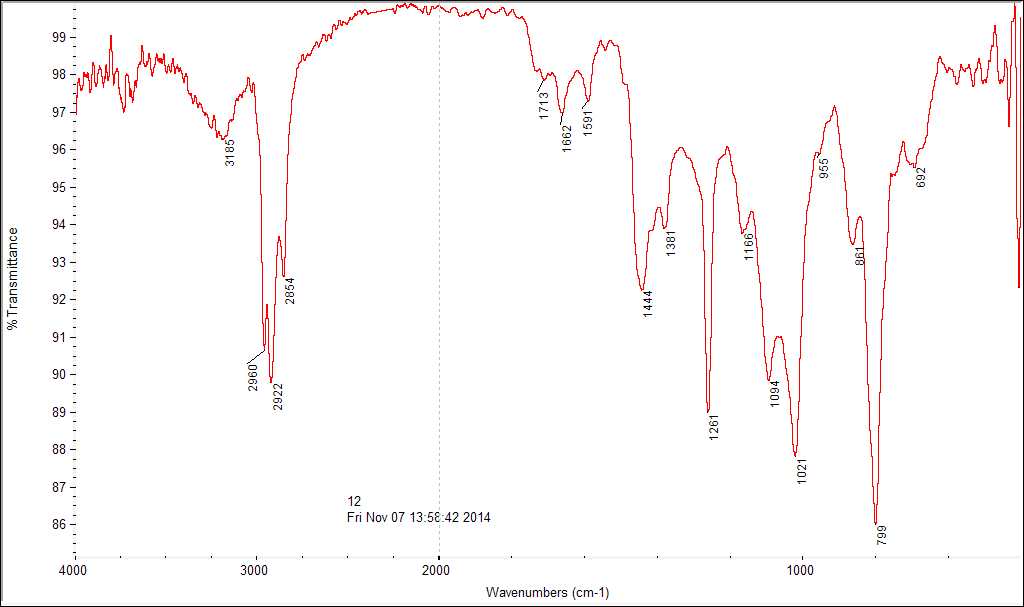


**Figure S12.** IR Spectrum of Dysivillosin A (**1**).


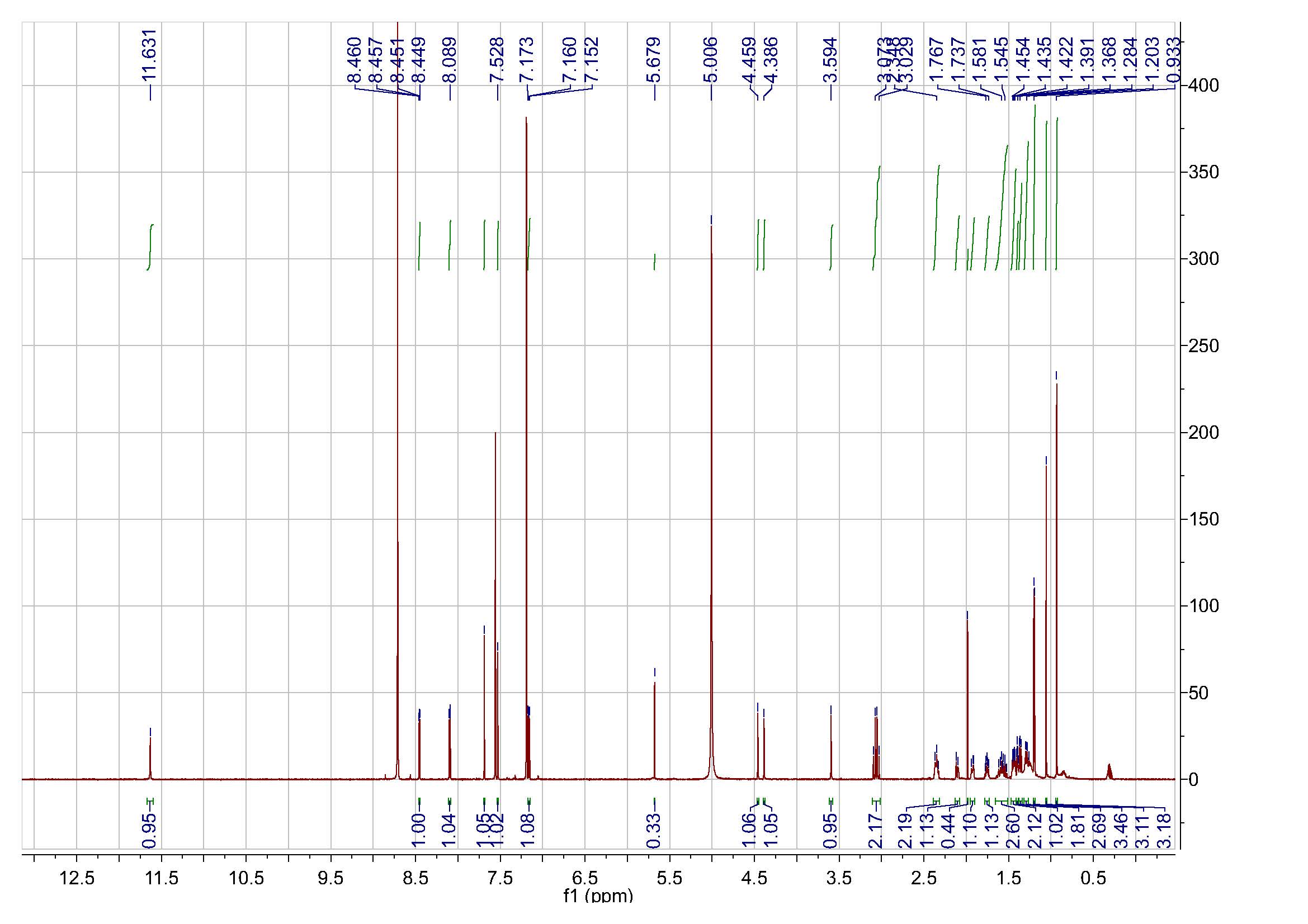


**Figure S13.** ^1^H NMR Spectrum of Dysivillosin B (**2**) in Pyr-*d*_5_.


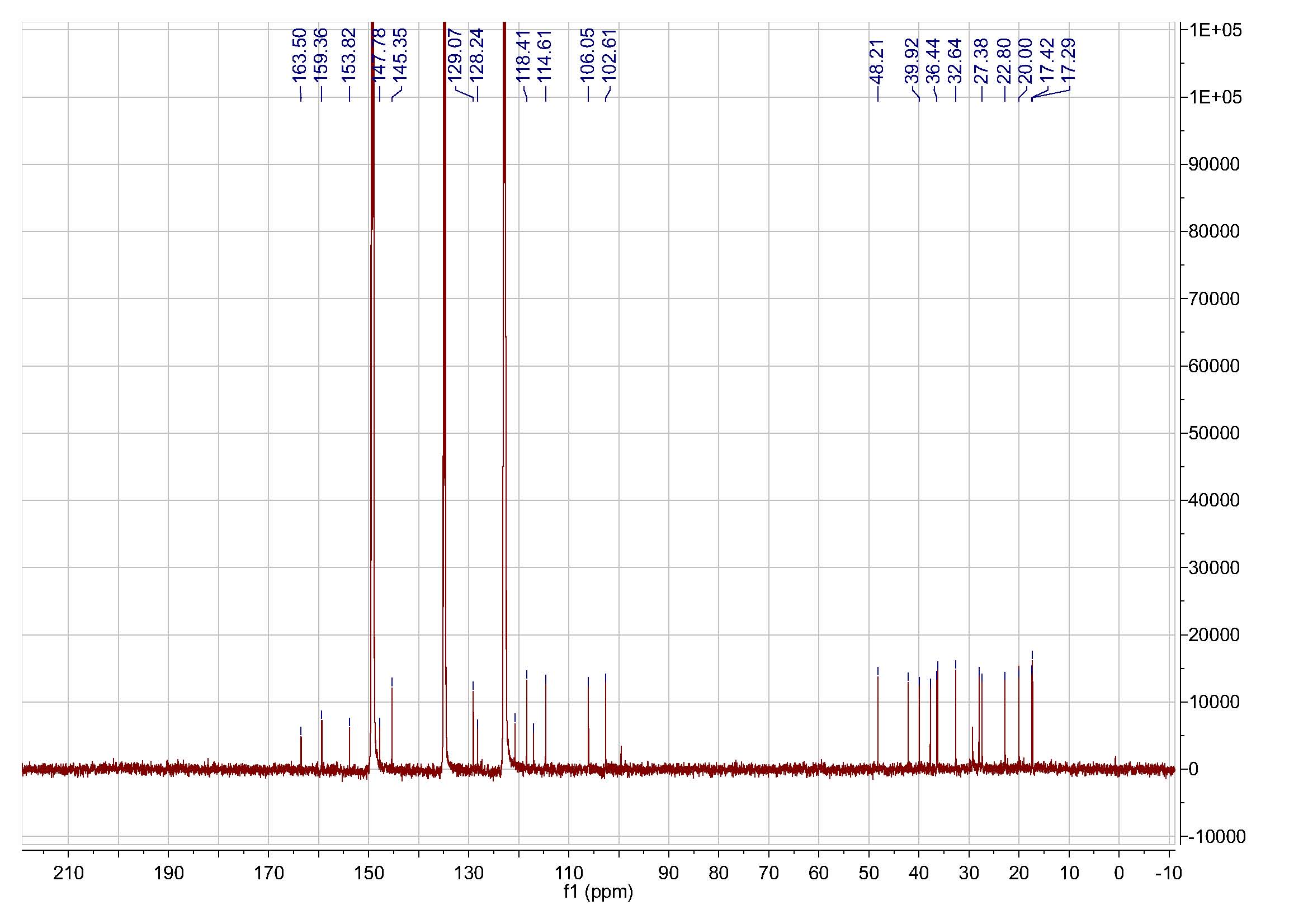


**Figure S14.** ^13^C NMR Spectrum of Dysivillosin B (**2**) in Pyr-*d*_5_.


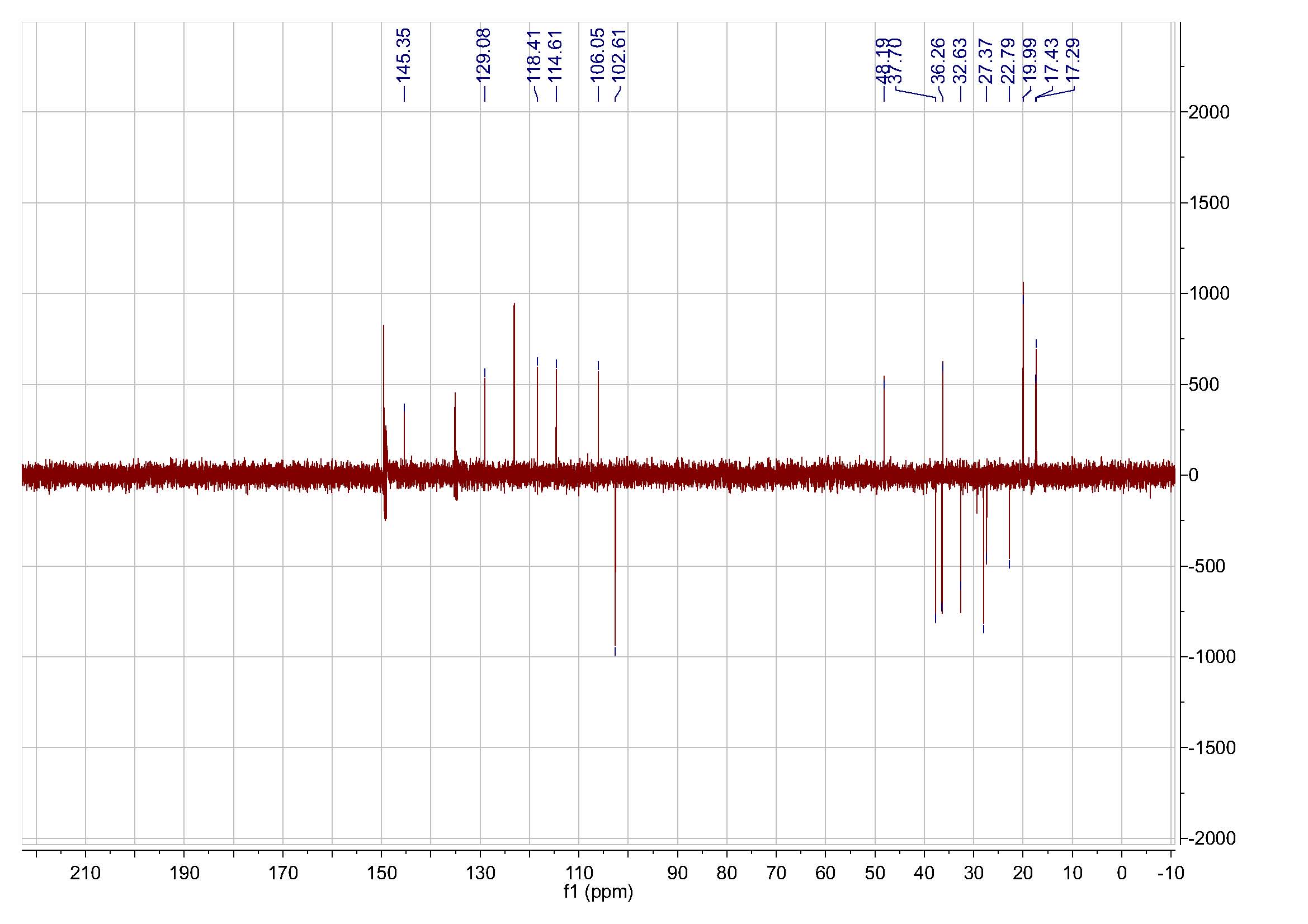


**Figure S15.** DEPT135 Spectrum of Dysivillosin B (**2**) in Pyr-*d*_5_.


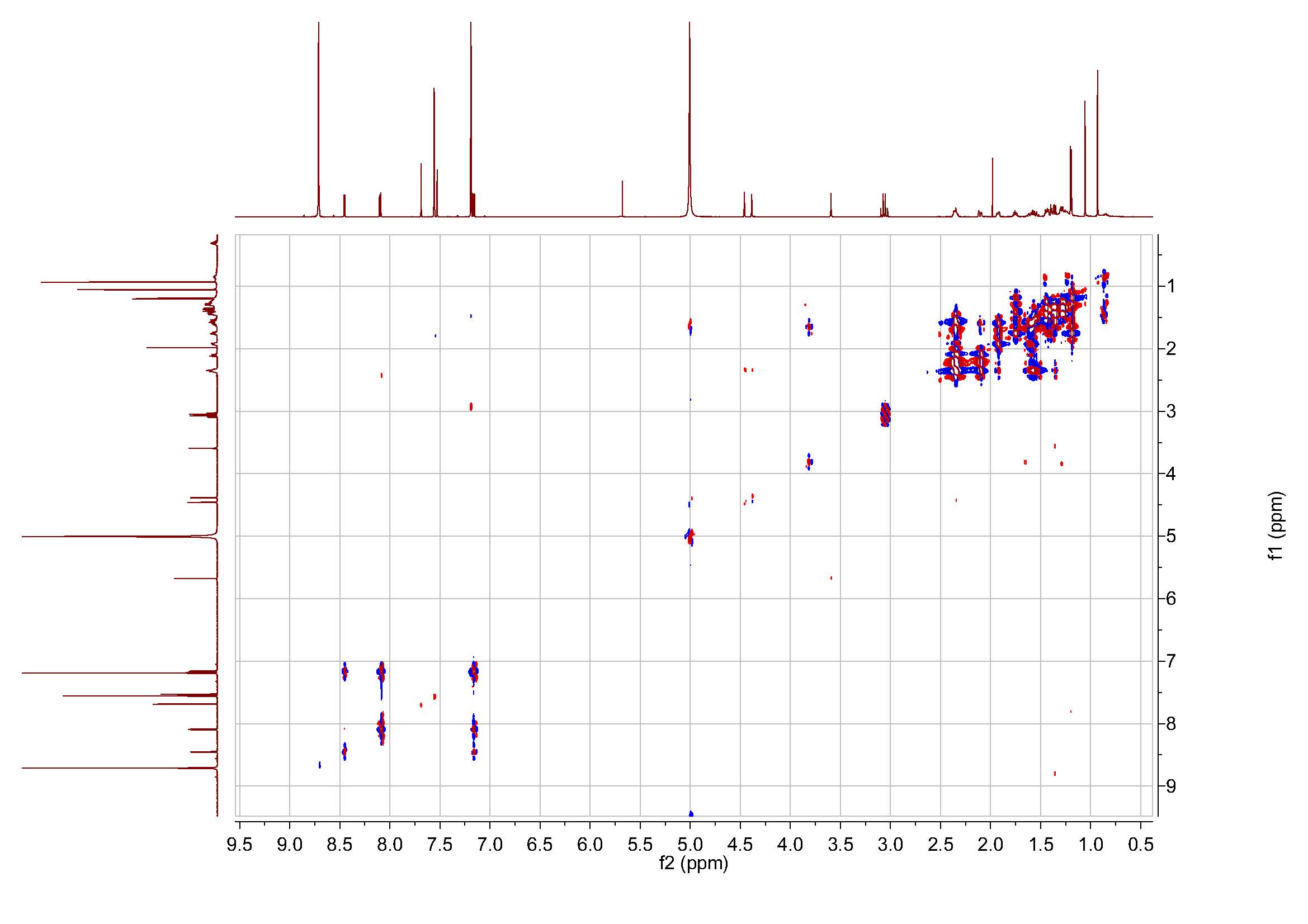


**Figure S16.** ^1^H-^1^H COSY Spectrum of Dysivillosin B (**2**) in Pyr-*d*_5_.


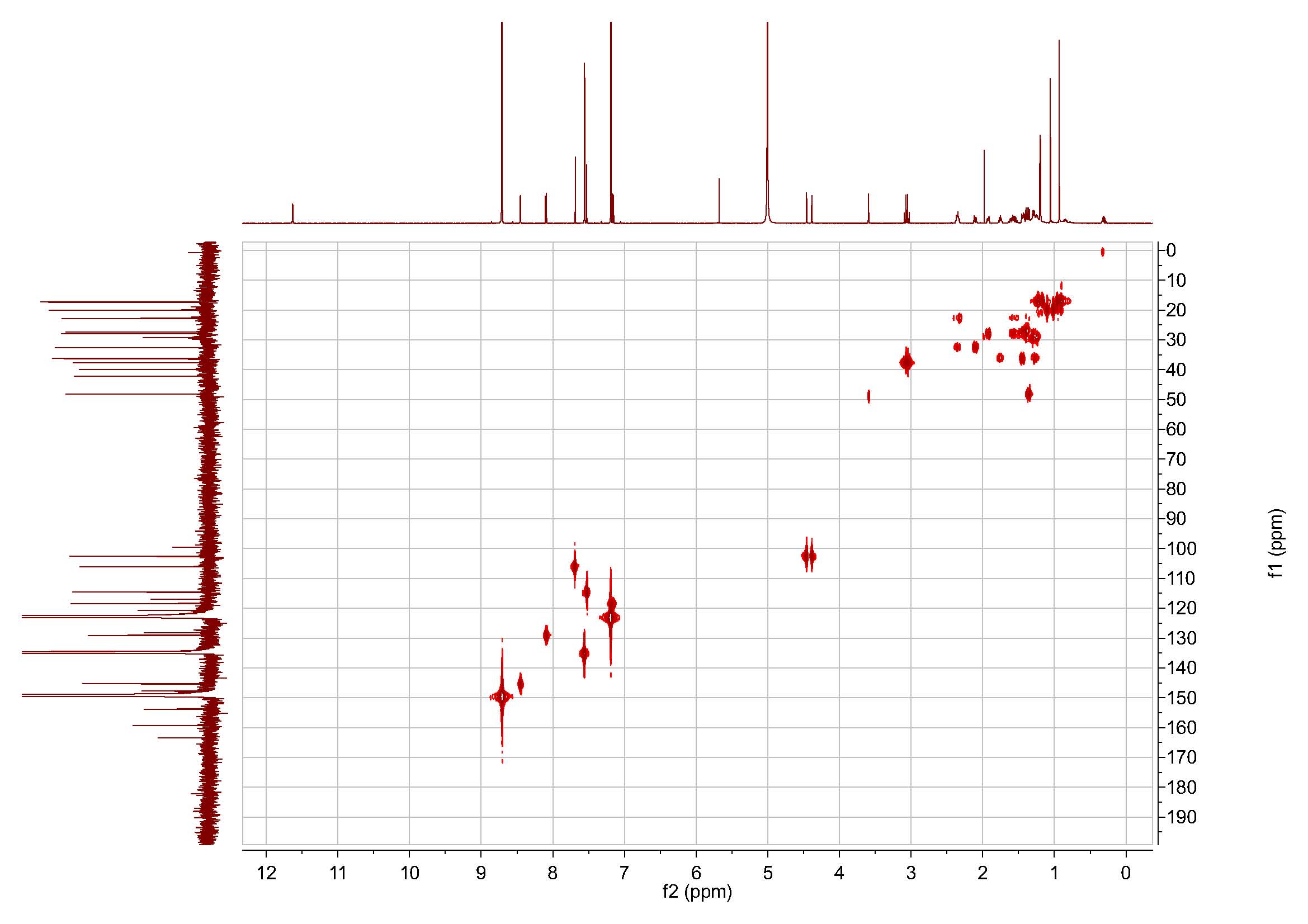


**Figure S17.** HSQC Spectrum of Dysivillosin B (**2**) in Pyr-*d*_5_.


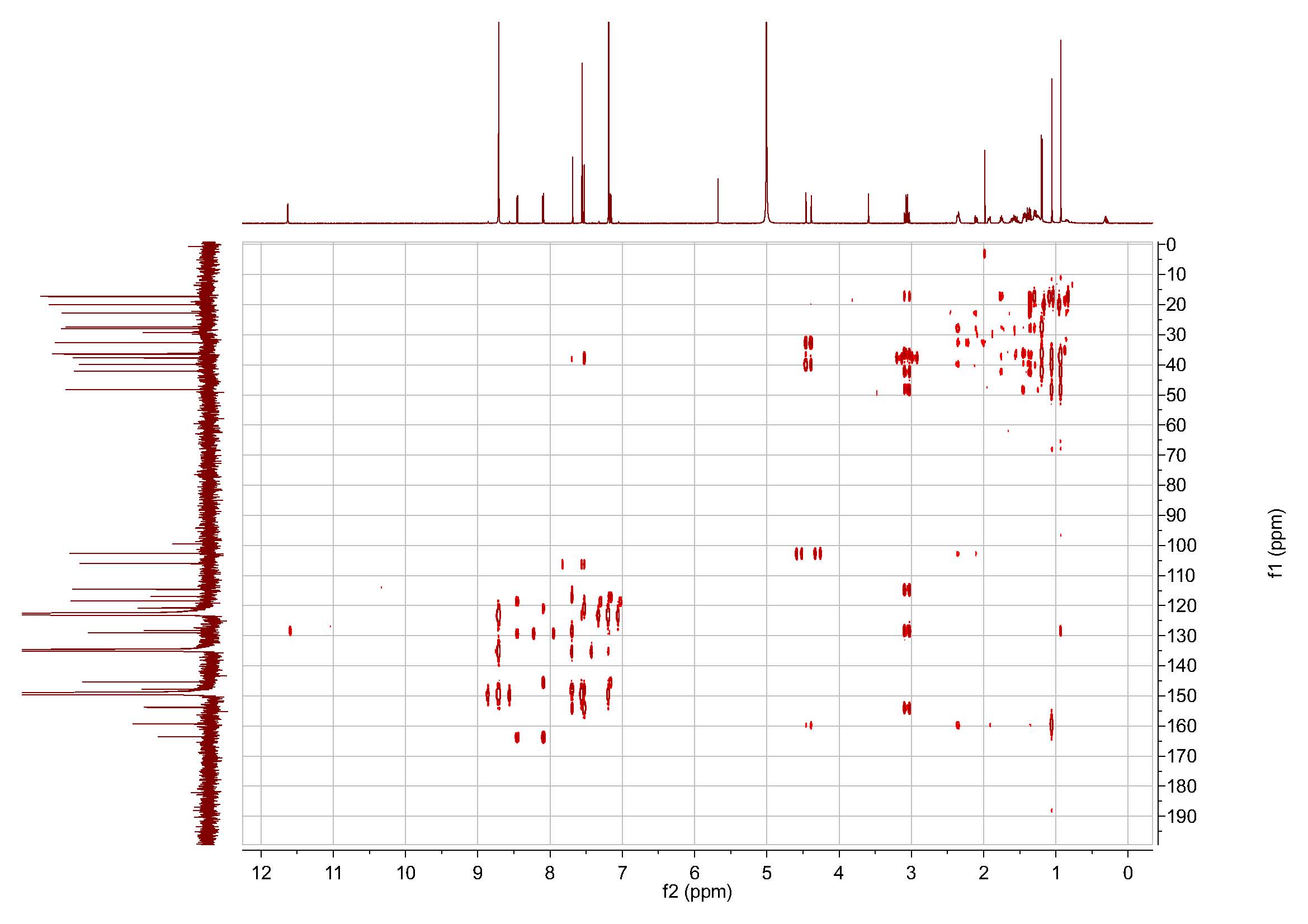


**Figure S18.** HMBC Spectrum of Dysivillosin B (**2**) in Pyr-*d*_5_.


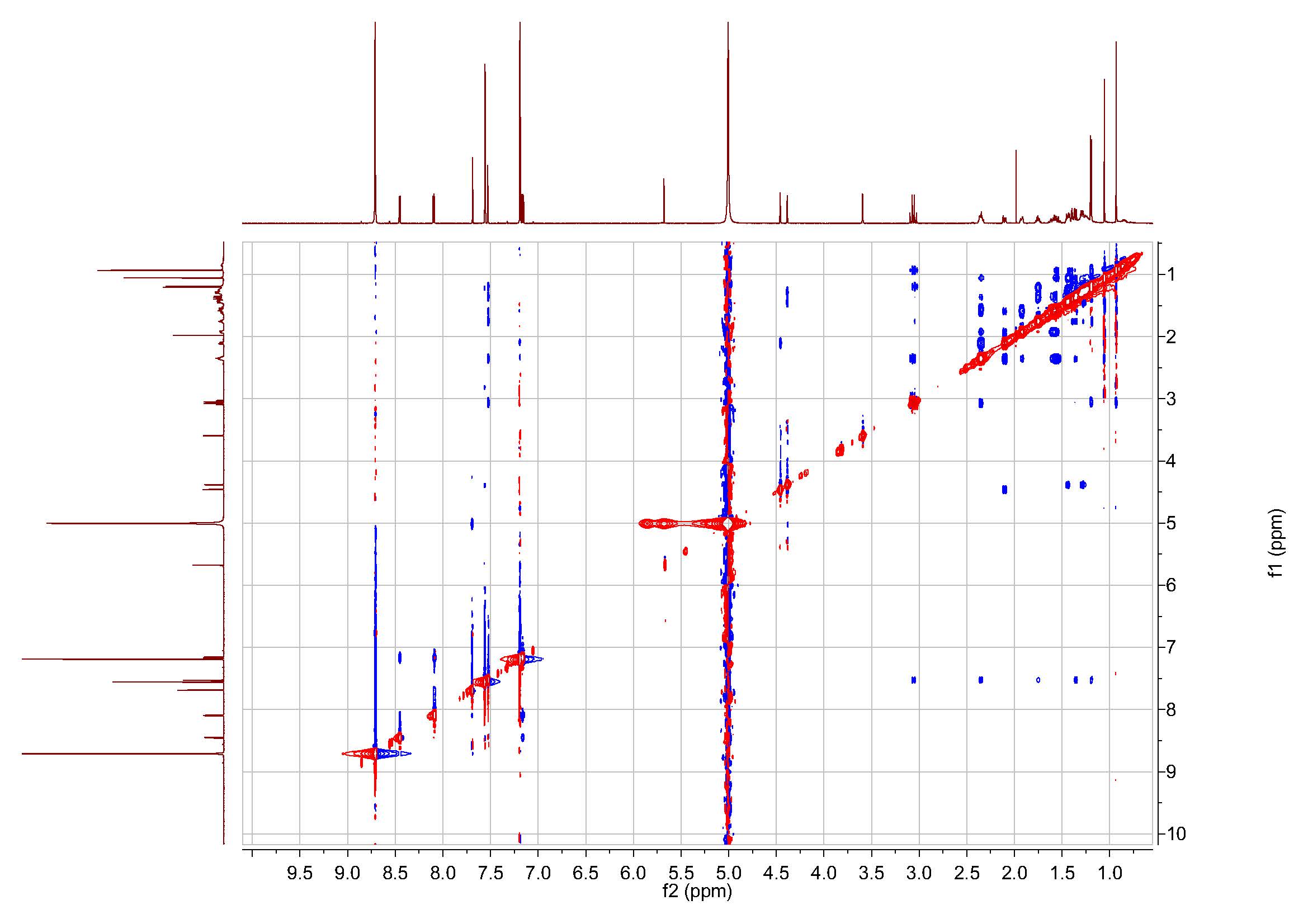


**Figure S19.** NOESY Spectrum of Dysivillosin B (**2**) in Pyr-*d*_5_.


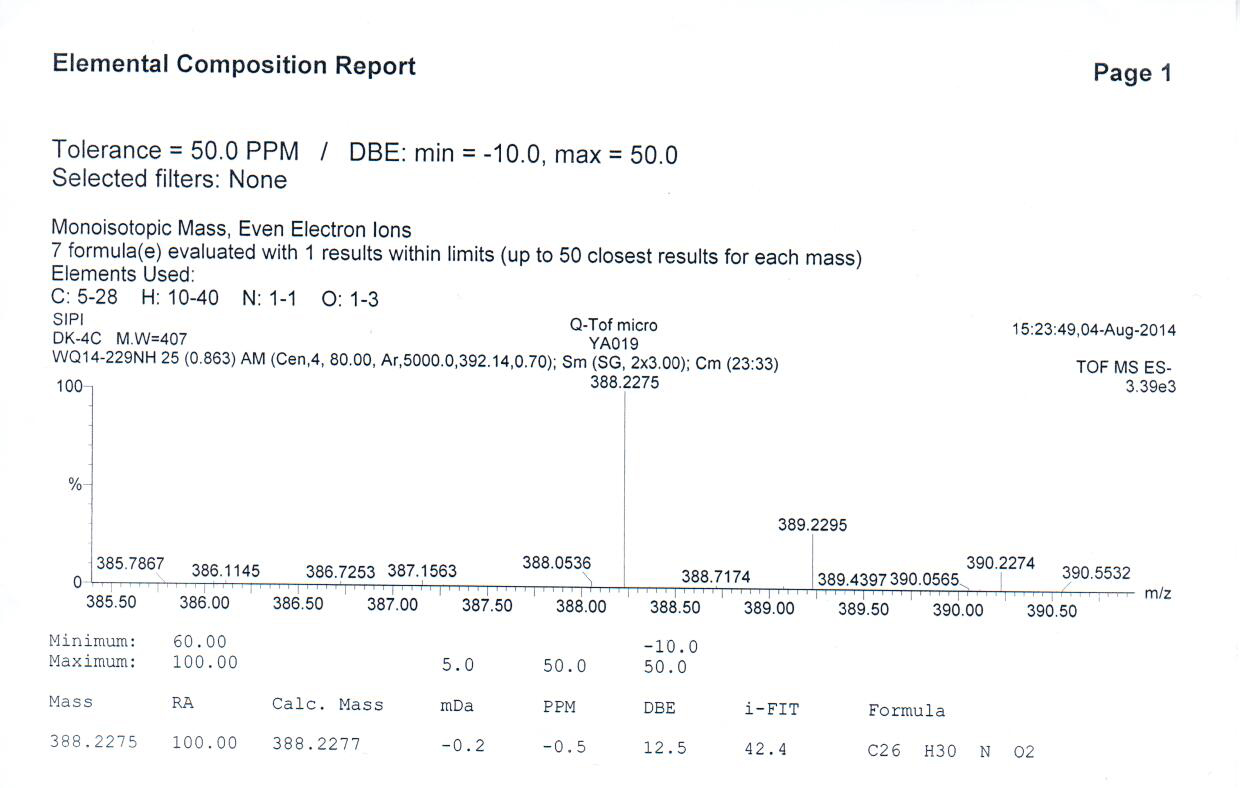


**Figure S20.** HRESIMS of Dysivillosin B (**2**).


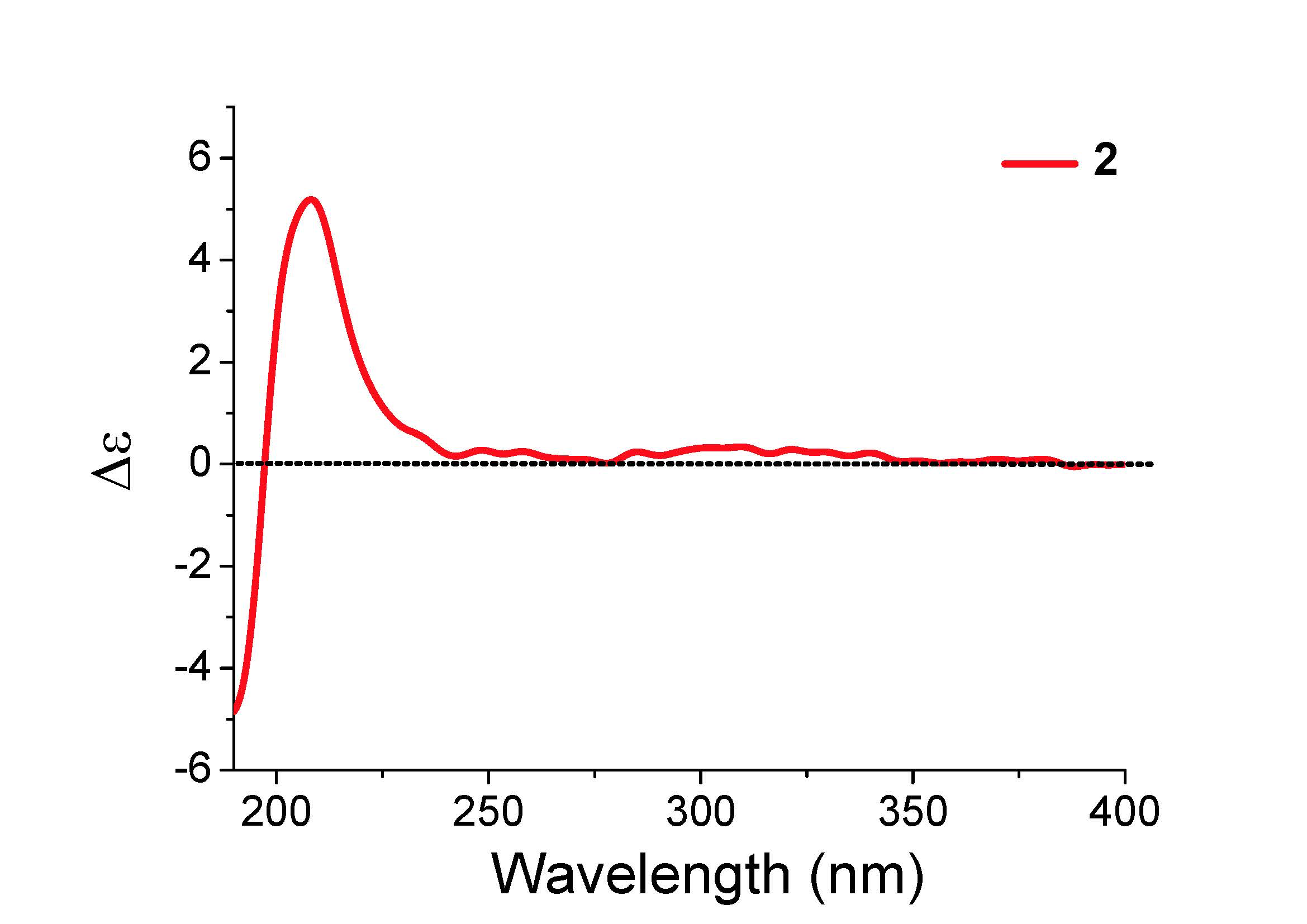


**Figure S21.** Experimental ECD Spectrum of Dysivillosin B (**2**) in MeOH.


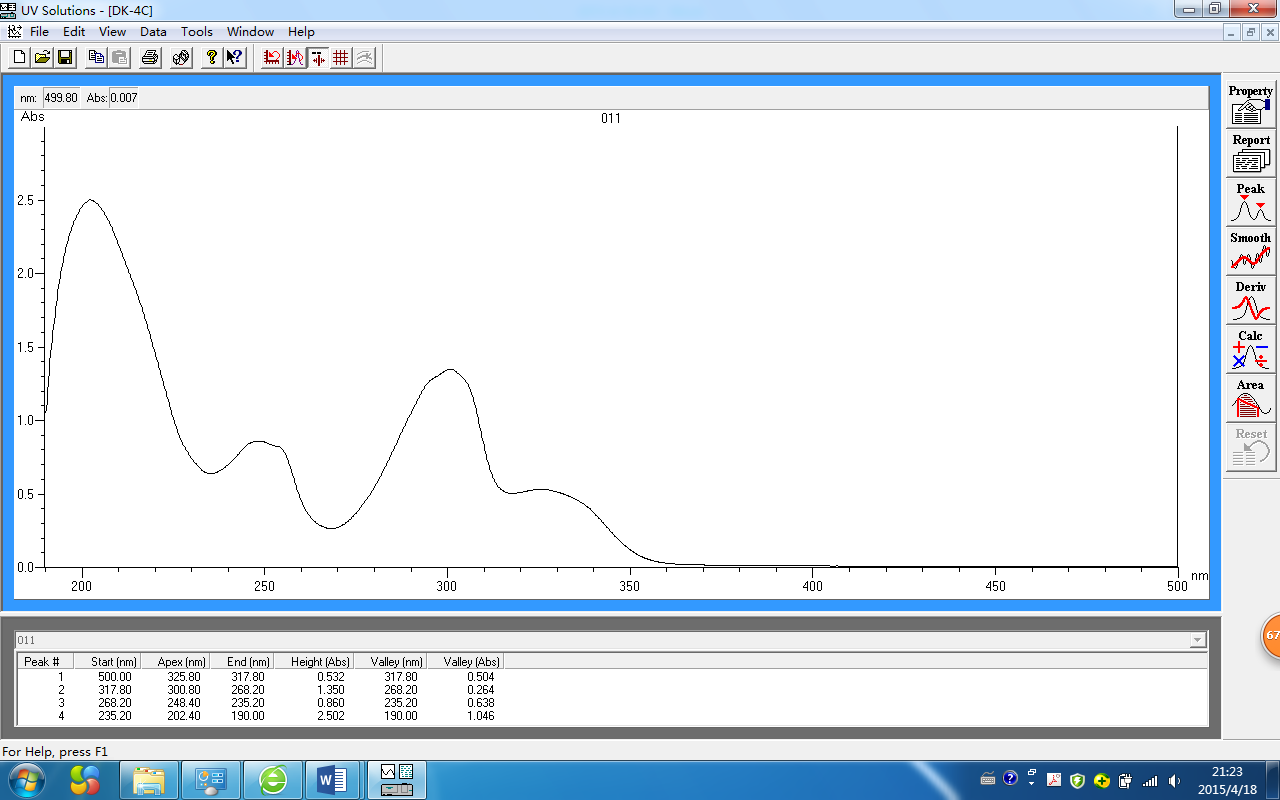


**Figure S22.** UV Spectrum of Dysivillosin B (**2**) in MeOH.


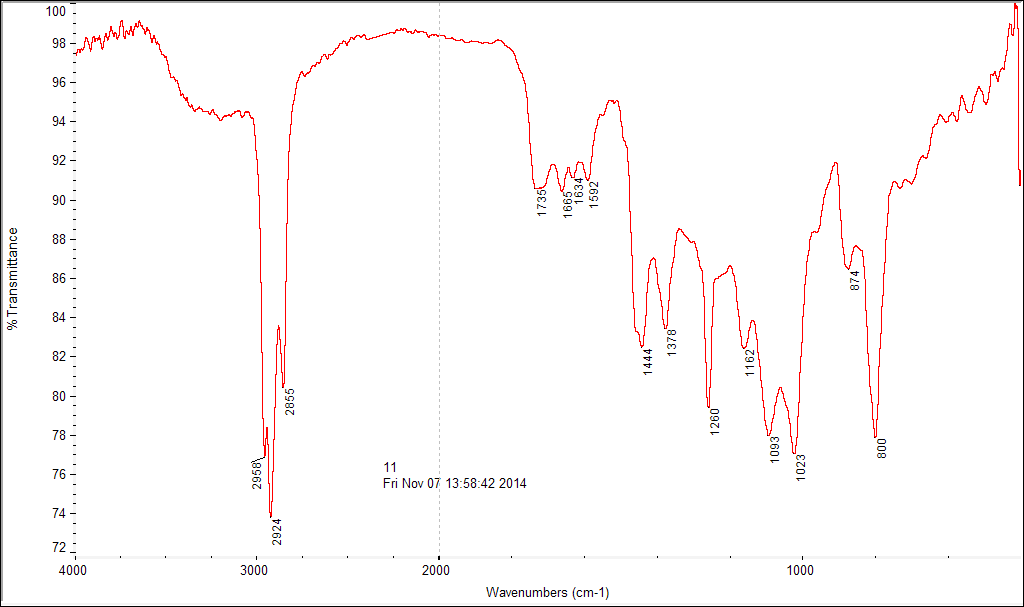


**Figure S23.** IR Spectrum of Dysivillosin B (**2**).


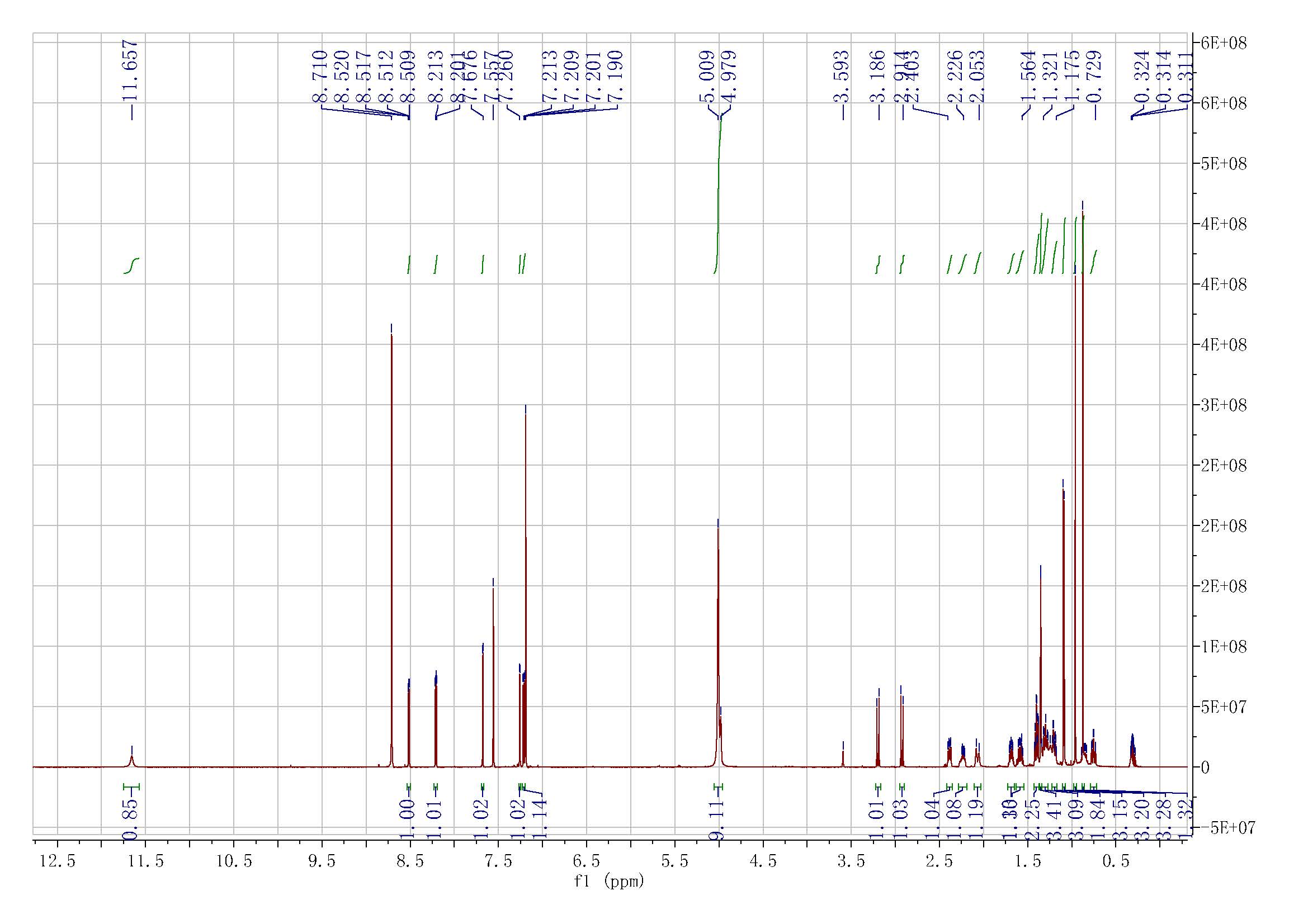


**Figure S24.** ^1^H NMR Spectrum of Dysivillosin C (**3**) in Pyr-*d*_5_.


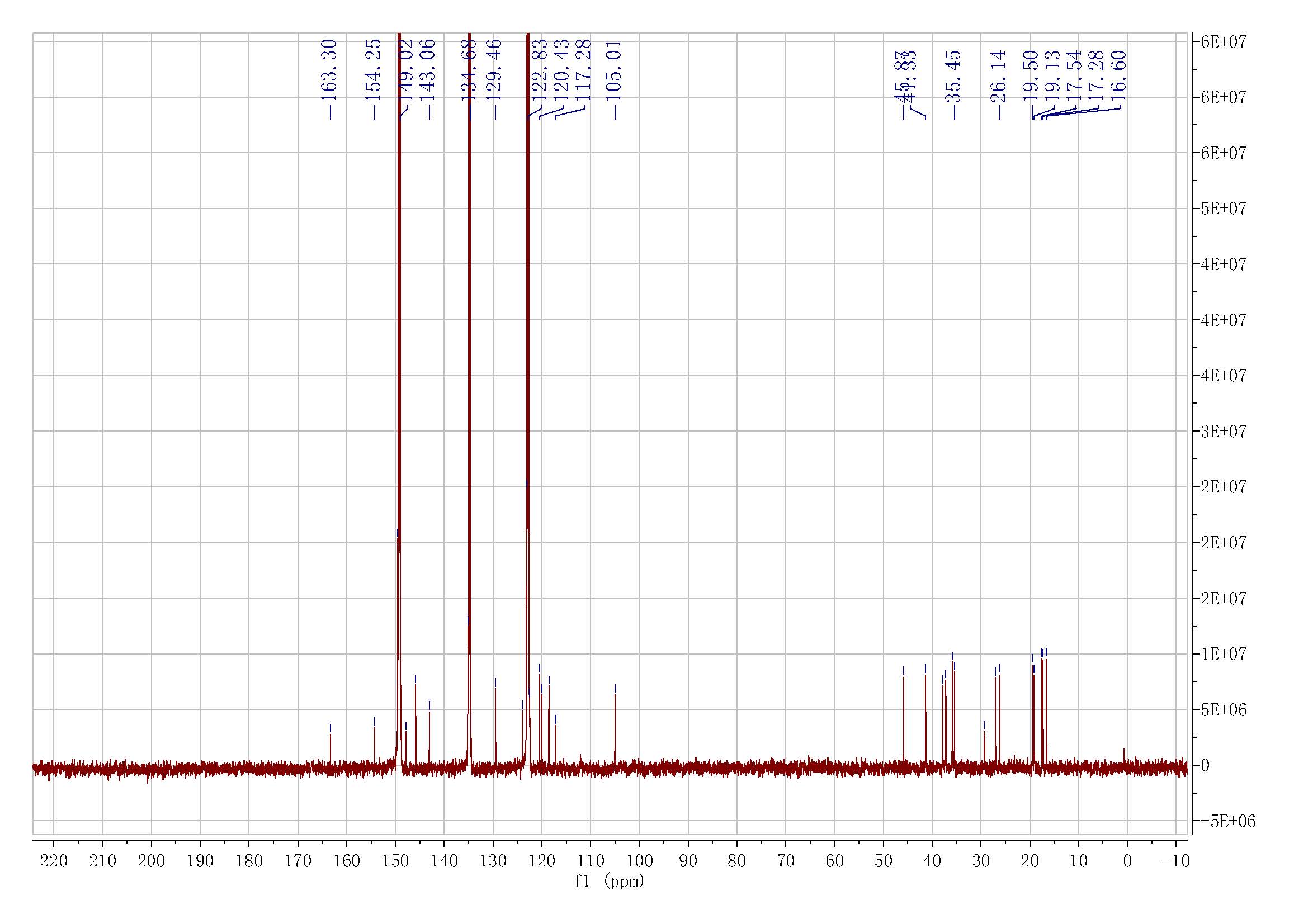


**Figure S25.** ^13^C NMR Spectrum of Dysivillosin C (**3**) in Pyr-*d*_5_.


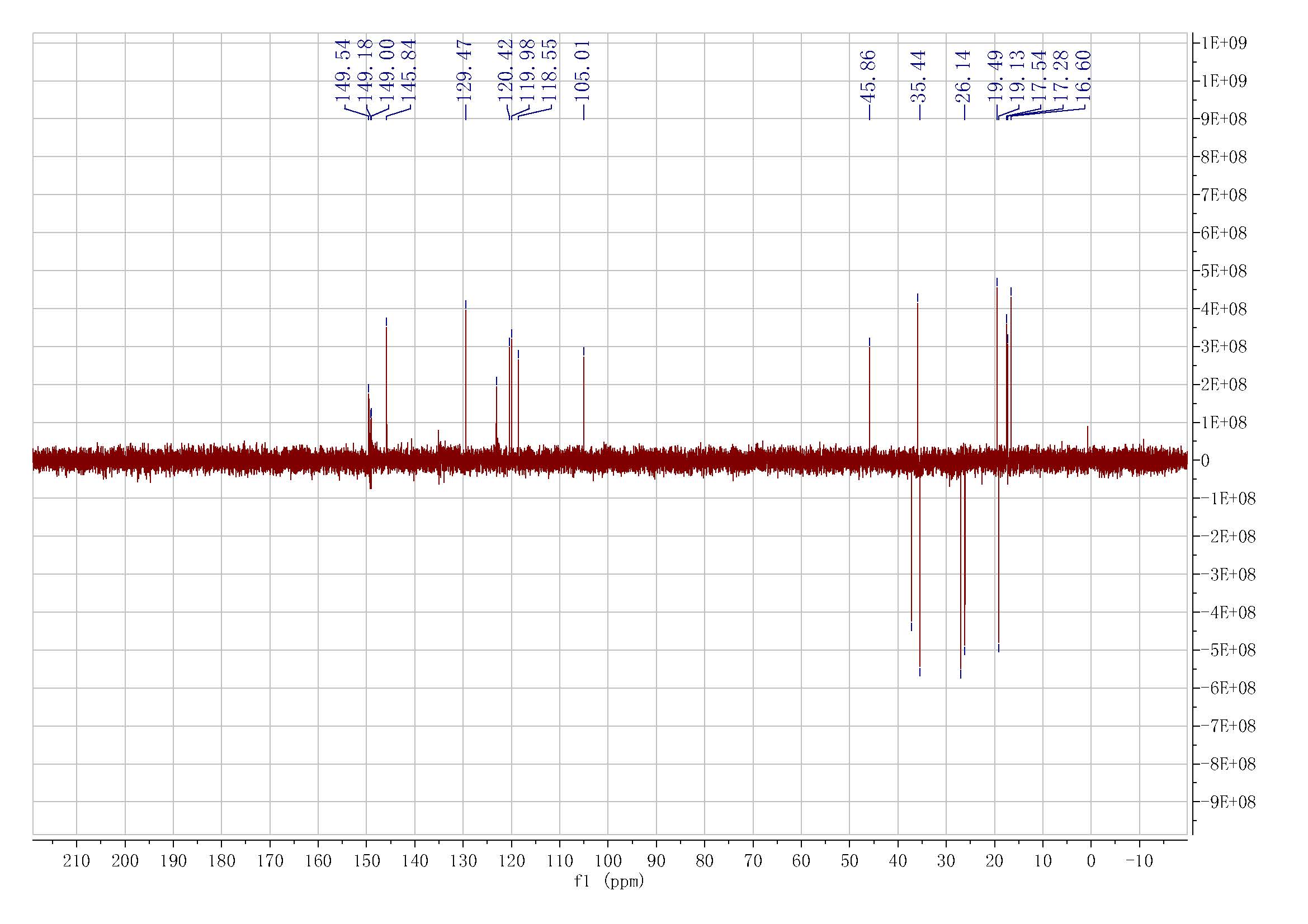


**Figure S26.** DEPT135 Spectrum of Dysivillosin C (**3**) in Pyr-*d*_5_.


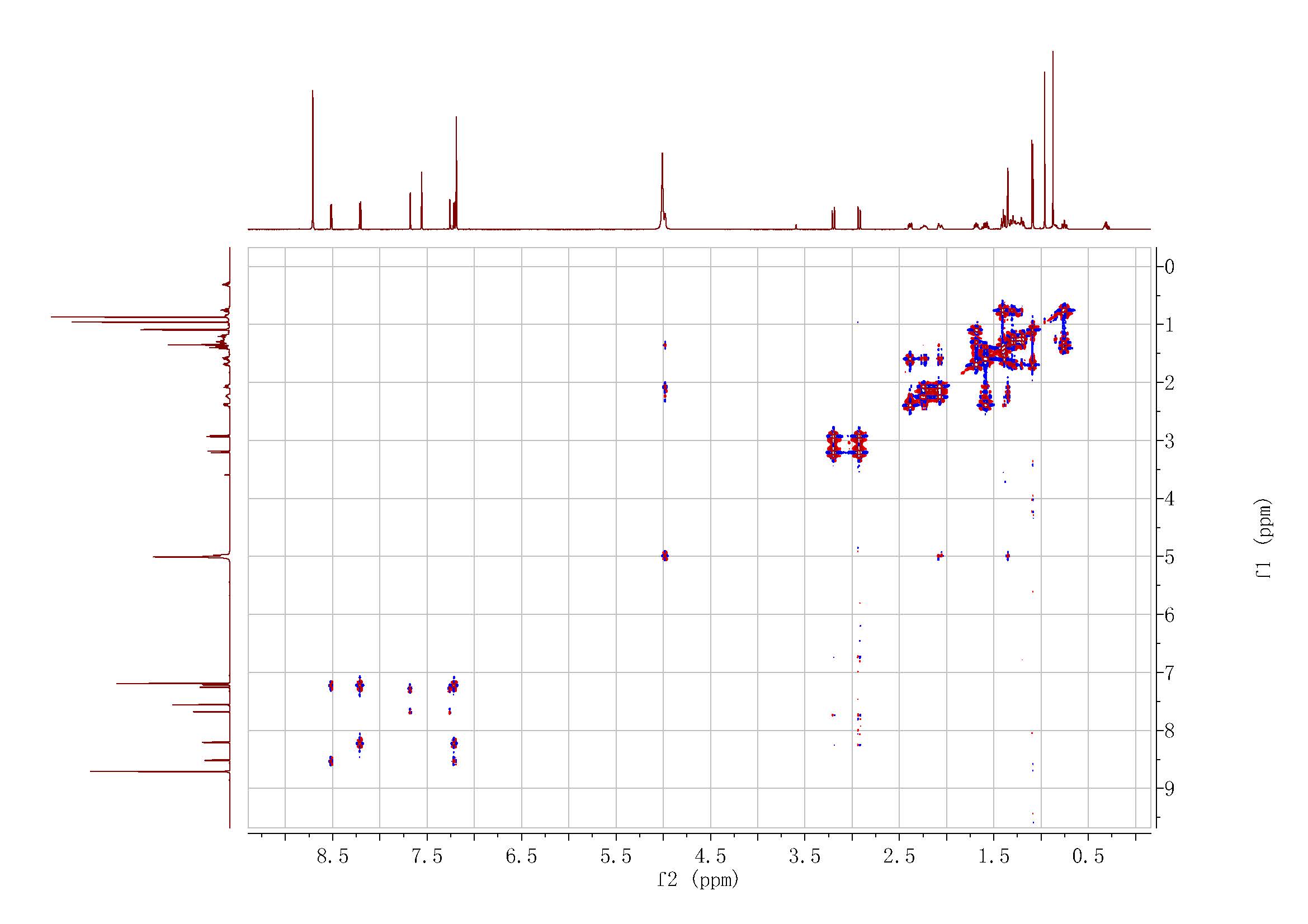


**Figure S27.** ^1^H-^1^H COSY Spectrum of Dysivillosin C (**3**) in Pyr-*d*_5_.


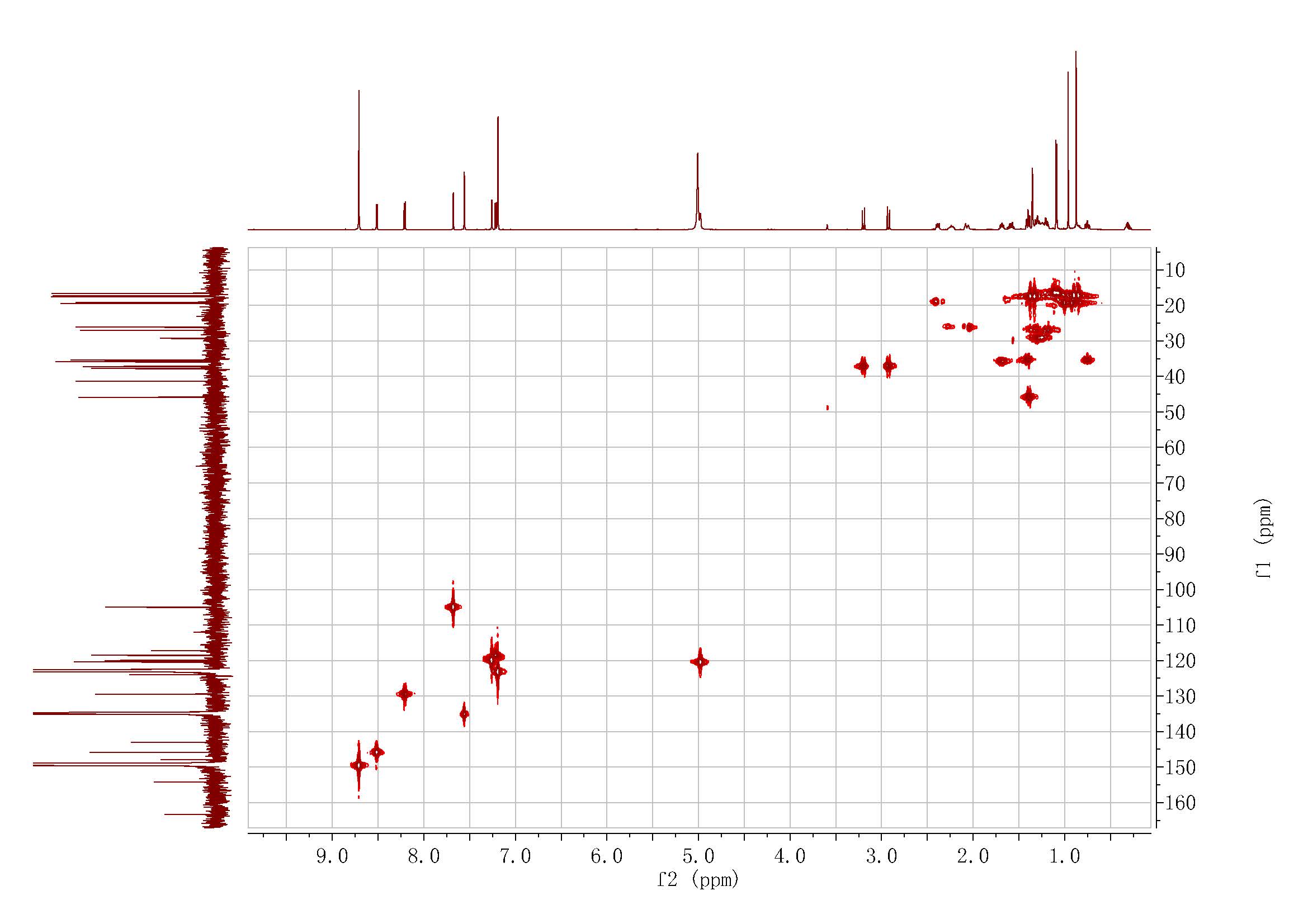


**Figure S28.** HSQC Spectrum of Dysivillosin C (**3**) in Pyr-*d*_5_.


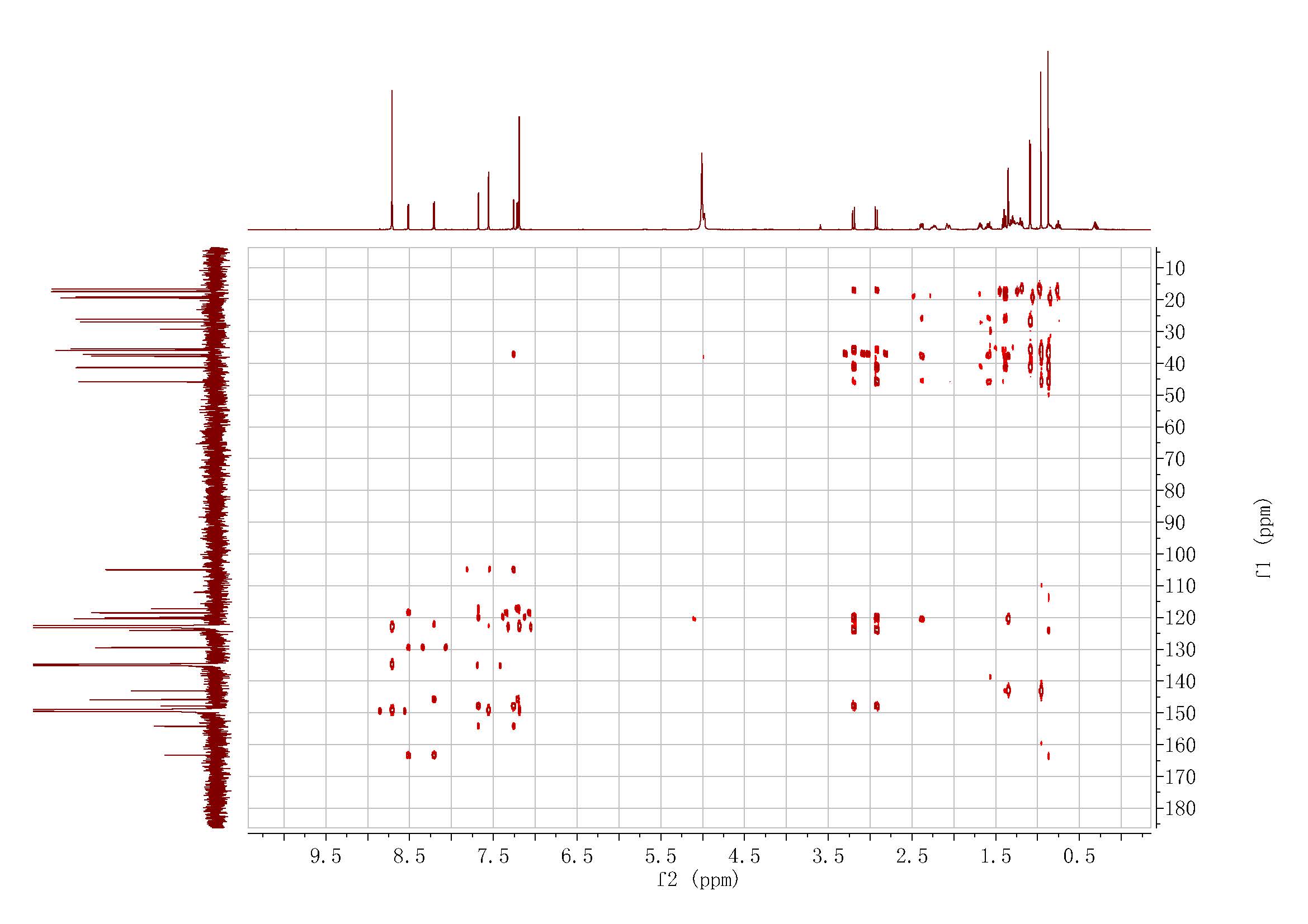


**Figure S29.** HMBC Spectrum of Dysivillosin C (**3**) in Pyr-*d*_5_.


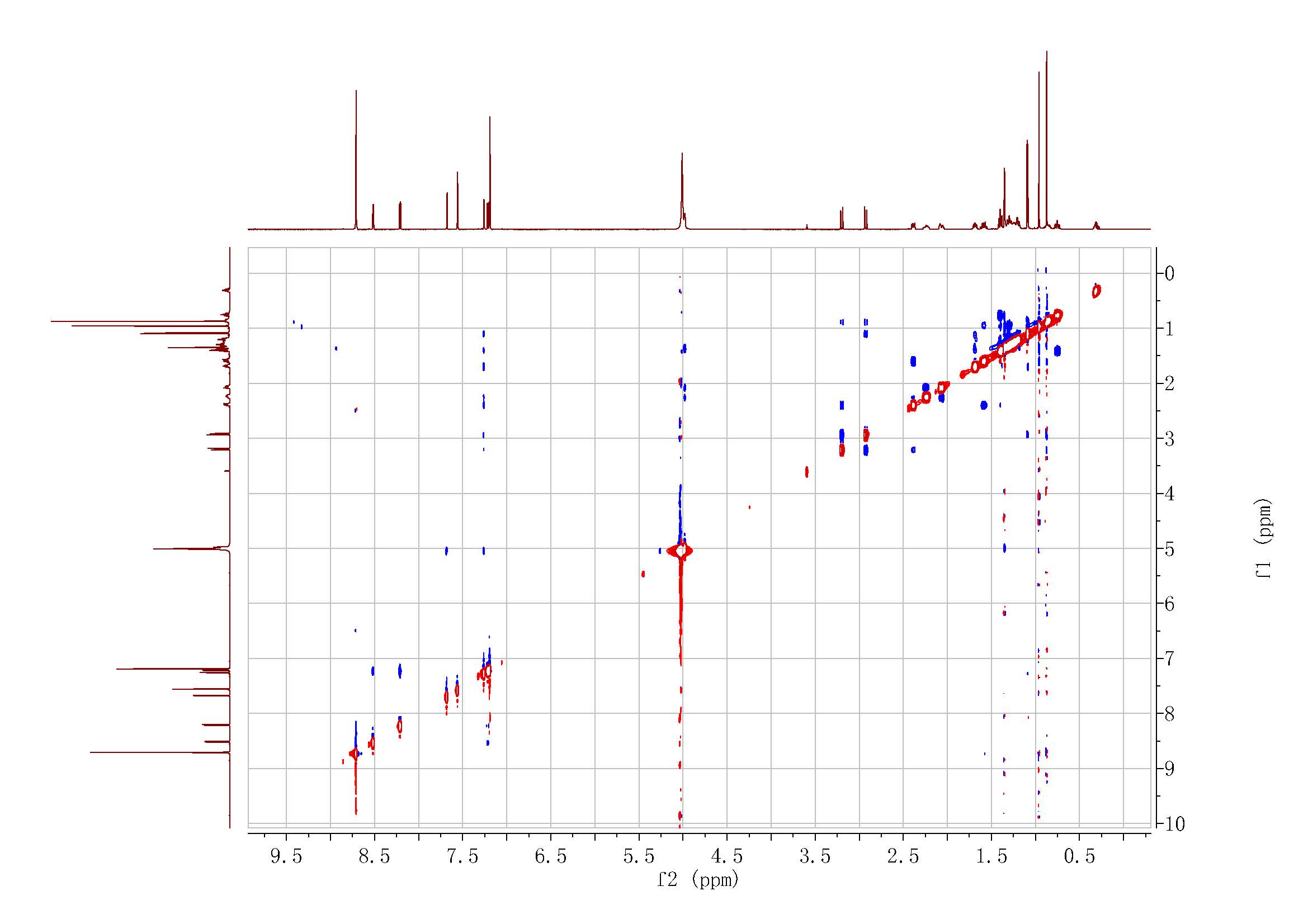


**Figure S30.** NOESY Spectrum of Dysivillosin C (**3**) in Pyr-*d*_5_.


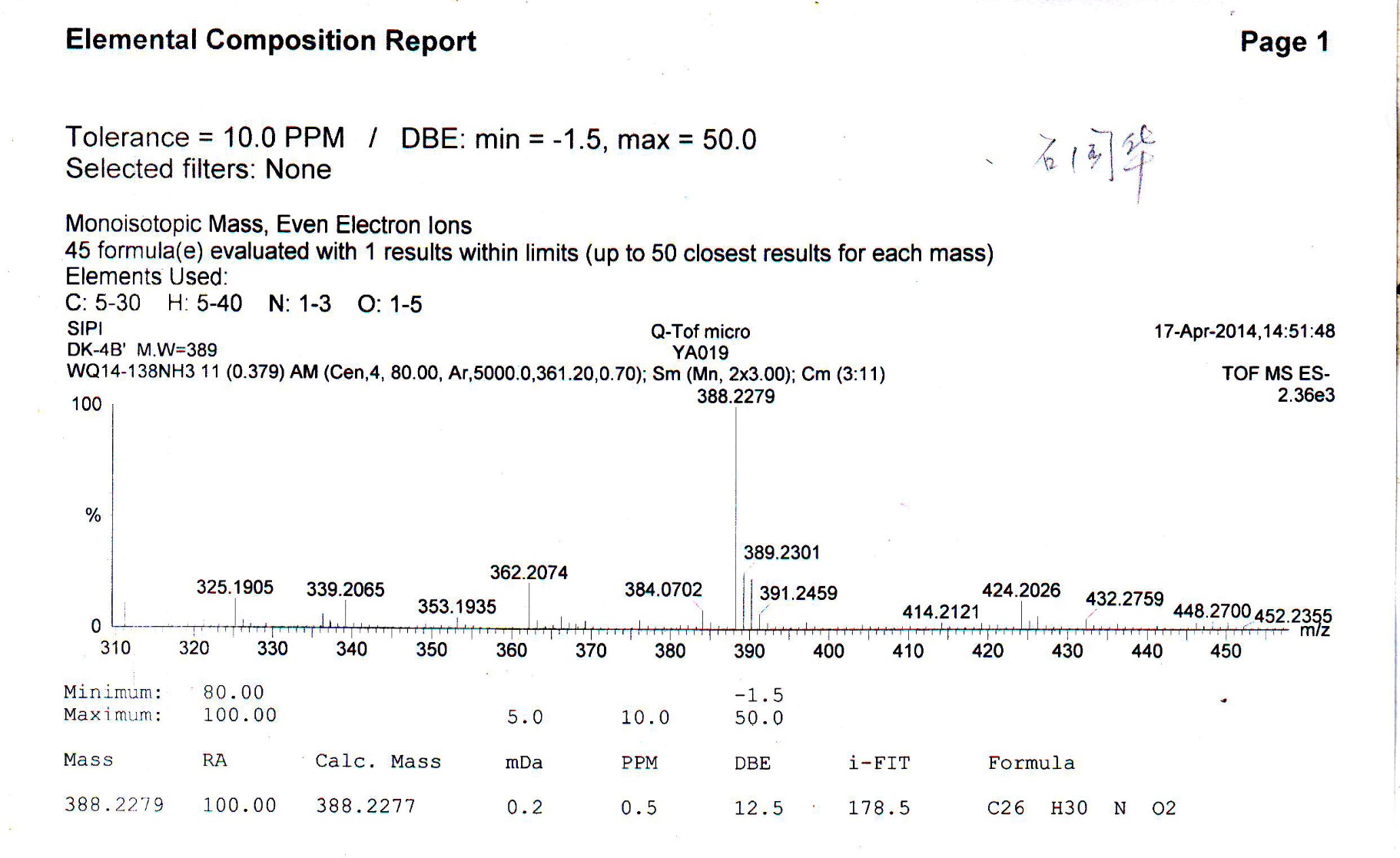


**Figure S31.** HRESIMS of Dysivillosin C (**3**).


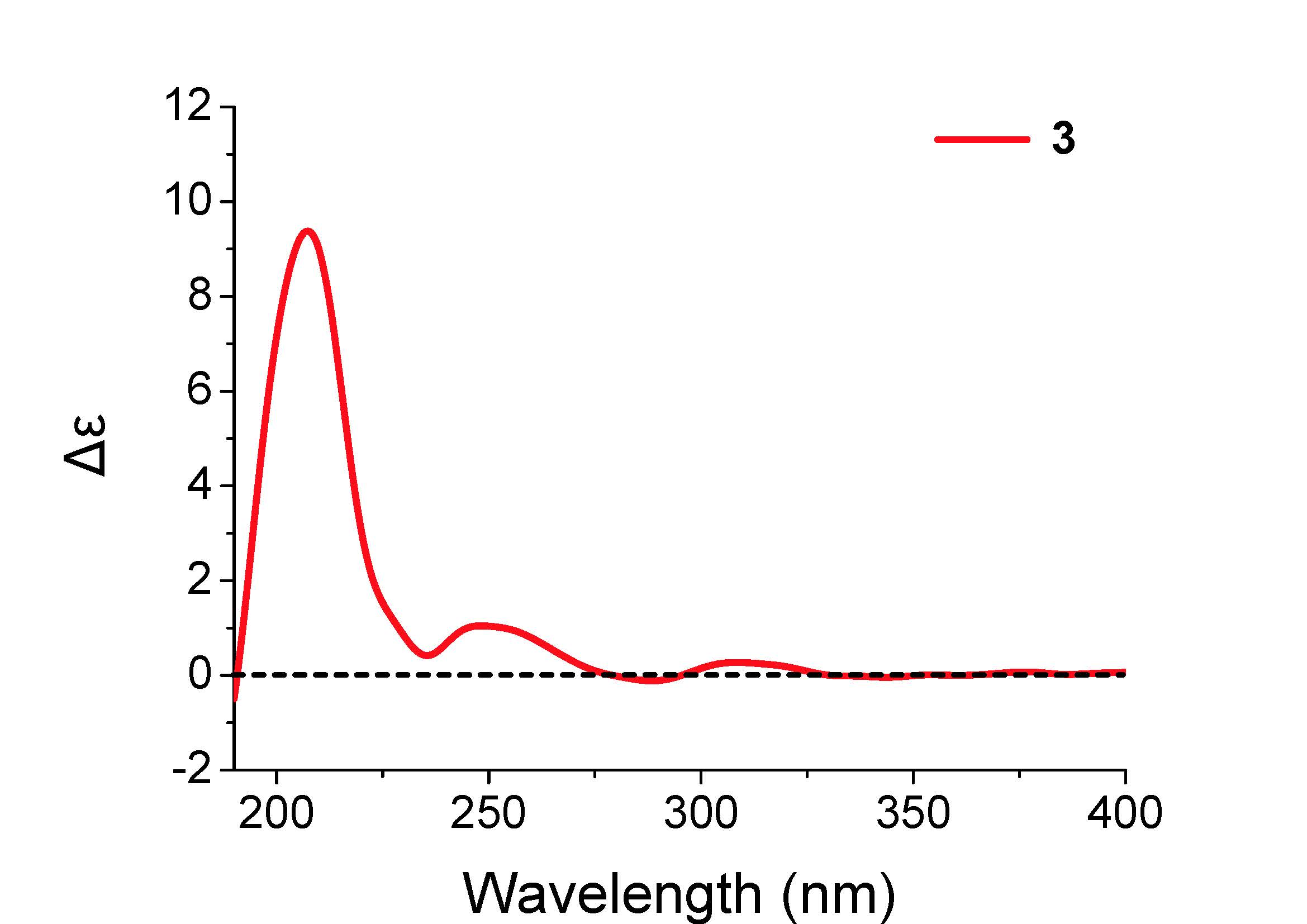


**Figure S32.** Experimental ECD Spectrum of Dysivillosin C (**3**) in MeOH.


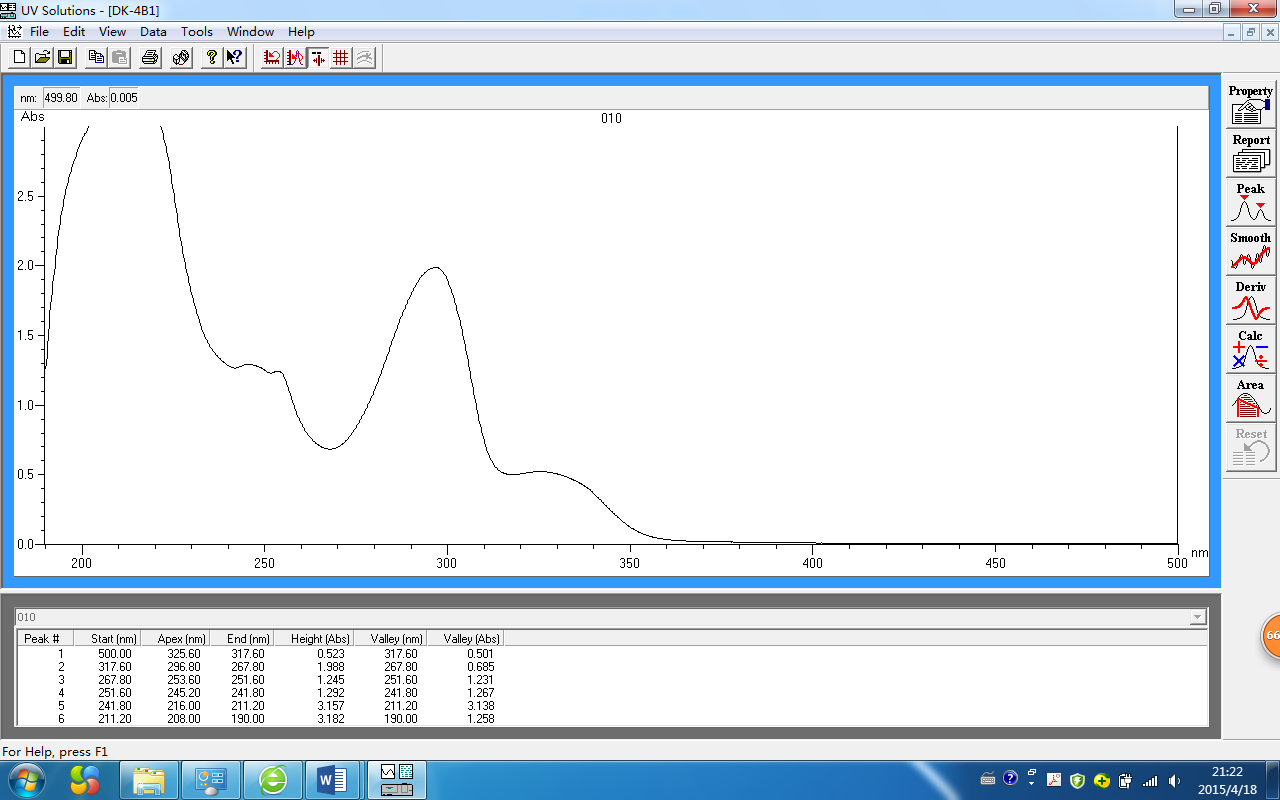


**Figure S33.** UV Spectrum of Dysivillosin C (**3**) in MeOH.


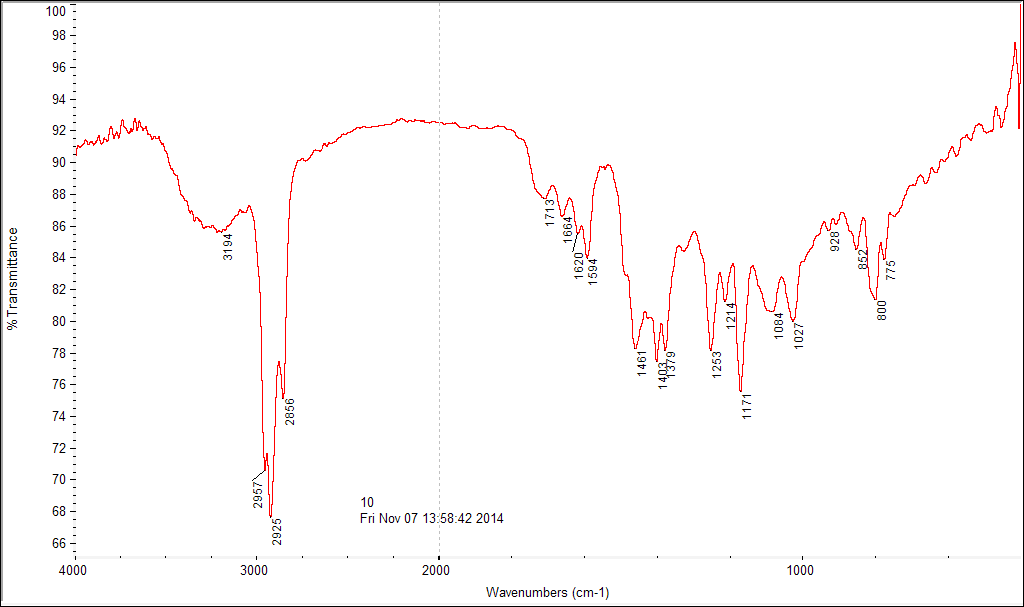


**Figure S34.** IR Spectrum of Dysivillosin C (**3**).


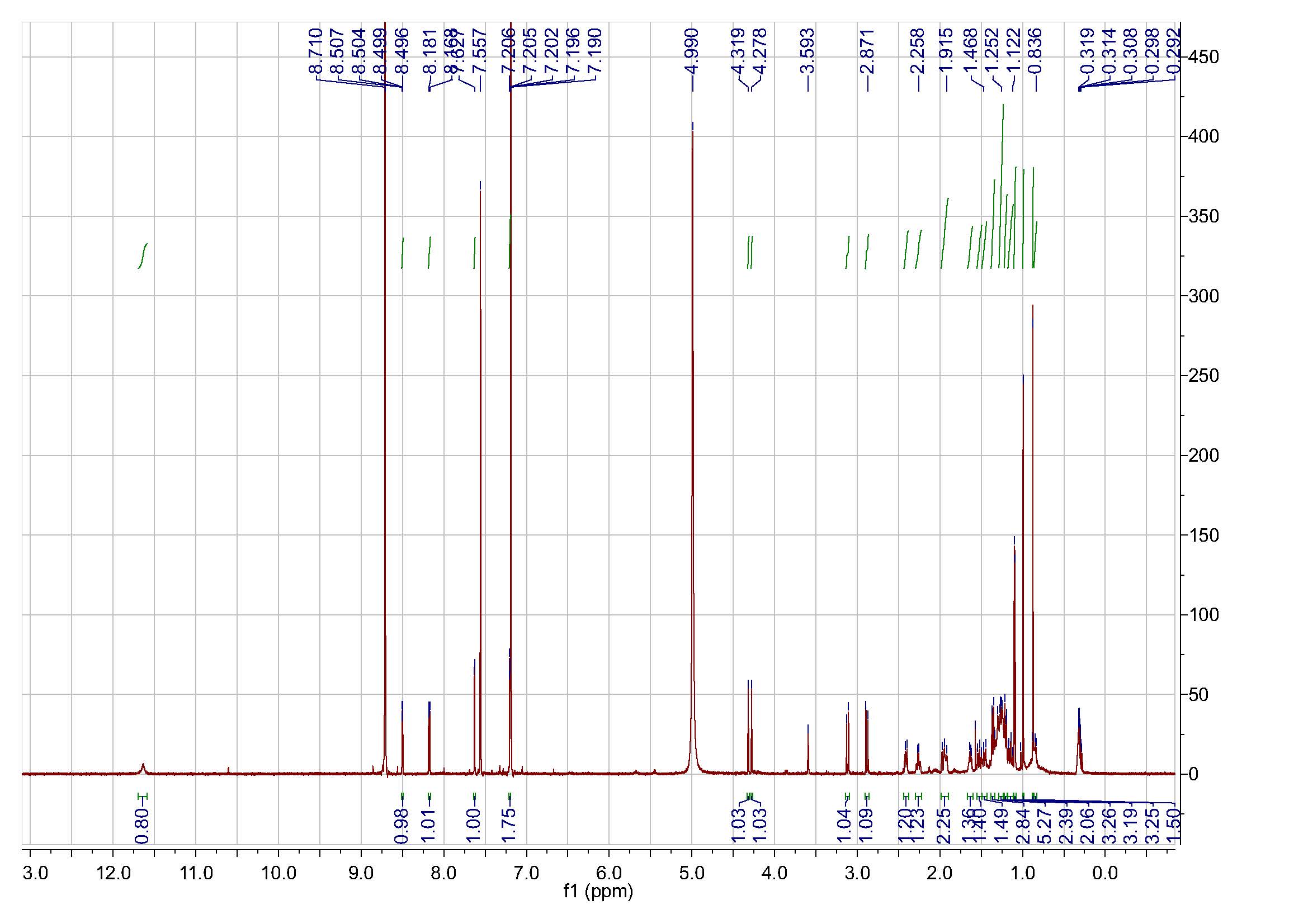


**Figure S35.** ^1^H NMR Spectrum of Dysivillosin D (**4**) in Pyr-*d*_5_.


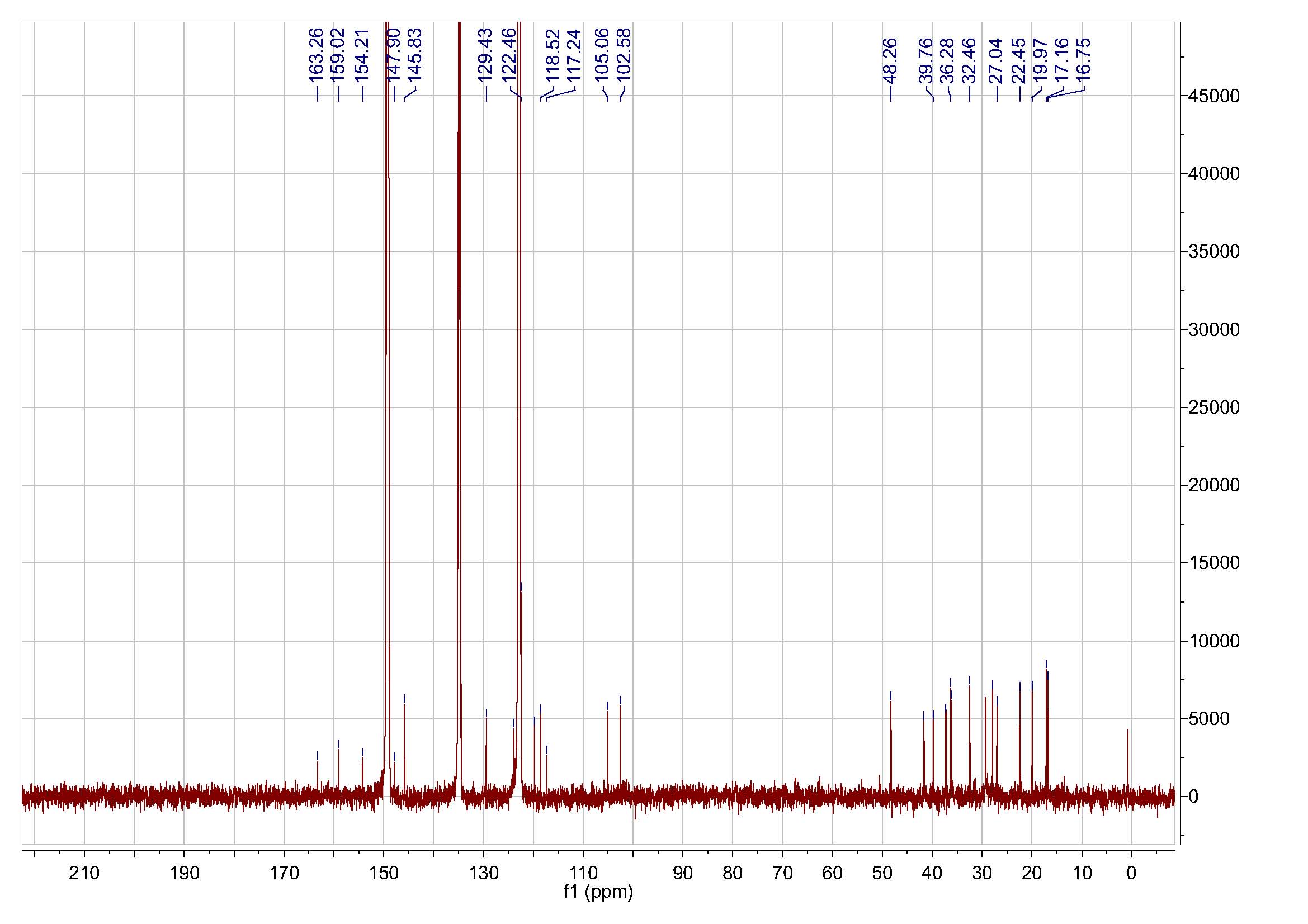


**Figure S36.** ^13^C NMR Spectrum of Dysivillosin D (**4**) in Pyr-*d*_5_.


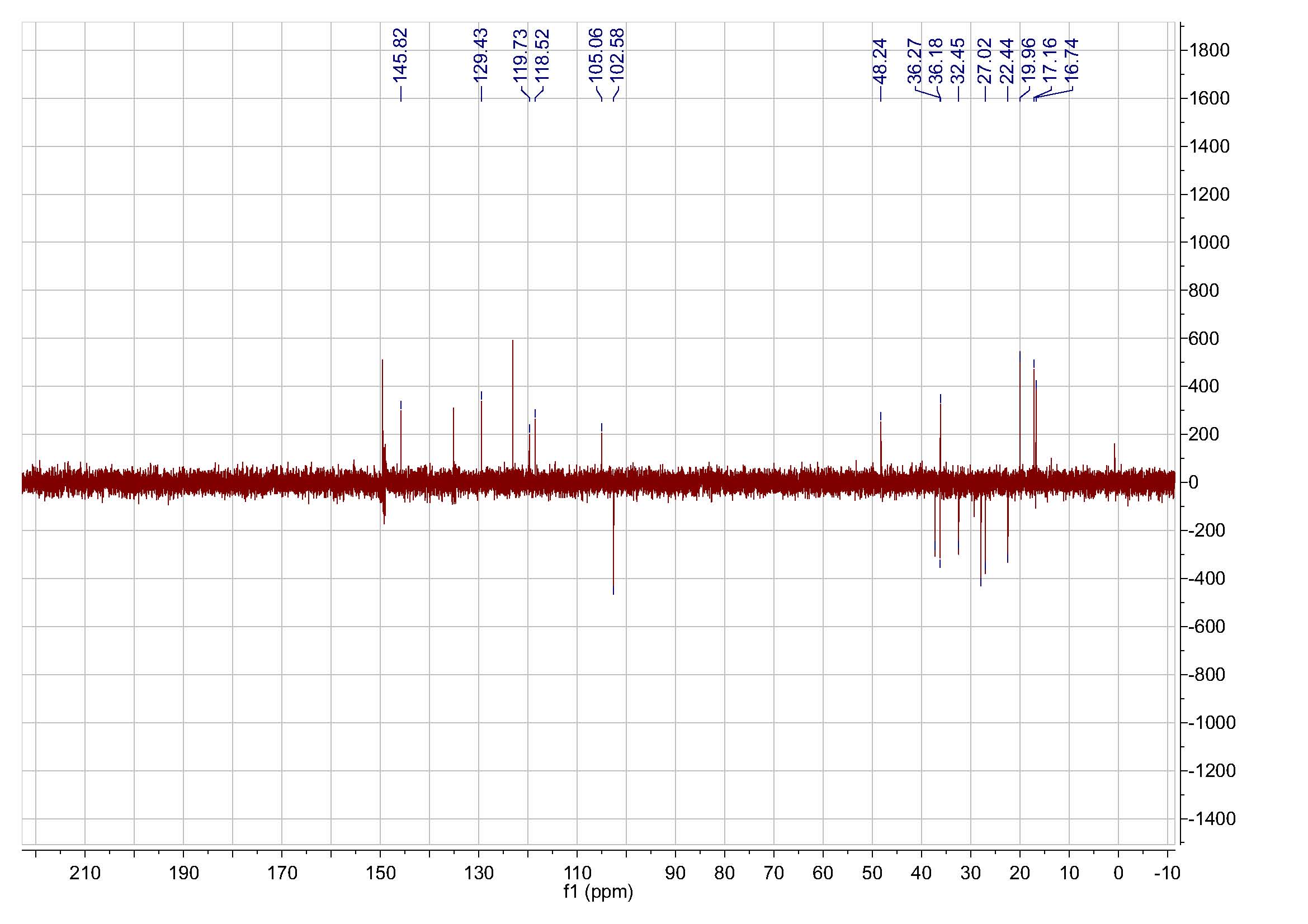


**Figure S37.** DEPT135 Spectrum of Dysivillosin D (**4**) in Pyr-*d*_5_.


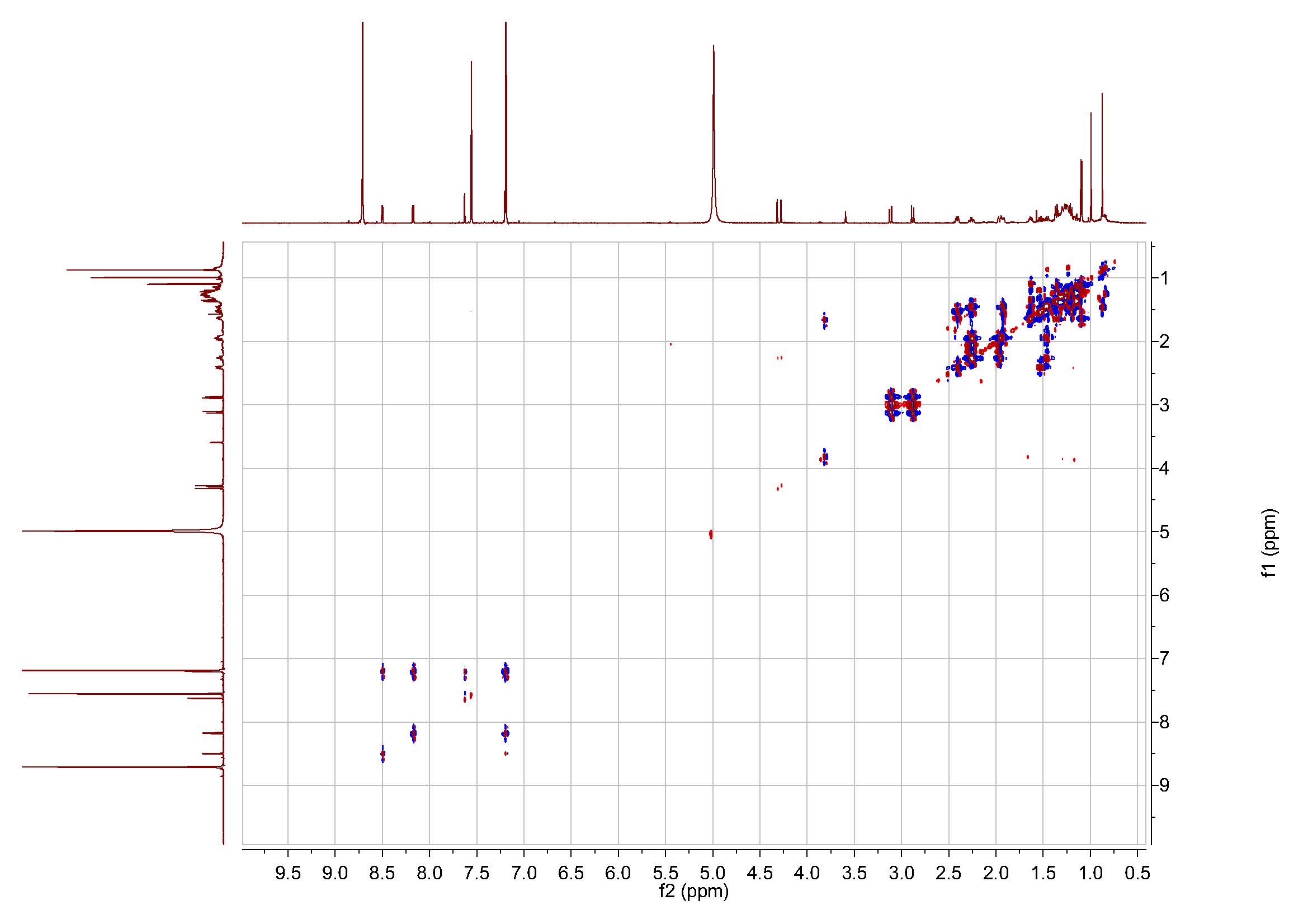


**Figure S38.** ^1^H-^1^H COSY Spectrum of Dysivillosin D (**4**) in Pyr-*d*_5_.


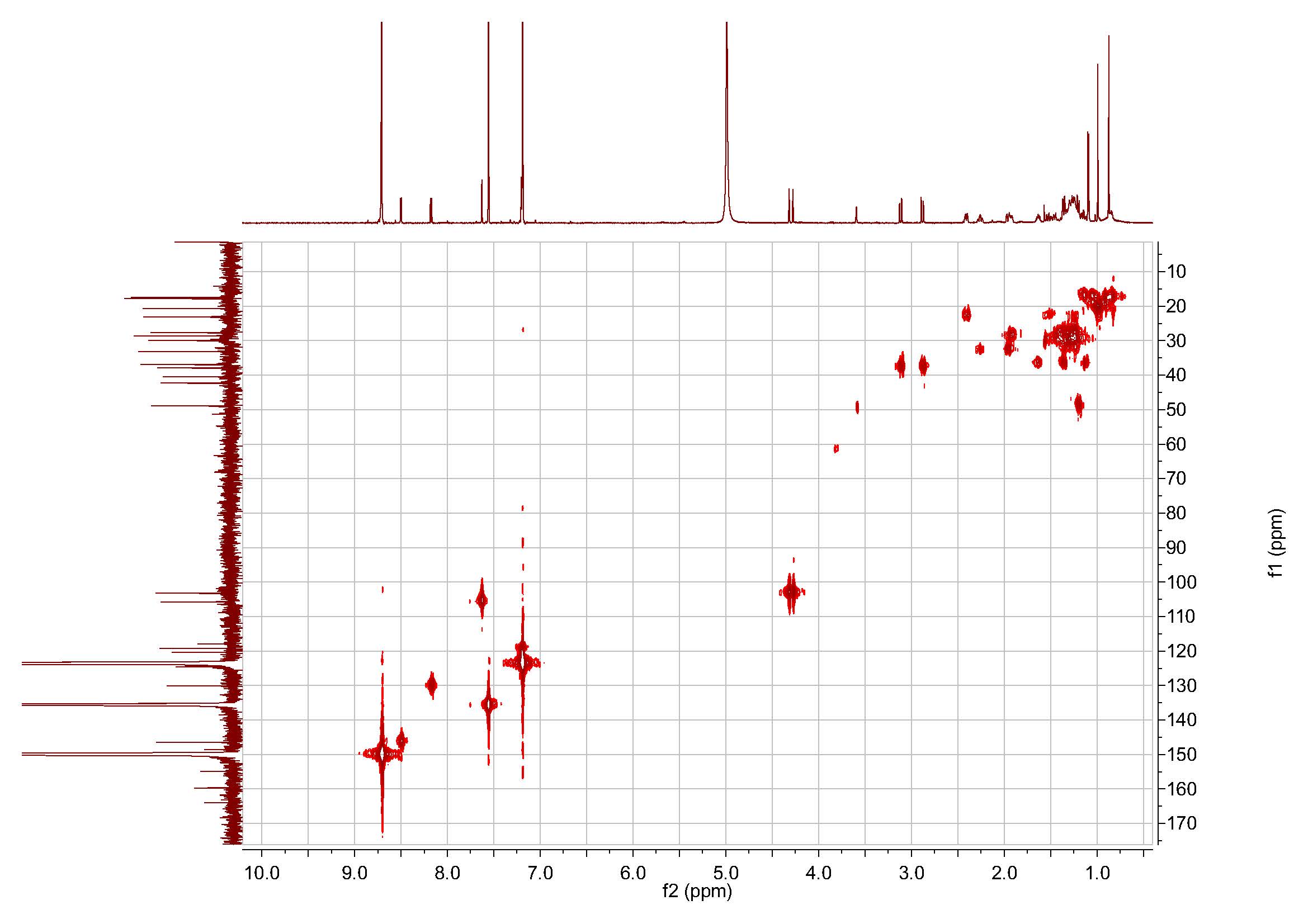


**Figure S39.** HSQC Spectrum of Dysivillosin D (**4**) in Pyr-*d*_5_.


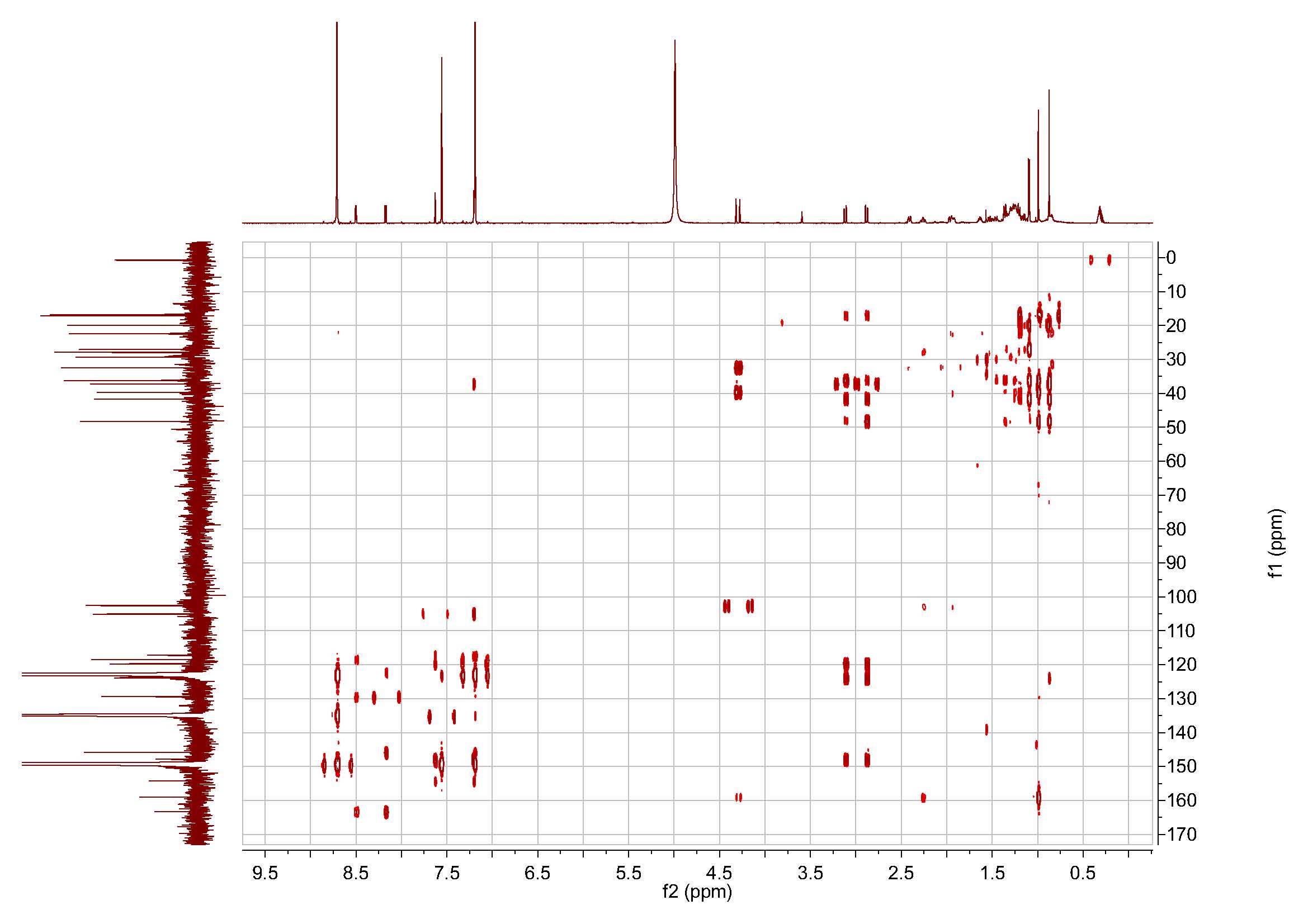


**Figure S40.** HMBC Spectrum of Dysivillosin D (**4**) in Pyr-*d*_5_.


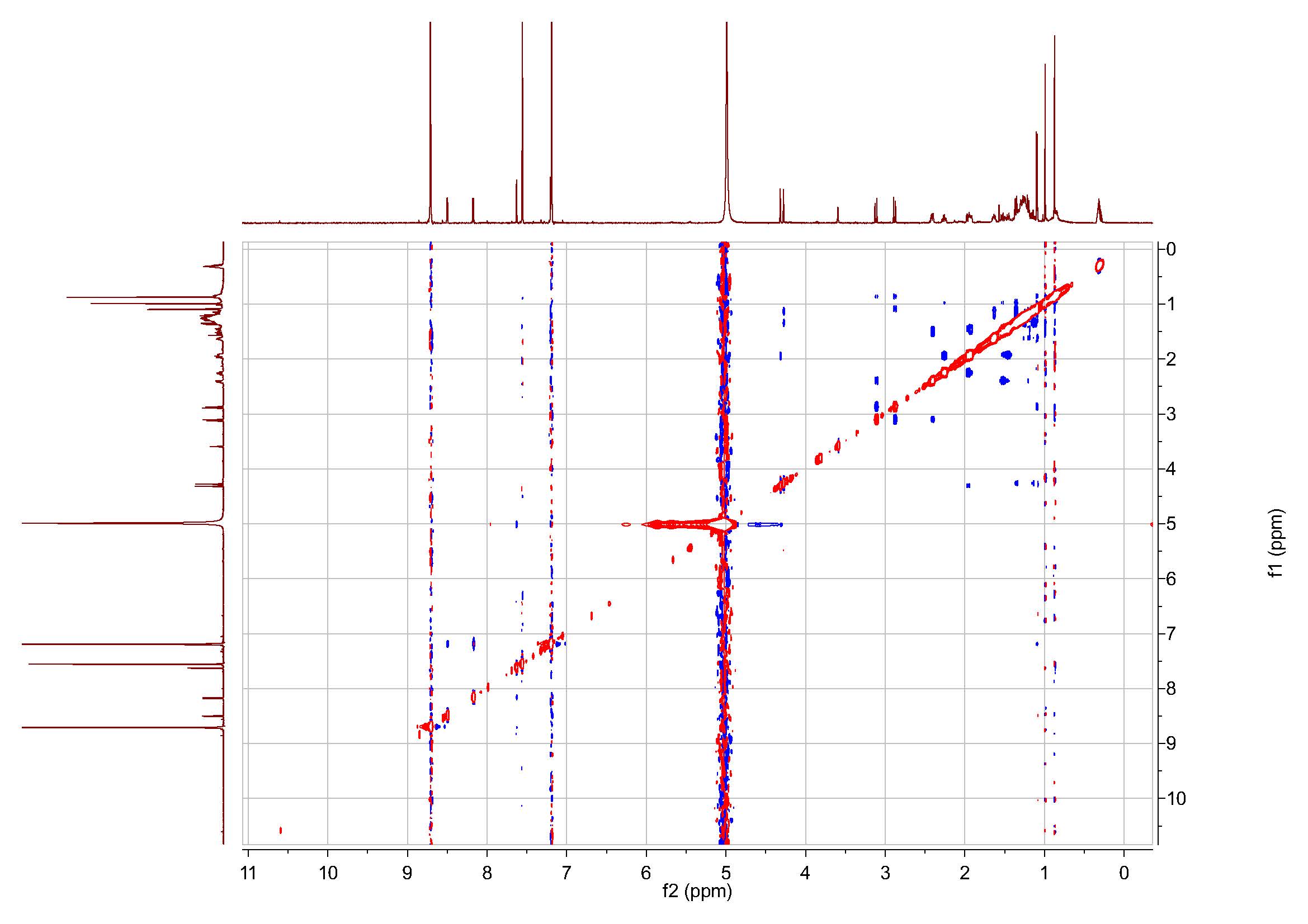


**Figure S41.** NOESY Spectrum of Dysivillosin D (**4**) in Pyr-*d*_5_.


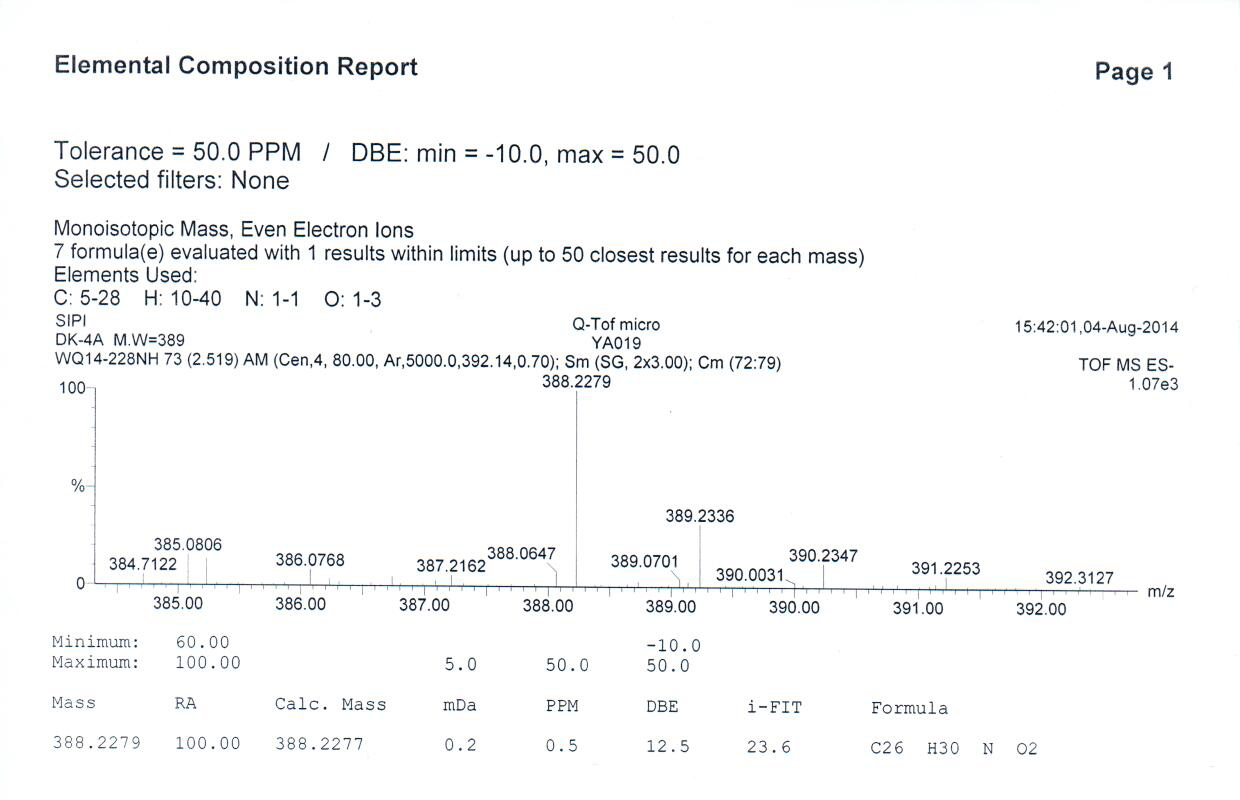


**Figure S42.** HRESIMS of Dysivillosin D (**4**).


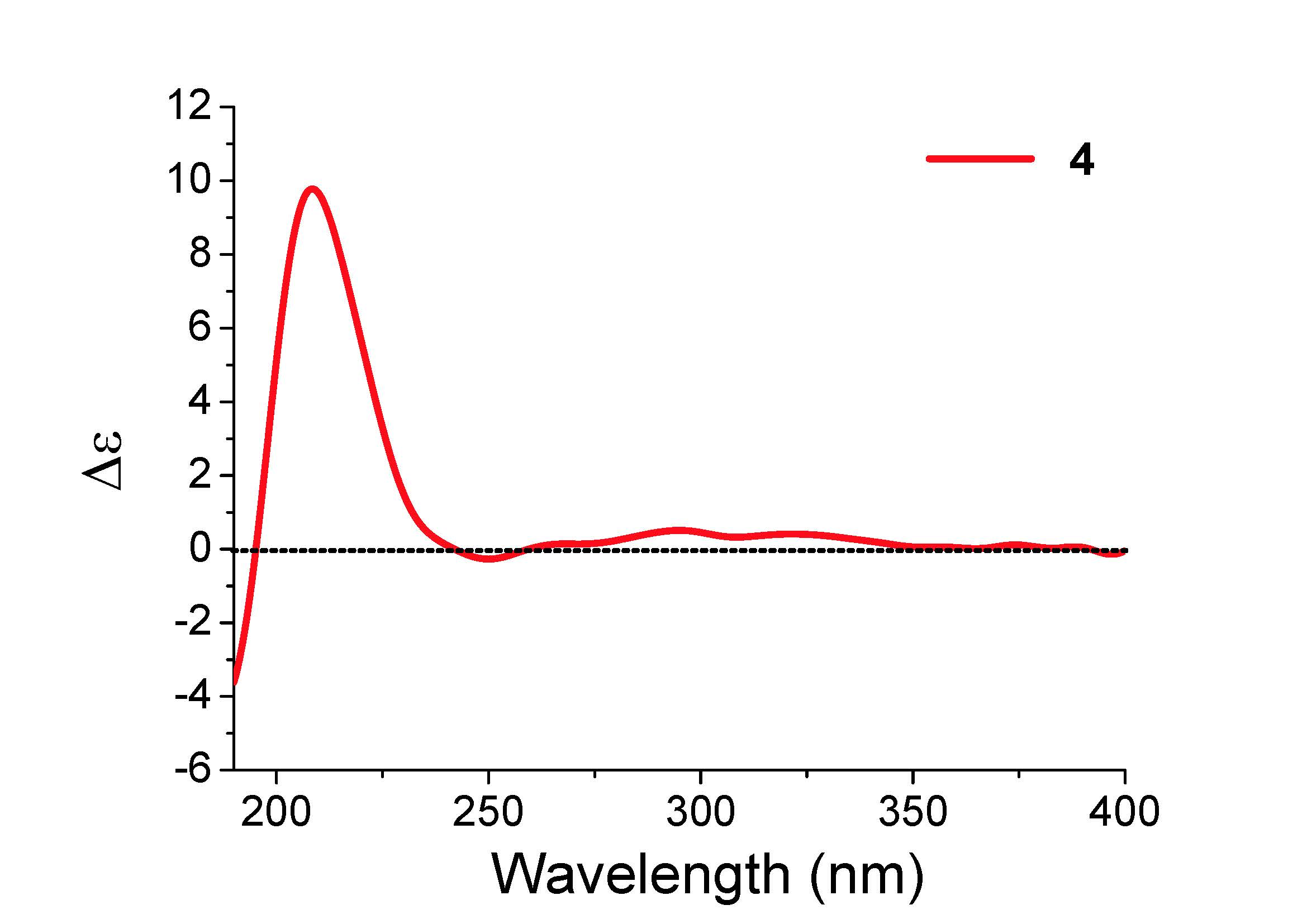


**Figure S43.** Experimental ECD Spectrum of Dysivillosin D (**4**) in MeOH.


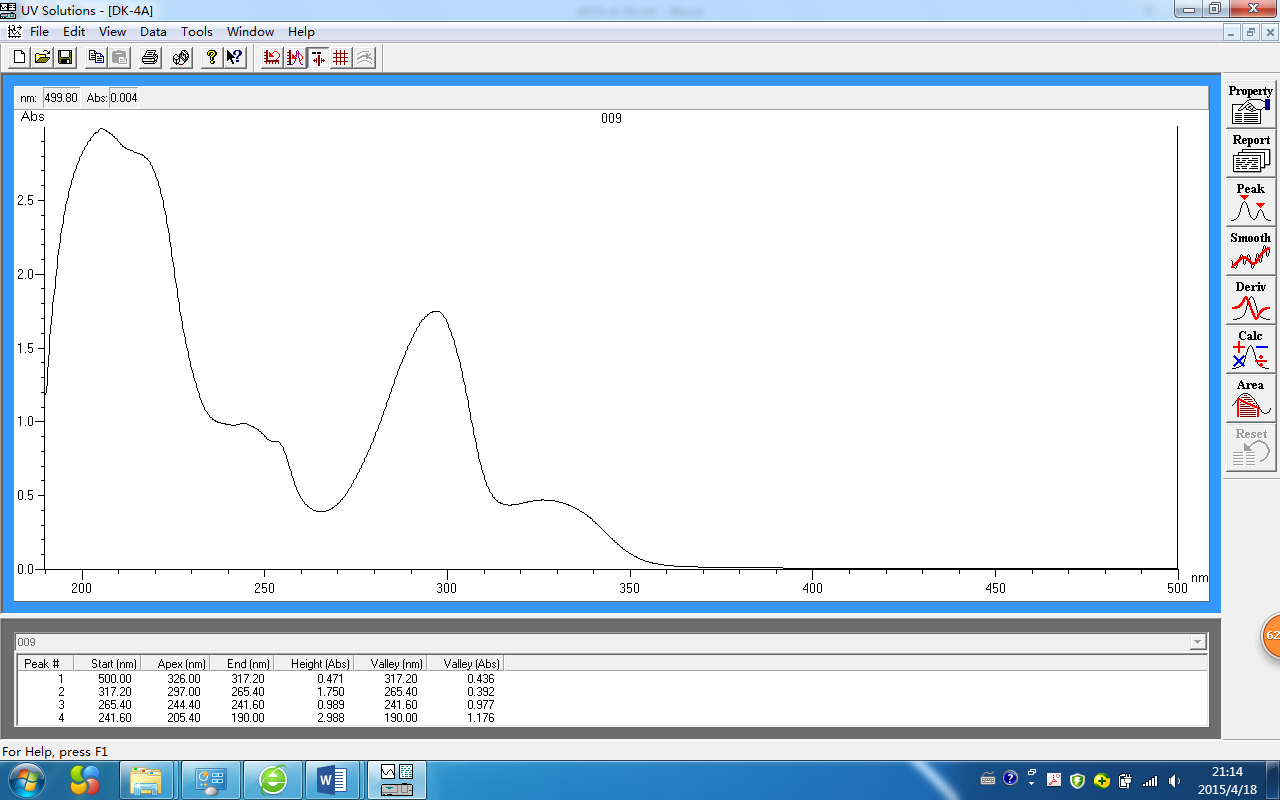


**Figure S44.** UV Spectrum of Dysivillosin D (**4**) in MeOH.


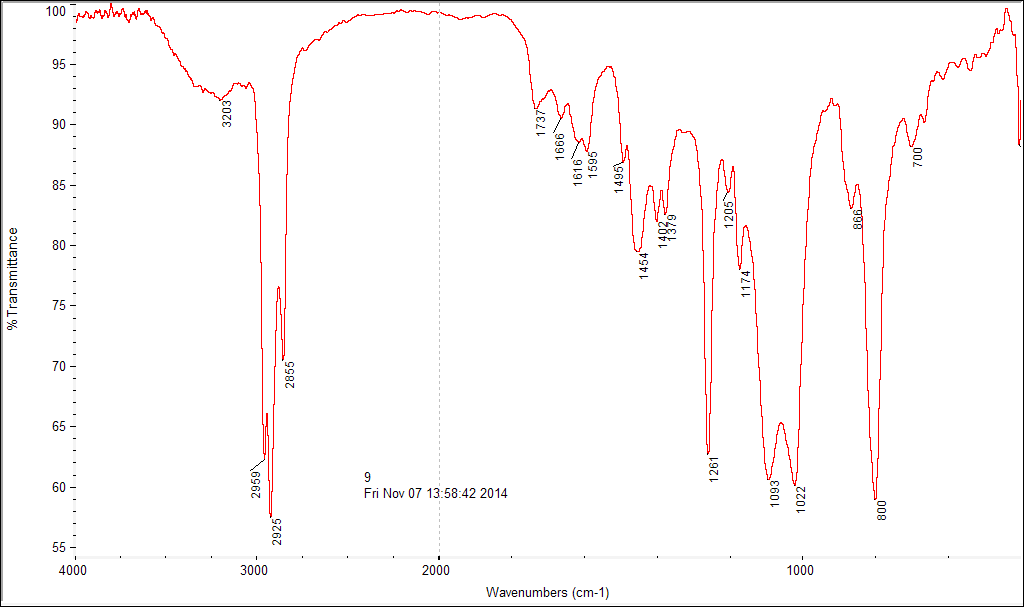


**Figure S45.** IR Spectrum of Dysivillosin D (**4**).
